# Organocatalytic one-pot 1,4-/1,6-/1,2-addition sequence for the stereocontrolled formation of six consecutive stereocenters†
†Electronic supplementary information (ESI) available. CCDC 1037530. For ESI and crystallographic data in CIF or other electronic format see DOI: 10.1039/c4cc09730k



**DOI:** 10.1039/c4cc09730k

**Published:** 2015-01-07

**Authors:** Pankaj Chauhan, Suruchi Mahajan, Gerhard Raabe, Dieter Enders

**Affiliations:** a Institute of Organic Chemistry , RWTH Aachen University , Landoltweg 1 , 52074 Aachen , Germany . Email: enders@rwth-aachen.de ; Fax: +49 241 8092127

## Abstract

An unprecedented stereoselective organocatalytic one-pot 1,4-/1,6-/1,2-addition sequence between β-dicarbonyl compounds, β-nitroalkenes and 4-nitro-5-styrylisoxazoles sequentially catalyzed by low loading of a squaramide catalyst and an achiral base has been developed. The protocol opens an efficient entry to isoxazole bearing cyclohexanes with six consecutive stereogenic centers including one tetrasubstituted carbon in good yields and excellent diastereo- and enantioselectivities.

## Abstract 2

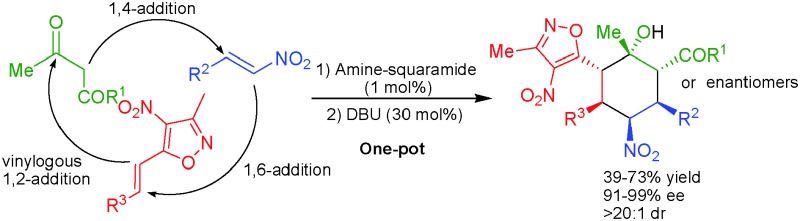
A new stereoselective organocatalyzed one-pot 1,4-/1,6-/1,2-addition reaction sequentially catalyzed by low loading of a squaramide and an achiral base provide a direct entry to isoxazole bearing cyclohexanes with six contiguous stereogenic centers in good yields and excellent stereoselectivities.

Over the last ten years, asymmetric organocatalytic cascade reactions have emerged as a powerful strategy for the synthesis of complex molecules bearing multiple stereogenic centers in a highly stereocontrolled fashion.^1^ These one-pot organocatalytic reactions were successfully employed for the creation of cyclohexane ring systems bearing up to six stereocenters.^2^ Most of these triple cascade reactions are governed by more common 1,4-/1,4-/1,2 addition sequences. Another important class of addition reactions involving the enantioselective 1,6-addition to control the formation of a remote stereocenter is more challenging and less explored in comparison to the other addition variants.^3^ Moreover, organocatalytic cascade reactions using all possible types of addition reactions, *i.e.* 1,4-/1,6-/1,2-addition reactions, are not known so far. Hence we took the challenge to develop a new stereoselective one-pot organocascade sequence using 1,4-/1,6-/1,2-additions (Scheme 1).

**Scheme 1:**
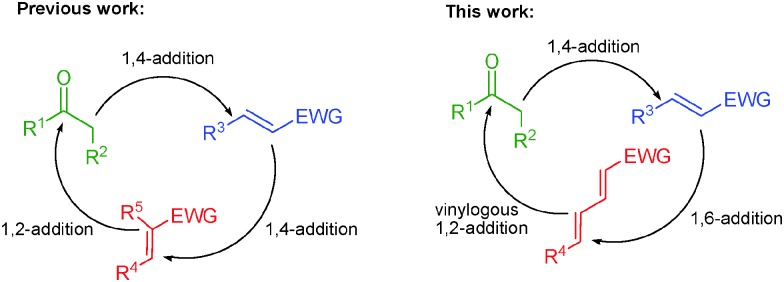
Enantioselective strategies for the construction of cyclohexane rings bearing multiple stereogenic centers.

In addition, the isoxazole core is present in various important naturally occurring and synthetic bioactive molecules (Fig. 1). For example, compounds **A–D** are β-lactamase-resistant antibiotics,^4^ while an isoxazole containing natural product **E** is a powerful neurotoxin, which is used as a brain-lesioning agent.^5^ A synthetic androgenic steroid danazol **D** bearing an isoxazole ring suppresses the production of gonadotrophins and also has some weak androgenic effects.^6^ Moreover, isoxazoles serve as precursors for the synthesis of various synthetically useful organic compounds.^7^ Thus, the development of efficient asymmetric methods for the synthesis of isoxazole ring containing molecules can provide a new series of potentially bioactive molecules.

**Fig. 1:**
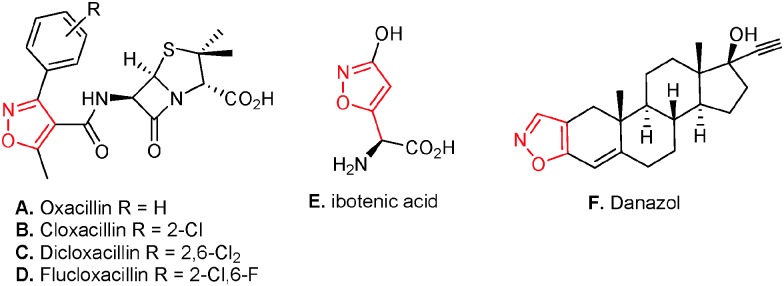
Enantiopure drugs and bioactive natural products bearing an isoxazole ring.

Recently, organo- and metal-catalyzed 1,6-additions to 4-nitro-5-styrylisoxazoles emerged as an efficient method to generate enantiopure isoxazole derivatives bearing one or two stereocenters.^8,9^ However, the 4-nitro-5-styrylisoxazoles remained less explored substrates in stereoselective cascade reactions.^9d,g^ Very recently, Jørgensen's group utilized 4-nitro-5-styrylisoxazoles in trienamine-mediated asymmetric [4+2] cycloaddition reactions to afford cyclohexene products bearing three vicinal stereocenters.^10^ Herein we report a novel cascade reaction involving a 1,4-/1,6-/vinylogous 1,2-addition sequence to access enantiopure cyclohexane rings bearing as many as six contiguous stereogenic centers, sequentially catalyzed by low loading of a cinchona derived squaramide^11^ and an achiral base.

Initially, we started our investigation with a squaramide **I** (1 mol%) catalyzed one-pot three component reaction between ethyl acetoacetate (**1a**), β-nitrostyrene (**2a**) and 4-nitro-5-styrylisoxazole (**3a**) (Table 1, entry 1). However our attempt to obtain the desired cyclohexane ring failed completely, and only the formation of the Michael adduct was observed.^12^ We envisaged that the squaramide catalyst was not enough active to generate a nitronate anion in the corresponding Michael adduct to initiate a domino 1,6-/vinylogous 1,2-addition sequence. Thus, a sequential reaction was performed involving a squaramide **I** catalyzed Michael addition of the β-ketoester **1a** to the β-nitrostyrene **2a**, followed by the addition of **3a** and a catalytic amount of DBU (20 mol%) (entry 2). To our delight, the desired cyclohexane **4a** was obtained in 46% yield with excellent stereoselectivity (98% ee and >20 : 1 dr). Further optimization of the reaction conditions by screening different solvents (entries 3–5) and bases (entries 6–11) showed that 30 mol% of DBU in CH_2_Cl_2_ provides a maximum yield of 62% and excellent stereoselectivity (entry 6). The use of a quinidine derived squaramide catalyst **II** led to the opposite enantiomer of the cyclohexane *ent*-**4a** with a similar yield, ee and dr (entry 12).

**Table 1:** Optimizations of the reaction conditions^*a*^

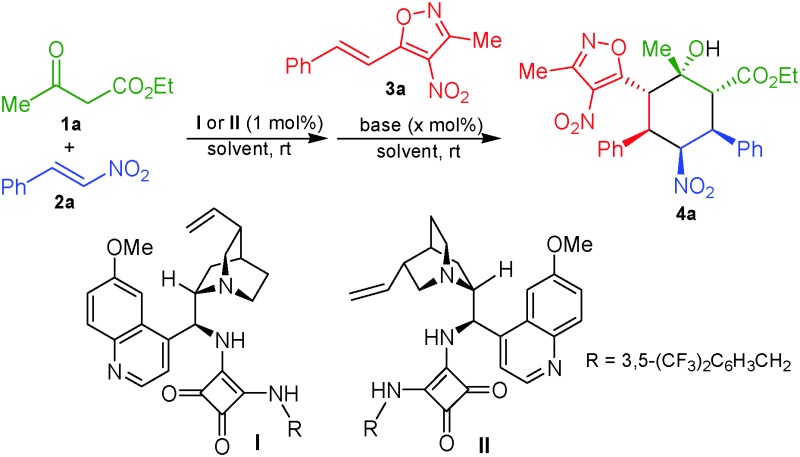					
Entry	Base (*x* mol%)	Solvent	Time^*b*^ (h)	Yield^*c*^ (%)	ee^*d*^ (%)
1^*e*^	—	CH_2_Cl_2_	24	—	—
2	DBU (20)	CH_2_Cl_2_	24 + 24	46	98
3	DBU (20)	CHCl_3_	24 + 24	35	98
4	DBU (20)	Toluene	24 + 24	44	98
5	DBU (30)	THF	24 + 24	36	98
6^*f*^	**DBU (30)**	**CH_2_Cl_2_**	**24 + 48**	**62**	**98**
7^*f*^	DBN (30)	CH_2_Cl_2_	24 + 48	36	97
8^*f*^	TEA (30)	CH_2_Cl_2_	24 + 48	Traces	n.d.
9^*f*^	TBD (30)	CH_2_Cl_2_	24 + 48	29	98
10^*f*^	DABCO (30)	CH_2_Cl_2_	24 + 48	—	—
11^*f*^	Piperidine (30)	CH_2_Cl_2_	24 + 48	Traces	n.d.
12^*f*^	DBU (30)	CH_2_Cl_2_	24 + 48	58	96^*g*^

^*a*^Reaction conditions: 0.2 mmol of **1a**, 0.2 mmol of **2a**, 1 mol% of **I**, 0.24 mmol of **3a** and *x* mol% of base (0.1 M in solvent).

^*b*^Time in hours for both reaction steps.

^*c*^Yield of isolated **4a** after column chromatography.

^*d*^Enantiomeric excess of the major diastereomer (>20 : 1 dr) determined by HPLC analysis on a chiral stationary phase.

^*e*^All the reactants were added in one step.

^*f*^2 equivalents of **3a** were used.

^*g*^ee value of *ent*-**4a** synthesized by using catalyst **II**.

Once equipped with optimized reaction conditions, we evaluated the substrate scope at a 0.5 mmol scale of the β-dicarbonyl compounds and the β-nitrostyrenes (Table 2). The various nitroalkenes bearing electron withdrawing and electron donating groups gave rise to the corresponding isoxazole products **4b–e** in 55–67% yield and excellent stereoselectivities (>20 : 1 dr and 93–99% ee). The nitroalkenes bearing a heteroaromatic group also worked well in this cascade sequence to provide the desired product **4f** in 61% yield and 91% ee. Further screening of different 4-nitro-5-styrylisoxazoles bearing electron withdrawing and electron releasing substituents on the aryl ring as well as heteroaryl group provided a direct access to the corresponding cyclohexanes **4g–m** in good yields and high enantioselectivities (95–99% ee). The methyl acetoacetate and acetyl acetone were also tolerated under this one-pot protocol to give rise to the respective products **4n** and **4o** in good yields and excellent stereoselectivities. Employing a pseudo-enantiomeric amino-squaramide catalyst **II** successfully led to the formation of the enantiomers of **4a–f**, **4h** and **4l** in very good yields (51–69%) and again excellent asymmetric inductions (>20 : 1 dr and 95–98% ee).

**Table 2:** Substrate scope^*a*^

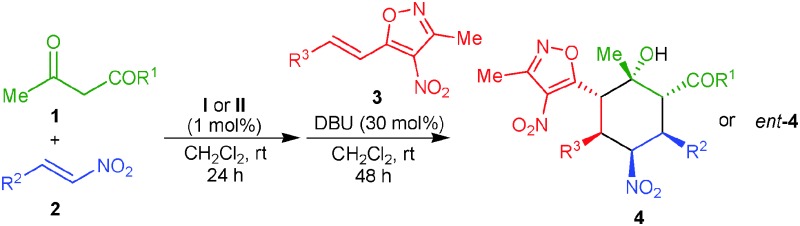					
**4**/*ent*-**4**	R^1^	R^2^	R^3^	Yield^*b*^ (%)	ee^*c*^ (%)
**4a**	OEt	Ph	Ph	61	98
**4b**	OEt	4-FC_6_H_4_	Ph	64	99
**4c**	OEt	4-ClC_6_H_4_	Ph	55	99
**4d**	OEt	4-MeC_6_H_4_	Ph	63	93
**4e**	OEt	4-MeOC_6_H_4_	Ph	67	97
**4f**	OEt	2-Thienyl	Ph	61	91
**4g**	OEt	Ph	4-FC_6_H_4_	60	98
**4h**	OEt	Ph	4-ClC_6_H_4_	61	97
**4i**	OEt	Ph	3-ClC_6_H_4_	69	97
**4j**	OEt	Ph	4-MeC_6_H_4_	73	99
**4k**	OEt	Ph	2-MeC_6_H_4_	49	95
**4l**	OEt	Ph	4-MeOC_6_H_4_	39	96
**4m**	OEt	Ph	2-Thienyl	50	97
**4n**	OMe	Ph	Ph	58	97
**4o**	Me	Ph	Ph	50	96
*ent*-**4a**	OEt	Ph	Ph	69	96
*ent*-**4b**	OEt	4-FC_6_H_4_	Ph	63	97
*ent*-**4c**	OEt	4-ClC_6_H_4_	Ph	51	95
*ent*-**4d**	OEt	4-MeC_6_H_4_	Ph	64	98
*ent*-**4e**	OEt	4-MeOC_6_H_4_	Ph	66	95
*ent*-**4f**	OEt	2-Thienyl	Ph	59	96
*ent*-**4h**	OEt	Ph	4-ClC_6_H_4_	60	97
*ent*-**4k**	OEt	Ph	2-MeC_6_H_4_	50	96

^*a*^Reaction conditions: 0.5 mmol of **1**, 0.5 mmol of **2**, 1 mol% of **I** (entry 1–17) or **II**, 1.0 mmol of **3** and 30 mol% of DBU (0.1 M in CH_2_Cl_2_).

^*b*^Yield of isolated product after column chromatography.

^*c*^Enantiomeric excess of the major diastereomer determined by HPLC analysis on a chiral stationary phase.

The absolute configuration of the products **4a–o** can be assigned as (1*S*), (2*S*), (3*R*), (4*S*), (5*S*) and (6*R*) on the basis of the X-ray crystallographic analysis of **4a** (Fig. 2).^13^

**Fig. 2:**
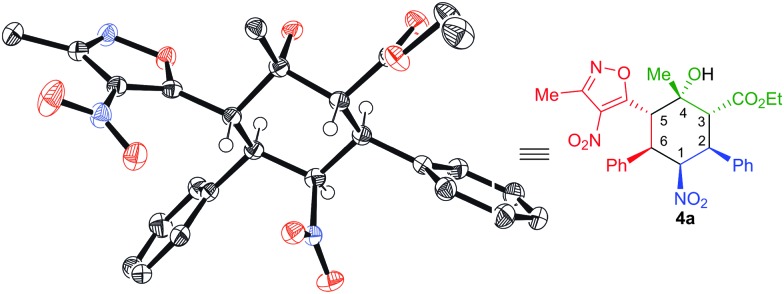
X-ray structure of **4a**.

To demonstrate the practical and preparative application of this new organocascade 1,4-/1,6-/1,2-addition sequence, we performed a gram-scale reaction between **1a**, **2a** and **3a** using a lower loading (0.5 mol%) of the squaramide **I** (Scheme 2). The desired product **4a** was obtained in 57% yield with unchanged ee and dr values. The enantiomeric purity could be enriched to >99% ee after a single crystallization of the product.

**Scheme 2:**
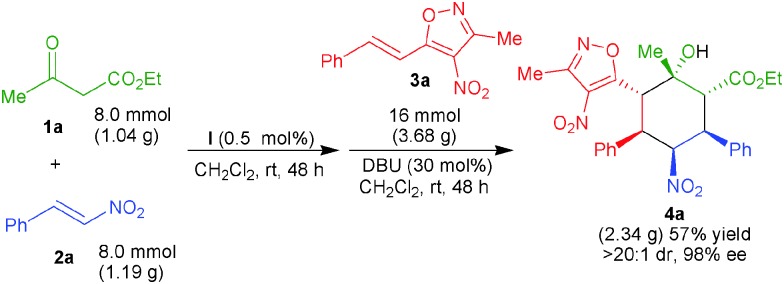
Gram-scale 1,4-/1,6-/1,2-addition sequence.

In conclusion, we have developed a novel 1,4-/1,6-/1,2-addition cascade sequence catalyzed sequentially by low loading of a cinchona-derived squaramide and a commercially available achiral base to afford a series of highly substituted cyclohexane derivatives bearing six consecutive stereogenic centers in good yields and excellent stereoselectivities. The enantiomeric cyclohexanes are also easily synthesized on a same level of asymmetric induction by employing a pseudo-enantiomeric squaramide catalyst. A successful gram-scale reaction documents the preparative utility of this organocascade protocol.

Support from the European Research Council (ERC Advanced Grant 320493 “DOMINOCAT”) is gratefully acknowledged. We thank BASF SE for the donation of chemicals.
