# Supplementary material for: Organocatalytic one-pot 1,4-/1,6-/1,2-addition sequence for the stereocontrolled formation of six consecutive stereocenters
Source: Chem Commun (Camb). 2015 Jan 7;51(12):2270–2. doi: 10.1039/c4cc09730k (PMC4612129; doi:10.1039/c4cc09730k)
Supplement: Supplementary file 1 [file CC-051-C4CC09730K-s001.pdf]

Electronic Supplementary Material (ESI) for ChemComm.
This journal is © The Royal Society of Chemistry 2014

Organocatalytic one-pot 1,4-/1,6-/1,2-addition sequence for the stereocontrolled formation of six consecutive stereocenters

Pankaj Chauhan, Suruchi Mahajan, Gerhard Raabe and Dieter Enders*

[a] Institut of Organic Chemistry, RWTH Aachen University Landoltweg 1, 52074 Aachen (Germany)
E-mail: enders@rwth-aachen.de

Contents

General Methods and Materials	1
General Procedure for the organocatalytic one-pot 1,4-/1,6-/1,2-additon reactions	1
Analytic data	2-13
Procedure for the gram-scale organocatalytic one-pot 1,4-1,6-/1,2-additon reaction	13
NMR spectra	14-30
HPLC Data	31-45

General Methods and Materials:

All reactions were performed in oven-dried glassware. Analytical TLC was performed using SIL G-25 UV254 from MACHERY & NAGEL and visualized with ultraviolet radiation at 254 nm. ^{1}H and ^{13}C NMR spectra were recorded at ambient temperature on a Varian Innova 600 or Varian Innova 400 instruments with tetramethylsilane as the internal standard. Chemical shifts for ^{1}H-NMR and ^{13}C-NMR are reported in parts per million (ppm), with coupling constants reported in Hertz (Hz). The following abbreviations are used for spin multiplicity: s = singlet, br s = broad singlet, d = doublet, dd = doublet of doublet, t = triplet and m = multiplet. Mass spectra were acquired on a Finnigan SSQ7000 (EI 70 eV) spectrometer and high resolution ESI spectra on a ThermoFisher Scientific LTQ-Orbitrap XL. IR spectra were taken on a PerkinElmer Spectrum 100 FT-IR Spectrometer. Elemental analyses were performed with a Vario EL elemental analyzer. Analytical HPLC was carried out either on a Hewlett-Packard 1050 Series instrument or Agilent 1100 instrument using chiral stationary phases. Optical rotation values were measured on a Perkin-Elmer 241 polarimeter.

Starting materials and reagents were purchased directly from commercial suppliers and used without further purifications. All solvents used as reaction medium were distilled before the use. The nitroalkenes **2**[1] and 4-nitro-5-styrylisoxazoles **3**[2] were synthesized using known literature procedures. The chiral squaramides **I** and **II** were also synthesized using known literature procedures.[3]

General Procedure for the organocatalytic one-pot 1,4-/1,6-/1,2-additon reactions:

In a 10 mL round bottom flask equipped with a magnetic stirring bar, the nitroalkene **2** (1.0 equiv., 0.5 mmol) and catalyst **I** or **II** (1 mol%) were dissolved in CH_2Cl_2 (1.00 mL) and stirred 5 minutes at room temperature followed by the addition of the β-keto ester **1** (1.0 equiv. 0.5 mmol). After stirring the reaction mixture at room temperature for 24 hours the 4-nitro-5-styrylisoxazoles **3** (2 equiv., 1.0 mmol) and DBU (30 mol%; 0.1 M in CH_2Cl_2) were added subsequently and stirred for another 48 hours at room temperature. The crude product was directly purified by flash column chromatographies (first *n*-hexane/EtOAc = 9:1, then *n*-hexane/EtOAc = 4:1) to afford the polysubstituted cyclohexanes **4a-o**.

---

[1] (a) Organic Syntheses, Coll. *Vol. 1,* p.413 (**1941**); *Vol. 9,* p.66 (**1929**); (b) B. M. Trost and C. Müller, *J. Am. Chem. Soc.* 2008, **130**, 2438.
[2] (a) M. F. A. Adamo and E. F. Duffy, *Org. Lett.,* 2006, **8**, 5157; (b) J.-L. Zhang, X.-H. Liu, X.-J. Ma and R. Wang, *Chem. Commun.,* 2013, **49**, 9329.
[3] J. P. Malerich, K. Hagihara and V. H. Rawal, *J. Am. Chem. Soc.,* 2008, **130,** 14416.

Analytic data:

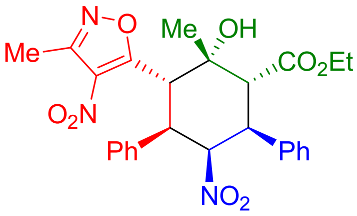

4a

Compound **4a** was synthesized with catalyst **I** and isolated as a colorless solid (155 mg, 61%); Mp = 237-238 °C; $[\alpha]_D^{24}$ = +58.0 (c = 0.5, $CHCl_3$); >20:1 dr; 98% ee (major diastereomer); HPLC (major diastereomer): t_R 11.82 min (major), 17.25 min (minor), 214 nm, *n*-heptane/*i*-PrOH, 7:3, 1.00 mL/min, Chiralpak AD column; IR (capillary): 3526, 3064, 3032, 2985, 2903, 2650, 2326, 2226, 2162, 2109, 2071, 2009, 1958, 1898, 1816, 1730, 1595, 1546, 1513, 1445, 1417, 1371, 1343, 1316, 1252, 1216, 1175, 1140, 1077, 1022, 982, 926, 890, 830, 788, 748, 699 cm^{-1}; ^1H NMR (600 MHz, $CDCl_3$, major diastereomer): δ = 7.32-7.27 (m, 3H, ArH), 7.25-7.24 (m, 2H, ArH), 7.18-7.16 (m, 3H, ArH), 7.12-7.11 (m, 2H, ArH), 5.44 (d, *J* = 13.2 Hz, 1H, CH), 5.04 (t, *J* = 4.2 Hz, 1H, CH), 4.46 (dd, *J* = 12.6, 4.2 Hz, 1H), 4.13-4.09 (m, 2H, 2CH), 3.99-3.92 (m, 2H, CH_2), 3.91 (d, *J* = 6.8 Hz, 1H, OH), 2.42 (s, 3H, CH_3), 1.34 (s, 3H, CH_3), 0.91 (t, *J* = 6.0 Hz, 3H, CH_3); ^{13}C NMR (151 MHz, $CDCl_3$, major diastereomer): δ = 174.2, 171.7, 155.0, 135.6, 135.3, 132.6, 129.4, 129.3, 129.0, 128.8, 128.7, 128.3, 127.3, 93.1, 71.9, 61.6, 50.0, 44.4, 44.1, 43.1, 26.9, 13.7, 11.8. ppm; MS (EI): *m/z* 510.5 $[M+1]^+$; HRMS Calcd for $[C_{26}H_{27}N_3O_8+Na]^+$: 532.1690, found: 532.1690.

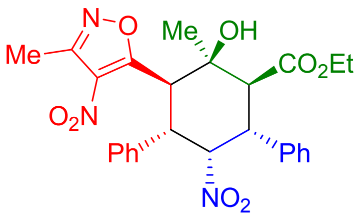

ent-**4a**

Compound *ent*-**4a** was synthesized with catalyst **II** and isolated as a colorless solid (151 mg, 60%); >20:1 dr; 96% ee (major diastereomer); HPLC (major diastereomer): t_R 11.85 min (minor), 17.28 min (major), 214 nm, *n*-heptane/*i*-PrOH, 7:3, 1.00 mL/min, Chiralpak AD column.

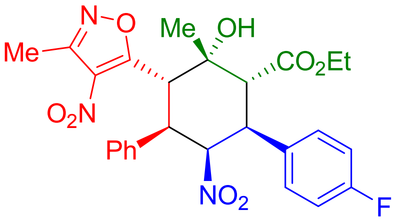

4b

Compound **4b** synthesized with catalyst **I** and isolated as a colorless solid (168 mg, 64%); Mp = 196-198°C; $[\alpha]_D^{24}$ = +60.0 (c = 0.2, $CHCl_3$); >20:1 dr; 99% ee (major diastereomer); HPLC (major diastereomer): t_R 5.59 min (major), 12.60 min (minor), 230 nm, *n*-heptane/*i*-PrOH, 9:1, 0.70 mL/min, Chiralpak IC column; IR (capillary): 3791, 3493, 2981, 2673, 2325, 2097, 1999, 1901, 1720, 1599, 1517, 1365, 1231, 1168, 1023, 832, 758, 701 cm^{-1}; ^1H NMR (400 MHz, $CDCl_3$, major diastereomer): δ = 7.26-7.17 (m, 5H, ArH), 7.11-7.09 (m, 2H, ArH), 7.03-6.99 (m, 2H, ArH), 5.42 (d, *J* = 12.8 Hz, 1H, CH), 5.00 (t, *J* = 4.0 Hz, 1H, CH), 4.44 (dd, *J* = 12.8, 4.5 Hz, 1H, CH), 4.08-4.07 (m, 2H, 2CH), 3.99-3.95 (m, 2H, CH_2), 3.85 (s, 1H, OH), 2.42 (s, 3H, CH_3), 1.33 (s, 3H, CH_3), 0.96 (t, *J* = 7.1 Hz, 3H, CH_3); ^{13}C NMR (101 MHz, $CDCl_3$, major diastereomer): δ = 174.0, 171.5, 162.9, 151.1, 135.5, 131.2, 130.1, 129.5, 128.8, 127.2, 116.1, 93.1, 71.9, 61.7, 50.2, 44.1, 43.6, 43.0, 26.8, 13.8, 11.8 ppm; ^{19}F NMR (376 MHz, $CDCl_3$, major diastereomer): δ = -112.8; MS (EI): *m/z* 527.9 $[M+1]^+$; HRMS Calcd for $[C_{26}H_{26}N_3O_8F+H]^+$: 528.1777, found: 528.1786.

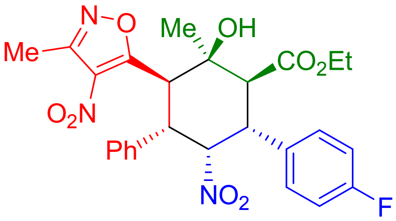

ent-**4b**

Compound *ent*-**4b** was synthesized with catalyst **II** and isolated as a colorless solid (165 mg, 63%); >20:1 dr; 97% ee (major diastereomer); HPLC (major diastereomer): t_R 5.44 min (minor), 11.76 min (major), 230 nm, *n*-heptane/*i*-PrOH, 9:1, 0.70 mL/min, Chiralpak IC column.

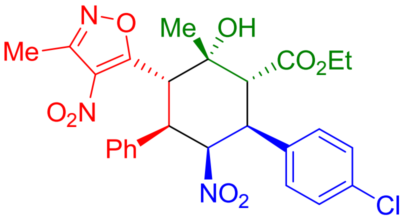

4c

Compound **4c** was synthesized with catalyst **I** and isolated as a colorless solid (149 mg, 55%); Mp = 198-200°C; $[\alpha]_D^{24}$ = +80.0 (c = 0.5, $CHCl_3$); >20:1 dr; 99% ee (major diastereomer); HPLC (major diastereomer): t_R 5.52 min (major), 12.61 min (minor), 230 nm, *n*-heptane/*i*-PrOH, 9:1, 0.70 mL/min, Chiralpak-IC column; IR (capillary): 3786, 3529, 2929, 2670, 2318, 2105, 1992, 1913, 1728, 1600, 1542, 1366, 1252, 1153, 1093, 1020, 890, 828, 747, 702 cm^{-1}; ^1H NMR (600 MHz, $CDCl_3$, major diastereomer): δ = 7.29-7.27 (m, 2H, ArH), 7.19-7.16 (m, 5H, ArH), 7.10-7.19 (m, 2H, ArH), 5.41 (d, *J* = 12.8 Hz, 1H, CH), 5.00 (t, *J* = 3.9 Hz, 1H, CH), 4.43 (dd, *J* = 12.8, 4.6 Hz, 1H), 4.07-4.06 (d, *J* = 5.4 Hz, 2H, 2CH), 4.00-3.96 (m, 2H, CH_2), 3.81 (br s, 1H, OH), 2.41 (s, 3H, CH_3), 1.32 (s, 3H, CH_3), 0.97 (t, *J* = 7.1 Hz, 3H, CH_3); ^{13}C NMR (151 MHz, $CDCl_3$, major diastereomer): δ = 173.9, 171.5, 155.1, 135.4, 134.8, 133.9, 132.5, 129.6, 129.5, 129.4, 128.8, 127.2, 92.9, 71.9, 61.8, 49.9, 44.0, 43.7, 43.0, 26.8, 13.9, 11.8 ppm; MS (EI): *m/z* 543.1$[M]^+$; HRMS Calcd for $[C_{26}H_{26}N_3O_8Cl+H]^+$: 544.1481, found: 544.1482.

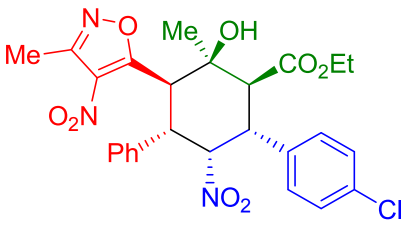

ent-**4c**

Compound *ent*-**4c** was synthesized with catalyst **II** and isolated as a colorless solid (139 mg, 51%); >20:1 dr; 95% ee (major diastereomer); HPLC (major diastereomer): t_R 5.51 min (minor), 12.72 min (major), 230 nm, *n*-heptane/*i*-PrOH, 9:1, 0.70 mL/min, Chiralpak-IC column.

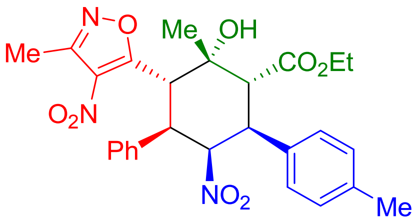

4d

Compound **4d** synthesized with catalyst **I** and isolated as a colorless solid (165 mg, 63%); Mp = 212-213°C; $[\alpha]_D^{24}$ = +54.4 (c = 0.5, $CHCl_3$); >20:1 dr; 93% ee (major diastereomer); HPLC (major diastereomer): t_R 20.24 min (major), 31.66 min (minor), 254nm, *n*-heptane/*i*-

PrOH, 7:3, 0.70 mL/min, Chiralpak-AD column; IR (capillary): 3788, 3488, 2980, 2669, 2323, 2105, 2000, 1905, 1722, 1597, 1530, 1443, 1365, 1259, 1178, 1023, 892, 827, 747, 702 cm^{-1}; ^{1}H NMR (600 MHz, $CDCl_3$, major diastereomer): δ = 7.18-7.16 (m, 3H, ArH), 7.12-7.09 (m, 6H, ArH), 5.42 (d, *J* = 12.6 Hz, 1H, CH), 5.01 (t, *J* = 4.5 Hz, 1H, CH), 4.43 (dd, *J* = 12.6, 4.8 Hz, 1H, CH), 4.10-4.02 (m, 2H, 2CH), 3.99-3.92 (m, 2H, CH_2), 3.90 (s, 1H, OH), 2.42 (s, 3H, CH_3), 2.29 (s, 3H, CH_3), 1.32 (s, 3H, CH_3), 0.94 (t, *J* = 7.2 Hz, 3H, CH_3); ^{13}C NMR (151 MHz, $CDCl_3$, major diastereomer): δ = 174.2, 171.7, 155.0, 138.5, 135.7, 132.5, 132.2, 129.7, 129.4, 129.3, 128.64, 128.1, 127.2, 93.3, 71.9, 61.6, 50.0, 44.0, 43.9, 43.1, 26.9, 21.2, 13.8, 11.8 ppm; MS (EI): *m/z* 523.0 $[M]^+$; HRMS Calcd for $[C_{27}H_{29}N_3O_8+Na]^+$: 546.1847, found: 546.1846.

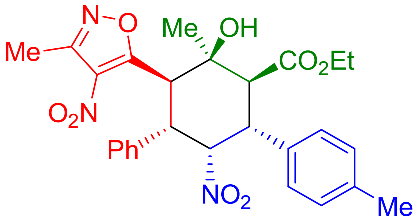

ent-**4d**

Compound *ent*-**4d** was synthesized with catalyst **II** and isolated as a colorless solid (168 mg, 64%); >20:1 dr; 98% ee (major diastereomer); HPLC (major diastereomer): t_R 31.55 min (minor), 20.31 min (major), 254 nm, *n*-heptane/*i*-PrOH, 7:3, 0.70 mL/min, Chiralpak-AD column.

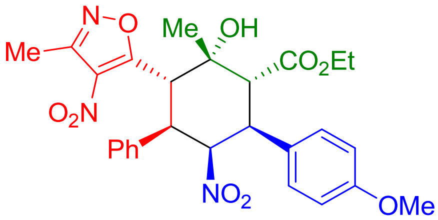

4e

Compound **4e** was synthesized with catalyst **I** and isolated as a colorless solid (180 mg, 67%); Mp = 109-110°C; $[\alpha]_D^{24}$ = +64.4 (c = 0.5, $CHCl_3$); >20:1 dr; 97% ee (major diastereomer); HPLC (major diastereomer): t_R 36.31 min (major), 54.49 min (minor), 230 nm, *n*-heptane/ethanol, 9:1, 1.00 mL/min, Chiralpak IA column; IR (capillary): 3790, 3487, 2977, 2670, 2324, 2099, 1999, 1896, 1723, 1598, 1517, 1450, 1365, 1253, 1180, 1027, 893, 830, 756, 702 cm^{-1}; ^{1}H NMR (400 MHz, $CDCl_3$, major diastereomer): δ = 7.18-7.15 (m, 5H, ArH), 7.11-7.10 (m, 2H, ArH), 6.82 (d, *J* = 8.6 Hz, 2H, ArH), 5.41 (d, *J* = 12.7 Hz, 1H, CH),

5.00 (t, J = 4.2 Hz, 1H, CH), 4.42 (dd, J = 12.8, 4.5 Hz, 1H, CH), 4.08-4.03 (m, 2H, 2CH), 3.98-3.95 (m, 2H, CH_2), 3.91 (s, 1H, OH), 3.76 (s, 3H, CH_3), 2.42 (s, 3H, CH_3), 1.32 (s, 3H, CH_3), 0.95 (t, J = 7.1 Hz, 3H, CH_3); ^{13}C NMR (101 MHz, $CDCl_3$, major diastereomer): δ = 174.22, 171.7, 159.8, 155.0, 153.7, 135.7, 132.5, 129.4, 128.6, 127.2, 114.4, 93.3, 71.9, 61.6, 55.3, 50.2, 44.0, 43.6, 43.0, 26.9, 13.9, 11.8 ppm; MS (EI): *m/z* 539.1 $[M]^+$; HRMS Calcd for $[C_{27}H_{29}N_3O_9+Na]^+$: 562.1796, found: 562.1803.

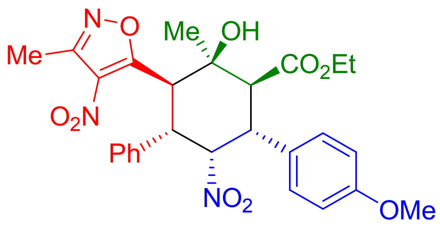

ent-**4e**

Compound *ent*-**4e** was synthesized with catalyst **II** and isolated as a colorless solid (177 mg, 66%); >20:1 dr; 95% ee (major diastereomer); HPLC (major diastereomer): t_R 36.35 min (minor), 54.29 min (major), 230 nm, *n*-heptane/ethanol, 9:1, 1.00 mL/min, Chiralpak IA column.

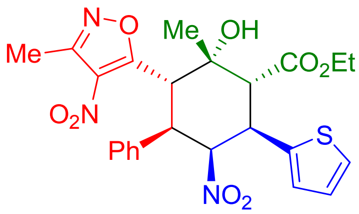

4f

Compound **4f** was synthesized with catalyst **I** and isolated as a colorless solid (158 mg, 61%); Mp = 232-233 °C; $[\alpha]_D^{24}$ = +73.8 (c = 0.5, $CHCl_3$); >20:1 dr; 91% ee (major diastereomer); HPLC (major diastereomer): t_R 6.91 min (major) 13.99 min (minor), 230 nm, *n*-heptane/*i*-PrOH, 9:1, 0.7 mL/min, Chiralpak IC column; IR (capillary): 3532, 3110, 2985, 2651, 2323, 2182, 2068, 1998, 1953, 1899, 1811, 1730, 1598, 1551, 1517, 1444, 1418, 1370, 1344, 1245, 1212, 1179, 1141, 1023, 982, 926, 888, 830, 760, 703 cm^{-1}; ^{1}H NMR (400 MHz, $CDCl_3$, major diastereomer): δ = 7.22-7.09 (m, 6H, ArH), 6.96 -6.92 (m, 2H, ArH), 5.38 (d, J = 12.8 Hz, 1H, CH), 5.09 (t, J = 4.4 Hz, 1H, CH), 4.46-4.34 (m, 2H, 2CH), 4.10-3.98 (m, 3H, CH, CH_2), 3.83 (s, 1H, OH), 2.41 (s, 3H, CH_3), 1.32 (s, 3H, CH_3), 1.01 (t, J = 7.1 Hz, 3H, CH_3); ^{13}C NMR (101 MHz, $CDCl_3$, major diastereomer): δ = 173.8, 171.5, 155.0, 137.2, 135.5, 132.6, 129.5, 128.7, 127.4, 127.2, 126.8, 125.7, 93.2, 71.9, 61.8, 51.6, 43.8, 43.0, 39.4,

26.7, 13.8, 11.8 ppm; MS (EI): *m/z* 516.1 $[M+H]^+$; HRMS Calcd for $[C_{24}H_{25}N_3O_8S +Na]^+$: 538.1255, found: 538.1251.

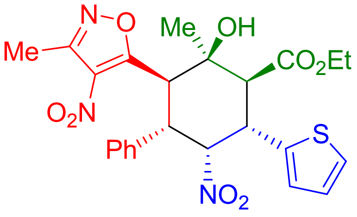

ent-**4f**

Compound *ent*-**4f** was synthesized with catalyst **II** and isolated as a colorless solid (152 mg, 59%); >20:1 dr; 96% ee (major diastereomer); HPLC (major diastereomer): t_R 6.94 min (minor), 13,94 min (major), 230 nm, *n*-heptane/*i*-PrOH, 9:1, 0.7 mL/min, Chiralpak IC column.

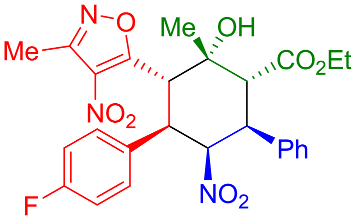

4g

Compound **4g** was synthesized with catalyst **I** and isolated as a colorless solid (160 mg, 60%); Mp = 201-203°C; $[\alpha]_D^{24}$ = +52.0 (c = 0.5, $CHCl_3$); >20:1 dr; 98% ee (major diastereomer); HPLC (major diastereomer): t_R 14.06 min (major), 17.91 min (minor), 214 nm, *n*-heptane/*i*-PrOH, 7:3, 1.00 mL/min, Chiralcel AD column; IR (capillary): 3790, 3491, 2982, 2671, 2322, 2087, 1899, 1719, 1597, 1524, 1364, 1172, 1024, 831, 761, 701 cm^{-1}; ^1H NMR (400 MHz, $CDCl_3$, major diastereomer): δ = 7.31-7.29 (m, 3H, ArH), 7.24-7.23 (m, 2H, ArH), 7.12-7.09 (m, 2H, ArH), 6.90-6.86 (m, 2H, ArH), 5.38 (dd, *J* = 12.8, 1.4 Hz, 1H, CH), 5.01-4.99 (m, 1H, CH), 4.44 (dd, *J* = 12.8, 4.5 Hz, 1H, CH), 4.08-4.07 (m, 2H, 2CH), 3.97-3.91 (m, 3H, OH, CH_2); 2.44 (s, 3H, CH_3), 1.32 (s, 3H, CH_3), 0.90 (t, *J* = 7.1 Hz, 3H, CH_3); ^{13}C NMR (151 MHz, $CDCl_3$, major diastereomer): δ = 174.1, 171.4, 162.6, 155.1, 135.1, 132.6, 131.5, 129.3, 128.9, 128.3, 127.8, 116.5, 93.0, 71.9, 61.7, 49.9, 44.3, 43.3 (2C), 26.8, 13.7, 11.8 ppm; ^{19}F NMR (376 MHz, $CDCl_3$. major diastereomer): δ = -112.6; MS (EI): *m/z* 528.0 $[M+1]^+$; HRMS Calcd for $[C_{26}H_{26}N_3O_8F+Na]^+$: 550.1596, found: 550.1597.

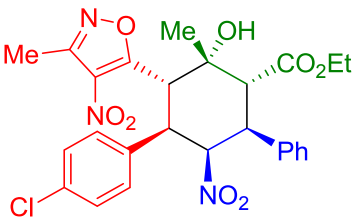

4h

Compound **4h** was synthesized with catalyst **I** and isolated as a colorless solid (167 mg, 61%); Mp = 192-193 °C; $[\alpha]_D^{24}$ = +57.6 (c = 0.5, $CHCl_3$); >20:1 dr; 97% ee (major diastereomer); HPLC (major diastereomer): t_R 14.27 min (major), 18.99 min (minor), 230 nm, *n*-heptane/i-PrOH, 7:3, 1.00 mL/min, Chiralpak AD column; IR (capillary): 3498, 3066, 3034, 2981, 2938, 26.53, 2507, 2324, 2226, 2161, 2069, 1989, 1954, 1903, 1706, 1597, 1550, 1522, 1451, 1414, 1368, 1261, 1187, 1094, 1015, 893, 827, 795, 755, 697 cm^{-1}; ^1H NMR (600 MHz, $CDCl_3$, major diastereomer): δ = 7.32-7.27 (m, 3H, ArH), 7.24-7.22 (m, 2H, ArH), 7.17-7.16 (m, 2H, ArH), 7.07-7.05 (m, 2H, ArH), 5.38 (dd, *J* = 12.8, 1.3 Hz, 1H, CH), 4.98 (t, *J* = 3.9 Hz, 1H, CH), 4.44 (dd, *J* = 12.8, 4.5 Hz, 1H), 4.07-4.06 (m, 2H, 2CH), 3.98-3.90 (m, 3H, CH_2, OH), 2.46 (s, 3H, CH_3), 1.32 (s, 3H, CH_3), 0.90 (t, *J* = 7.1 Hz, 3H, CH_3); ^{13}C NMR (151 MHz, $CDCl_3$, major diastereomer): δ = 174.1, 171.3, 155.2, 135.1, 134.6, 134.2, 132.6, 129.7, 129.1, 128.9, 128.7, 128.3, 127.8, 92.9, 71.9, 61.7, 49.9, 44.3, 43.4, 43.1, 26.8, 13.7, 11.9 ppm; MS (EI): *m/z* 544.1 $[M+1]^+$; Anal. Calcd for $C_{26}H_{26}N_3O_8Cl$: C, 57.41; H, 4.82; N, 7.72, found: C, 57.11; H, 4.88; N 7.45.

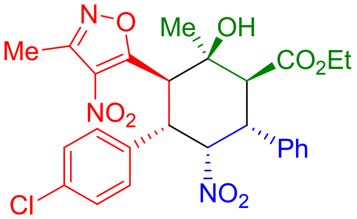

ent-**4h**

Compound *ent*-**4h** was synthesized with catalyst **II** and isolated as a colorless solid (164 mg, 60%); 20:1 dr; 97% ee (major diastereomer); HPLC (major diastereomer): t_R 14.28 min (minor), 19.05 min (major), 230 nm, *n*-heptane/*i*-PrOH, 7:3, 1.00 mL/min, Chiralpak AD column.

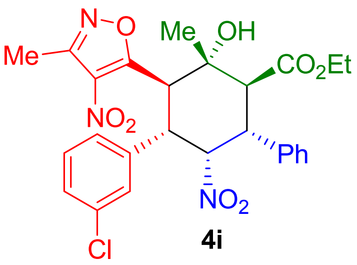

4i

Compound **4i** was synthesized with catalyst **I** and isolated as a colorless solid (189 mg, 69%); Mp = 216-218 °C; $[\alpha]_D^{24}$ = +68.0 (c = 0.2, $CHCl_3$); >20:1 dr; 97% ee (major diastereomer); HPLC (major diastereomer): t_R 10.16 min (major), 19.54 min (minor), 230 nm, *n*-heptane/EtOH, 9:1, 1.00 mL/min, Chiralcel OD column; IR (capillary): 3578, 2978, 2679, 2326, 2100, 1996, 1897, 1718, 1596, 1543, 1364, 1255, 1028, 898, 829, 760, 697 cm^{-1}; ^1H NMR (600 MHz, $CDCl_3$, major diastereomer): δ = 7.33-7.27 (m, 3H, ArH), 7.24-7.22 (m, 2H, ArH), 7.17-7.14 (m, 2H, ArH), 7.12-7.10 (m, 1H, ArH), 6.97 (d, *J* = 7.7 Hz, 1H, ArH), 5.40 (dd, *J* = 12.8, 1.6 Hz, 1H, CH), 5.03-5.02 (m, 1H, CH), 4.44 (dd, *J* = 12.8, 4.5 Hz, 1H, CH), 4.08-4.07 (m, 2H, 2CH), 3.99-3.90 (m, 3H, CH_2, OH), 2.45 (s, 3H, CH_3), 1.32 (s, 3H, CH_3), 0.90 (t, *J* = 7.1 Hz, 3H, CH_3); ^{13}C NMR (151 MHz, $CDCl_3$, major diastereomer): δ = 174.1, 171.2, 155.2, 137.6, 135.2, 135.0, 132.6, 130.7, 129.2, 129.1, 128.9, 128.3, 127.8, 124.7, 92.8, 71.9, 61.7, 49.9, 44.3, 43.8, 42.9, 26.8, 13.7, 11.8 ppm; MS (EI): *m/z* 543.0 $[M]^+$; HRMS Calcd for $[C_{26}H_{26}N_3O_8Cl+Na]^+$: 566.1301, found: 566.1301.

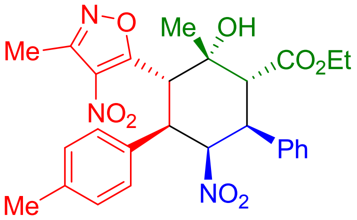

4j

Compound **4j** was synthesized with catalyst **I** and isolated as a colorless solid (190 mg, 73%); Mp = 186-188 °C; $[\alpha]_D^{24}$ = +57.6 (c = 0.5, $CHCl_3$); >20:1 dr; 98% ee (major diastereomer); HPLC (major diastereomer): t_R 10.22 min (major), 18.64 min (minor), 254 nm, *n*-heptane/EtOH, 9:1, 1.00 mL/min, Chiralcel OD column; IR (capillary): 3506, 2980, 2933, 2735, 2651, 2509, 2323, 2230, 2176, 2112, 2067, 2014, 1983, 1951, 1808, 1712, 1597, 1551, 1519, 1452, 1417, 1370, 1260, 1183, 1111, 1023, 978, 895, 827, 792, 754, 700 cm^{-1}; ^1H NMR (600 MHz, $CDCl_3$, major diastereomer): δ = 7.31-7.23 (m, 6H, ArH), 6.99-6.95 (m, 3H, ArH), 5.41 (dd, *J* = 12.8, 1.4 Hz, 1H, CH), 5.01 (t, *J* = 4.3 Hz, 1H, CH), 4.40 (dd, *J* = 12.8, 4.6 Hz, 1H, CH), 4.11-4.07 (m, 2H, 2CH), 3.98-3.89 (m, 3H, CH_2, OH), 2.43 (s, 3H,

CH_3), 2.20 (s, 3H, CH_3), 1.33 (s, 3H, CH_3), 0.90 (t, J = 7.1 Hz ,3H, CH_3); ^{13}C NMR (151 MHz, $CDCl_3$, major diastereomer): δ = 174.2, 171.8, 155.0, 138.4, 135.3, 132.5, 130.1, 130.0, 129.1, 128.8, 128.3, 127.8, 127.0, 93.2, 71.9, 61.6, 49.9, 44.3, 43.6, 43.1, 26.9, 21.2, 13.7, 11.9 ppm; MS (EI): *m/z* 523.1 $[M]^+$; Anal. Calcd for $C_{27}H_{29}N_3O_8$: C, 61.94; H, 5.58; N, 8.03, found: C, 61.70; H, 5.94; N 7.65.

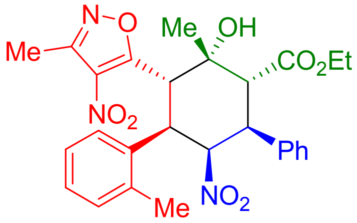

4k

Compound **4k** was synthesized with catalyst **I** and isolated as a colorless solid (128 mg, 49%); Mp = 180-182 °C; $[\alpha]_D^{24}$ = +45.0 (c = 0.2, $CHCl_3$); >20:1 dr; 95% ee (major diastereomer); HPLC (major diastereomer): t_R 5.14 min (major), 13.93 min (minor), 230 nm, *n*-heptane/*i*-PrOH, 9:1, 0.70 mL/min, Chiralpak IC column; IR (capillary): 3788, 2523, 2979, 2669, 2323, 2083, 1989, 1902, 1723, 1598, 1545, 1444, 1364, 1253, 1164, 1026, 890, 829, 752, 699 cm^{-1}; ^{1}H NMR (600 MHz, $CDCl_3$, major diastereomer): δ = 7.32-7.28 (m, 3H, ArH), 7.25-7.24 (m, 2H, ArH), 7.08-7.02 (m, 2H, ArH), 6.97-6.91 (m, 2H, ArH), 5.43 (dd, J = 12.7, 1.6 Hz, 1H, CH), 4.96-4.95 (m, 1H, CH), 4.72 (dd, J = 12.7, 4.5 Hz, 1H, CH), 4.15 (d, J = 12.8 Hz, 1H, CH), 4.05 (dd, J = 12.8, 4.4 Hz, 1H, CH), 3.99-3.91 (m, 2H, CH_2), 3.87 (d, J = 1.6 Hz, 1H, OH), 2.45 (s, 3H, CH_3), 2.41 (s, 3H, CH_3), 1.34 (s, 3H, CH_3), 0.91 (t, J = 7.1 Hz, 3H, CH_3); ^{13}C NMR (151 MHz, $CDCl_3$, major diastereomer): δ = 174.2, 171.9, 155.1, 136.1, 135.3, 133.5, 132.5, 131.3, 129.6, 129.1, 128.8, 128.4, 128.3, 128.0, 127.1, 125.0, 91.1, 72.0, 61.6, 50.1, 44.6, 43.4, 39.1, 27.0, 19.7, 13.8, 11.8 ppm; MS (EI): *m/z* 523.0 $[M]^+$; HRMS Calcd for $[C_{27}H_{29}N_3O_8+Na]^+$: 546.1847, found: 546.1846.

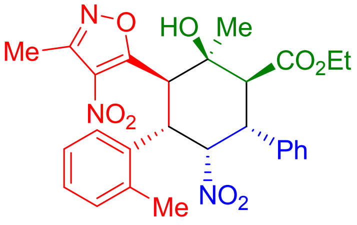

ent-**4k**

Compound *ent*-**4k** was synthesized with catalyst **II** and isolated as a colorless solid (132 mg, 50%); >20:1 dr; 96% ee (major diastereomer); HPLC (major diastereomer): t_R 5.14 min

(minor), 13.63 min (minor), 230 nm, *n*-heptane/*i*-PrOH, 9:1, 0.70 mL/min, Chiralpak IC column.

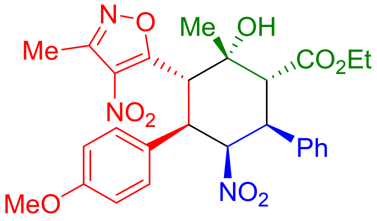

4l

Compound **4l** was synthesized with catalyst **I** and isolated as a colorless solid (105 mg, 39%); Mp = 110-112 °C; $[\alpha]_D^{24}$ = +62.8 (c = 0.5, $CHCl_3$); >20:1 dr; 96% ee (major diastereomer); HPLC (major diastereomer): t_R 15.96 min (major), 23.00 min (minor), 214 nm, *n*-heptane/*i*-PrOH, 7:3, 1.00 mL/min, Chiralpak AD column; IR (capillary): 3786, 3474, 2971, 2682, 2322, 2115, 1999, 1927, 1731, 1598, 1523, 1445, 1365, 1248, 1186, 1024, 894, 828, 759, 700 cm^{-1}; ^1H NMR (600 MHz, $CDCl_3$, major diastereomer): δ = 7.31-7.22 (m, 5H, ArH), 7.03 (d, *J* = 8.7 Hz, 2H, ArH), 6.69 (d, *J* = 8.9 Hz, 2H, ArH), 5.38 (dd, *J* = 12.8, 1.6 Hz, 1H, CH), 5.00 (t, *J* = 4.3 Hz, 1H, CH), 4.38 (dd, *J* = 12.8, 4.6 Hz, 1H, CH), 4.09-4.07 (m, 2H, 2CH), 3.96-3.89 (m, 3H, CH_2, OH), 3.69 (s, 3H, CH_3), 2.43 (s, 3H, CH_3), 1.32 (s, 3H, CH_3), 0.90 (t, *J* = 7.1 Hz, 3H, CH_3); ^{13}C NMR (151 MHz, $CDCl_3$, major diastereomer): δ = 174.2, 171.8, 159.5, 155.1, 135.3, 132.5, 129.2, 129.1, 128.8, 128.4, 128.3, 127.9, 127.8, 127.5, 114.7, 93.3, 71.9, 61.6, 55.2, 49.9, 44.3, 43.3, 43.2, 26.9, 13.7, 11.9 ppm; MS (EI): *m/z* 539.0 $[M]^+$; HRMS Calcd for $[C_{27}H_{29}N_3O_9+Na]^+$: 562.1796, found: 562.1796.

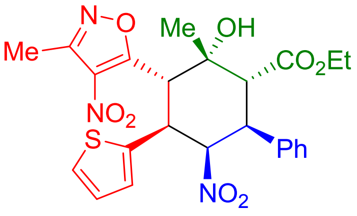

4m

Compound **4m** was synthesized with catalyst **I** and isolated as a colorless solid (128 mg, 50%); Mp = 230-232°C; $[\alpha]_D^{24}$ = +79.8 (c = 0.5, $CHCl_3$); >20:1 dr; 97% ee (major diastereomer); HPLC (major diastereomer): t_R 7.47 min (major), 16.39 min (minor), 214 nm, *n*-heptane/*i*-PrOH, 9:1, 0.70 mL/min, Chiralpak IC column; IR (capillary): 3787, 3483, 2981, 2666, 2318, 2080, 1987, 1721, 1599, 1537, 1364, 1257, 1196, 1024, 832, 704 cm^{-1}; ^1H NMR (600 MHz, $CDCl_3$, major diastereomer): δ = 7.33-7.29 (m, 3H, ArH), 7.24-7.22 (m, 2H, ArH), 7.08 (dd, *J* = 4.9, 0.9 Hz, 1H, ArH), 6.79-6.76 (m, 2H, ArH), 5.40 (dd, *J* = 12.6, 1.6

Hz, 1H, CH), 5.10-5.09 (m, 1H, CH), 4.71 (dd, *J* = 12.6, 4.6 Hz, 1H, CH), 4.06-4.05 (m, 2H, 2CH), 4.98-3.90 (m, 3H, CH_2, OH), 2.46 (s, 3H, CH_3), 1.32 (s, 3H, CH_3), 0.90 (t, *J* = 7.1 Hz, 3H, CH_3); ^{13}C NMR (151 MHz, $CDCl_3$, major diastereomer): δ = 174.1, 171.4, 155.2, 137.6, 135.1, 132.7, 131.0, 129.1, 128.9, 128.2, 127.5, 125.9, 125.8, 93.2, 71.9, 61.6, 49.8, 44.8, 44.1, 39.3, 26.8, 13.7, 11.9; MS (EI): *m/z* 516.0 $[M+1]^+$; HRMS Calcd for $[C_{24}H_{25}N_3O_8S+Na]^+$: 538.1255, found: 538.1255.

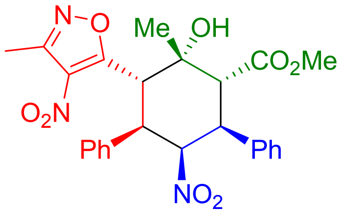

4n

Compound **4n** was synthesized with catalyst **I** and isolated as a colorless solid (145 mg, 58%); Mp = 236-238 °C; $[\alpha]_D^{24}$ = +79.8 (c = 0.5, $CHCl_3$); >20:1 dr; 97% ee (major diastereomer); HPLC (major diastereomer): t_R 5.58 min (major), 8.13 min (minor), 230 nm, *n*-heptane/EtOH, 9:1, 0.70 mL/min, Chiralpak-IC column; IR (capillary): 3782, 3704, 3514, 3254, 2953, 2648, 2514, 2326, 2226, 2160, 2101, 2066, 1990, 1950, 1905, 1710, 1597, 1551, 1518, 1433, 1357, 1262, 1202, 1163, 1074, 1009, 957, 919, 890, 861, 827, 789, 750, 700 cm^{-1}; ^1H NMR (400 MHz, $CDCl_3$, major diastereomer): δ = 7.32-7.27 (m, 3H, ArH), 7.25-7.23 (m, 2H, ArH), 7.19-7.16 (m, 3H, ArH), 7.13-7.11 (m, 2H, ArH), 5.45 (d, *J* = 12.8 Hz, 1H, CH), 5.05 (t, *J* = 4.3 Hz, 1H, CH), 4.47 (dd, *J* = 12.8, 4.5 Hz, 1H), 4.19-4.10 (m, 2H, 2CH), 3.74 (br s, 1H), 3.49 (s, 3H, CH_3), 2.43 (s, 3H, CH_3), 1.34 (s, 3H, CH_3); ^{13}C NMR (101 MHz, $CDCl_3$, major diastereomer): δ = 174.5, 171.6, 155.1, 135.5, 135.3, 132.5, 129.4, 129.2, 128.9, 128.8, 128.7, 128.0, 127.2, 93.1, 71.9, 52.3, 50.0, 44.2, 44.0, 43.1, 26.9, 11.8 ppm; MS (EI): *m/z* 495.9 $[M]^+$; Anal. Calcd for $C_{25}H_{25}N_3O_8$: C, 60.60; H, 5.09; N, 8.48, found: C, 60.60; H, 5.37; N 8.12.

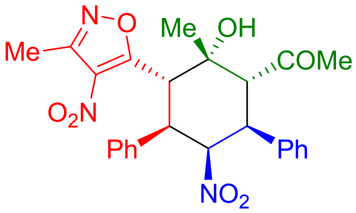

4o

Compound **4a** was synthesized with catalyst **I** and isolated as a colorless solid (120 mg, 50%); Mp = 244-245 °C; $[\alpha]_D^{24}$ = +68.0 (c = 0.5, $CHCl_3$); >20:1 dr; 96% ee (major diastereomer); HPLC (major diastereomer): t_R 12.77 min (major), 25.18 min (minor), 214

nm, *n*-heptane/EtOH, 97:3, 0.70 mL/min, Chiralpak IC column; IR (capillary): 3575, 3484, 2981, 2920, 2321, 2153, 2065, 1992, 1954, 1898, 1700, 1596, 1549, 1520, 1452, 1417, 1360, 1255, 1204, 1141, 1076, 1032, 996, 953, 894, 854, 828, 759, 700 cm^{-1}; ^{1}H NMR (400 MHz, $CDCl_3$, major diastereomer): δ = 7.33-7.29 (m, 3H, ArH), 7.23-7.16 (m, 5H, ArH), 7.12-7.09 (m, 2H, ArH), 5.46 (d, *J* = 12.8 Hz, 1H, CH), 5.00 (t, *J* = 4.5 Hz, 1H, CH), 4.44 (dd, *J* = 12.8, 4.5 Hz, 1H, CH), 4.38 (d, *J* = 12.6 Hz, 1H, CH), 4.03-4.00 (m, 2H, CH, OH), 2.42 (s, 3H, CH_3), 2.09 (s, 3H, CH_3), 1.31 (s, 3H, CH_3); ^{13}C NMR (101 MHz, $CDCl_3$, major diastereomer): δ = 214.5, 171.7, 155.0, 135.6, 135.5, 132.7, 129.6, 129.4, 129.1, 128.7, 128.1, 127.2, 93.2, 72.6, 54.40, 44.7, 44.1, 43.4, 35.2, 26.9, 11.8 ppm; *m/z* 480 $[M+1]^+$; HRMS Calcd for $[C_{25}H_{25}N_3O_7+H]^+$: 480.1765, found: 480.1769.

Procedure for the gram-scale organocatalytic one-pot 1,4-1,6-/1,2-additon reaction:

In a 100 mL round bottom flask equipped with a magnetic stirring bar, the nitroalkene **2a** (1.0 equiv., 8.0 mmol) and catalyst **I** (0.5 mol%) were dissolved in CH_2Cl_2 (10 mL) and stirred 5 minutes at room temperature followed by the addition of ethyl acetoacetate (1.0 equiv. 8.0 mmol). After stirring the reaction mixture at room temperature for 48 hours the 4-nitro-5-styrylisoxazole **3a** (2 equiv., 16 mmol) and DBU (30 mol%; 0.1 M in CH_2Cl_2) were added subsequently and stirred for another 48 hours at room temperature. The crude product was directly purified by flash column chromatography (first *n*-hexane/EtOAc = 9:1, then *n*-hexane/EtOAc = 4:1) to afford 2.34 g (57% yield) of the polysubstituted cyclohexane **4a** .

NMR Spectra:

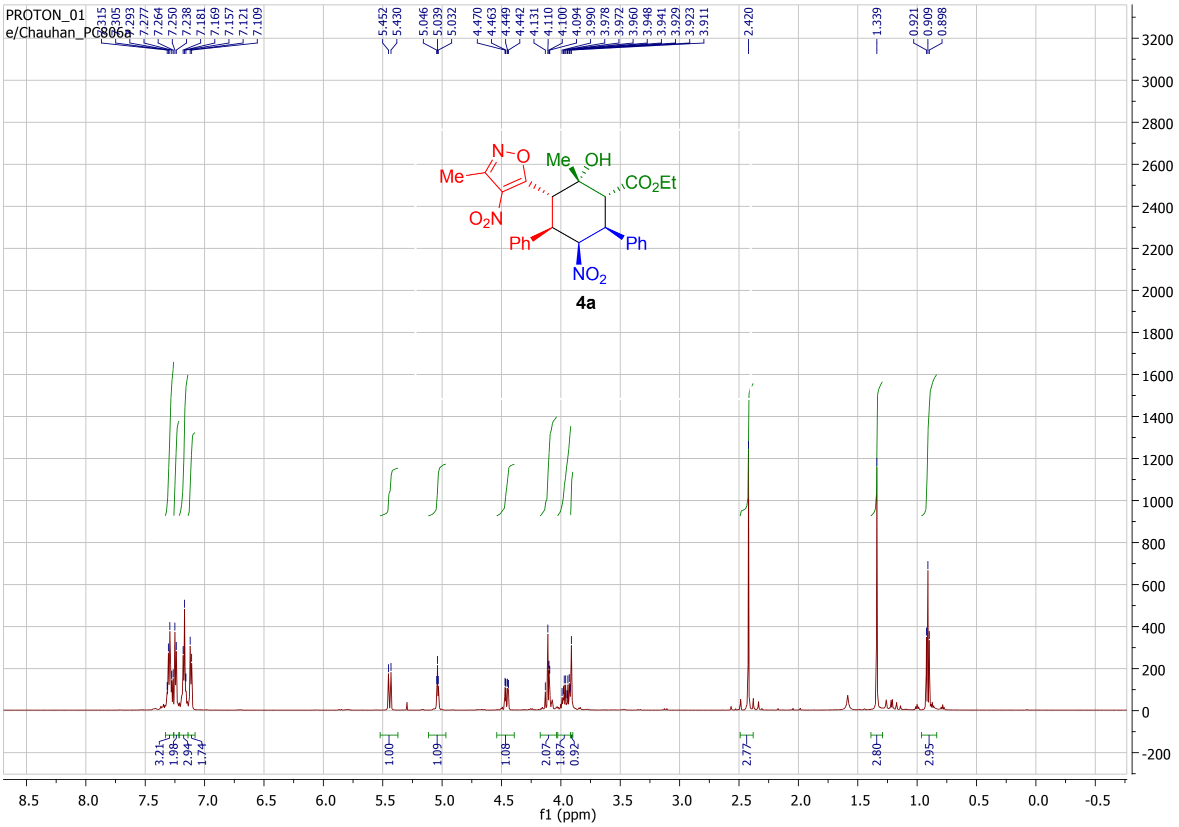
PROTON_01
e/Chauhan_PC806a
4a
f1 (ppm)

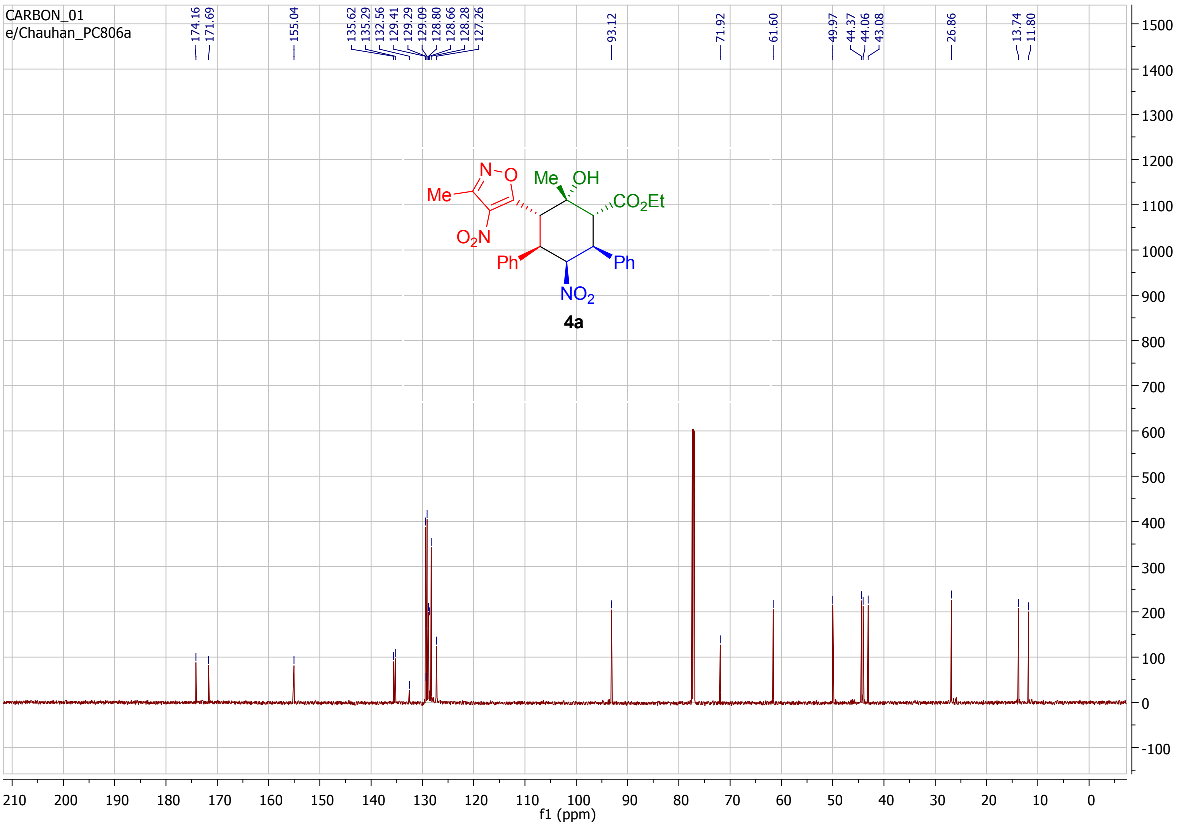
CARBON_01
e/Chauhan_PC806a
4a
f1 (ppm)

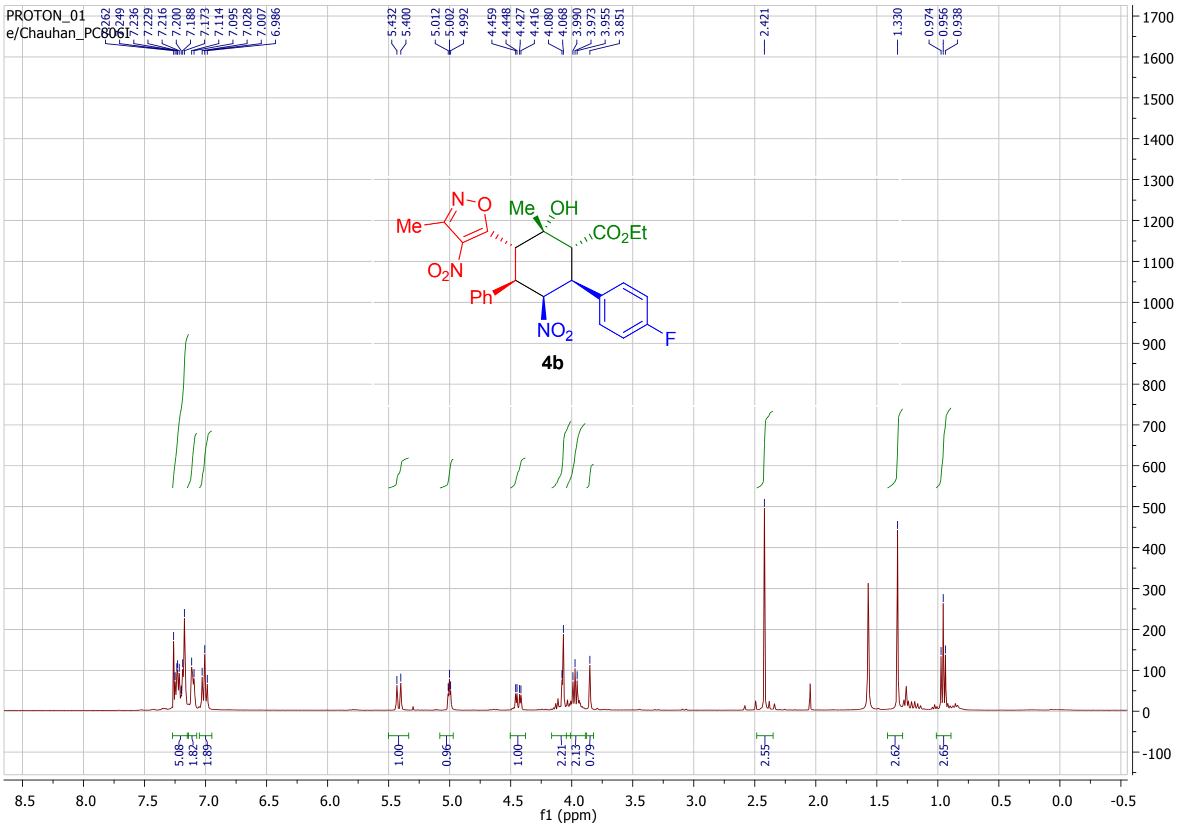
PROTON_01
e/Chauhan_PC806I
7.262
7.249
7.236
7.229
7.216
7.200
7.188
7.173
7.114
7.095
7.028
7.007
6.986
5.432
5.400
5.012
5.002
4.992
4.459
4.448
4.427
4.416
4.080
4.068
3.990
3.973
3.955
3.851
2.421
1.330
0.974
0.956
0.938
Me
OH
CO2Et
N
O
O2N
Ph
NO2
F
4b
5.08
1.82
1.89
1.00
0.96
1.00
2.21
2.13
0.79
2.55
2.62
2.65
f1 (ppm)

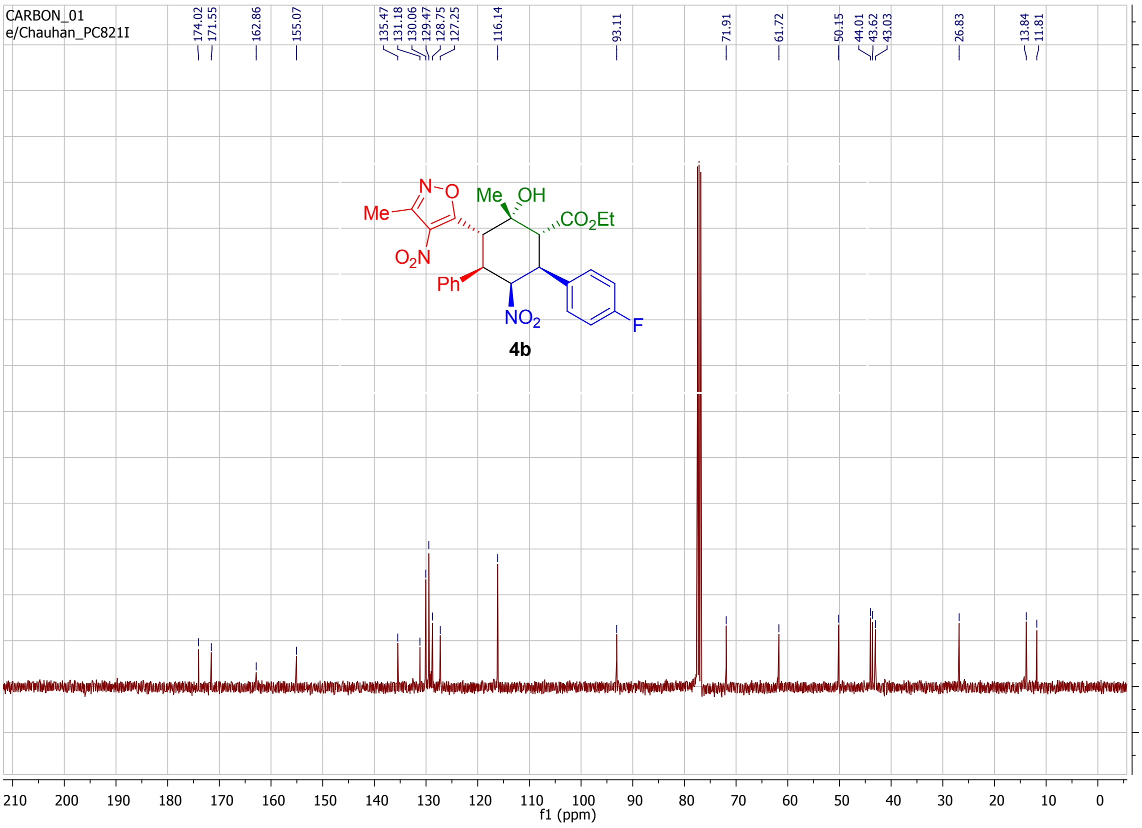
CARBON_01
e/Chauhan_PC821I
174.02
171.55
162.86
155.07
135.47
131.18
130.06
129.47
128.75
127.25
116.14
93.11
71.91
61.72
50.15
44.01
43.62
43.03
26.83
13.84
11.81
Me
OH
CO2Et
N
O
O2N
Ph
NO2
F
4b
f1 (ppm)

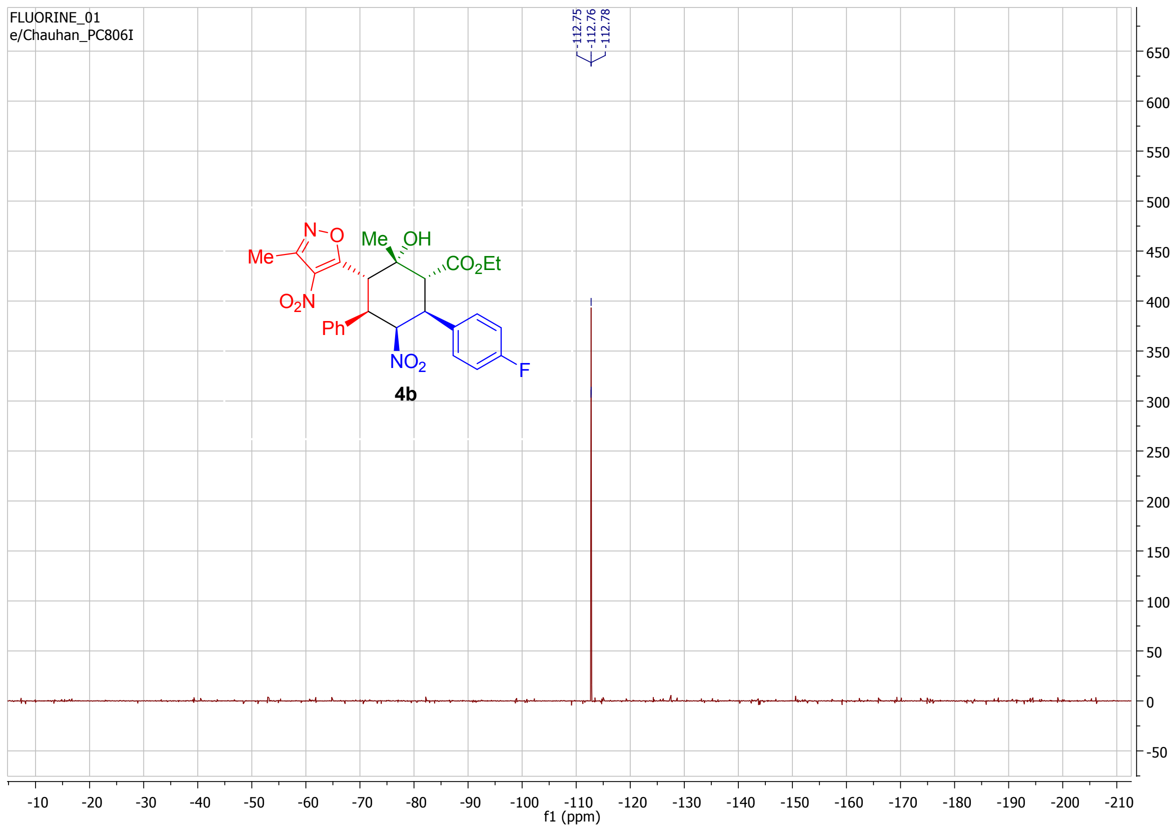

FLUORINE_01
e/Chauhan_PC806I
-112.75
-112.76
-112.78
Me
OH
CO2Et
N
O
Me
O2N
Ph
NO2
F
4b
650
600
550
500
450
400
350
300
250
200
150
100
50
0
-50
-10
-20
-30
-40
-50
-60
-70
-80
-90
-100
-110
-120
-130
-140
-150
-160
-170
-180
-190
-200
-210
f1 (ppm)

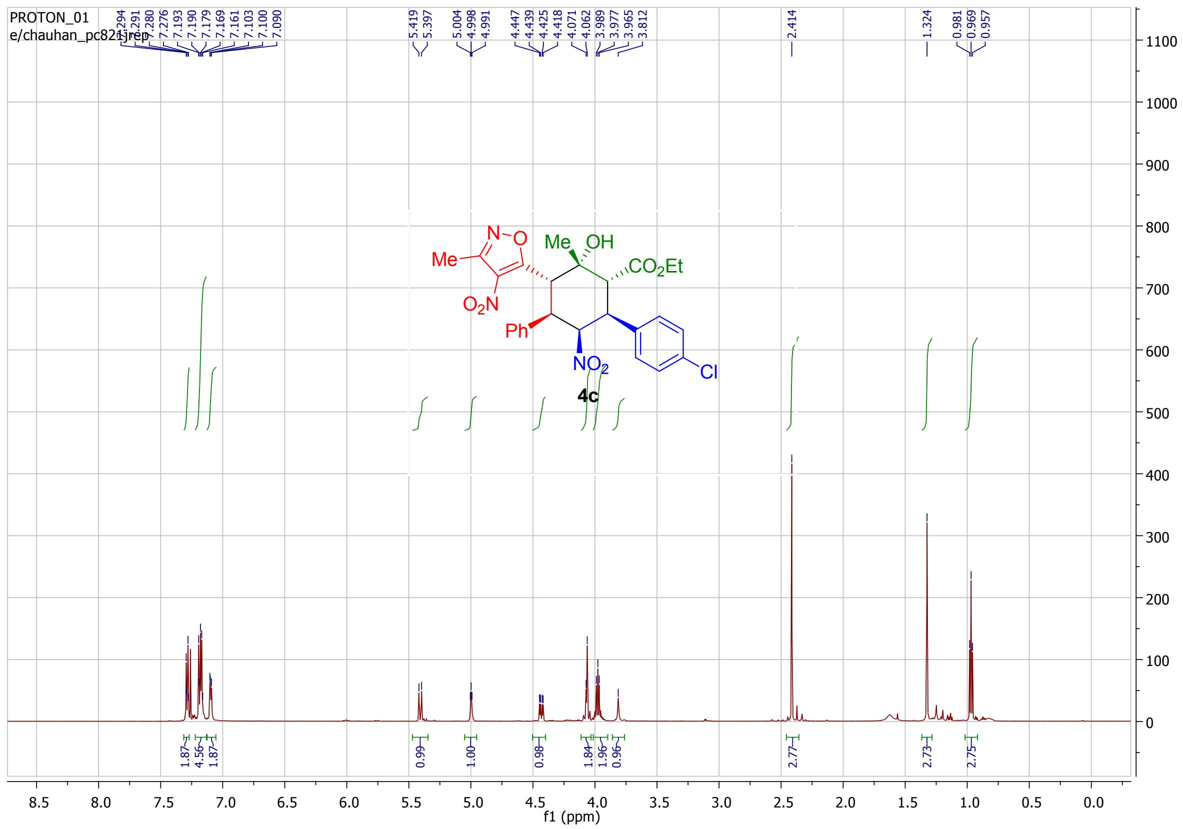
PROTON_01
e/chauhan_pc821jrep
4c
f1 (ppm)

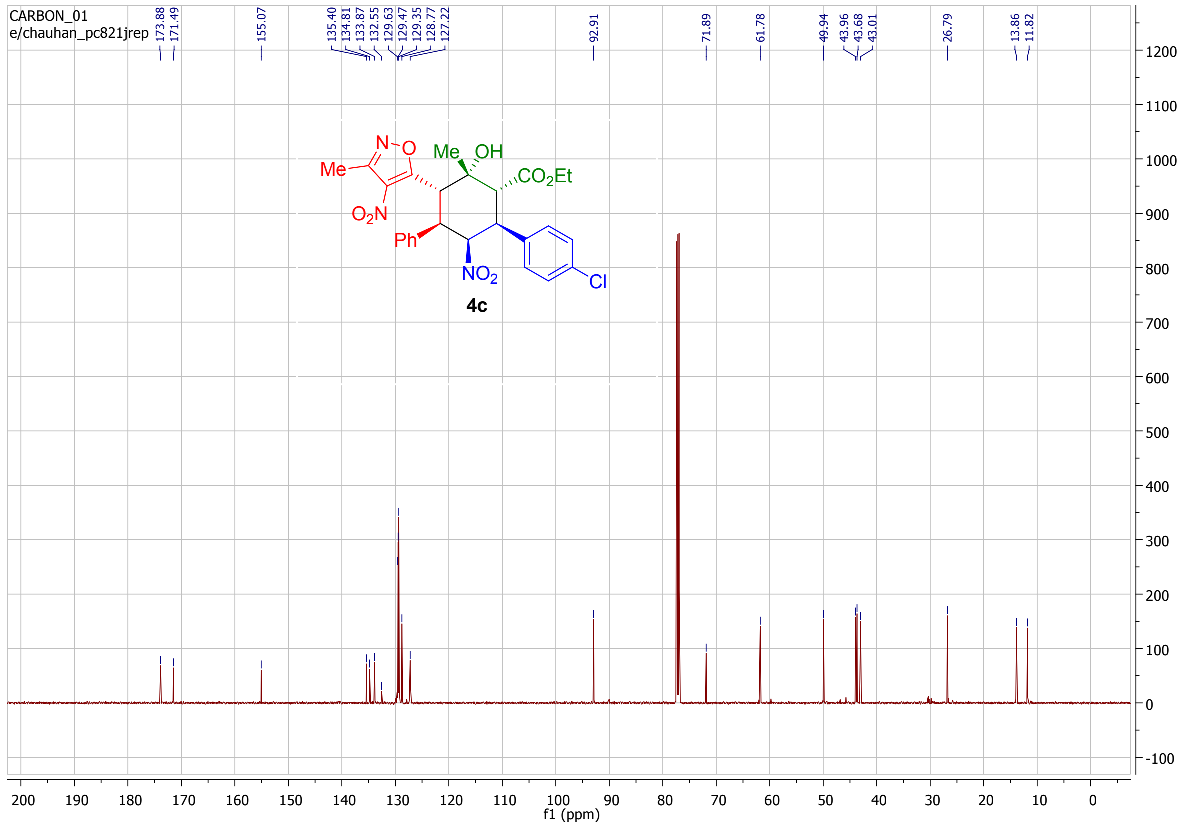
CARBON_01
e/chauhan_pc821jrep
4c
f1 (ppm)

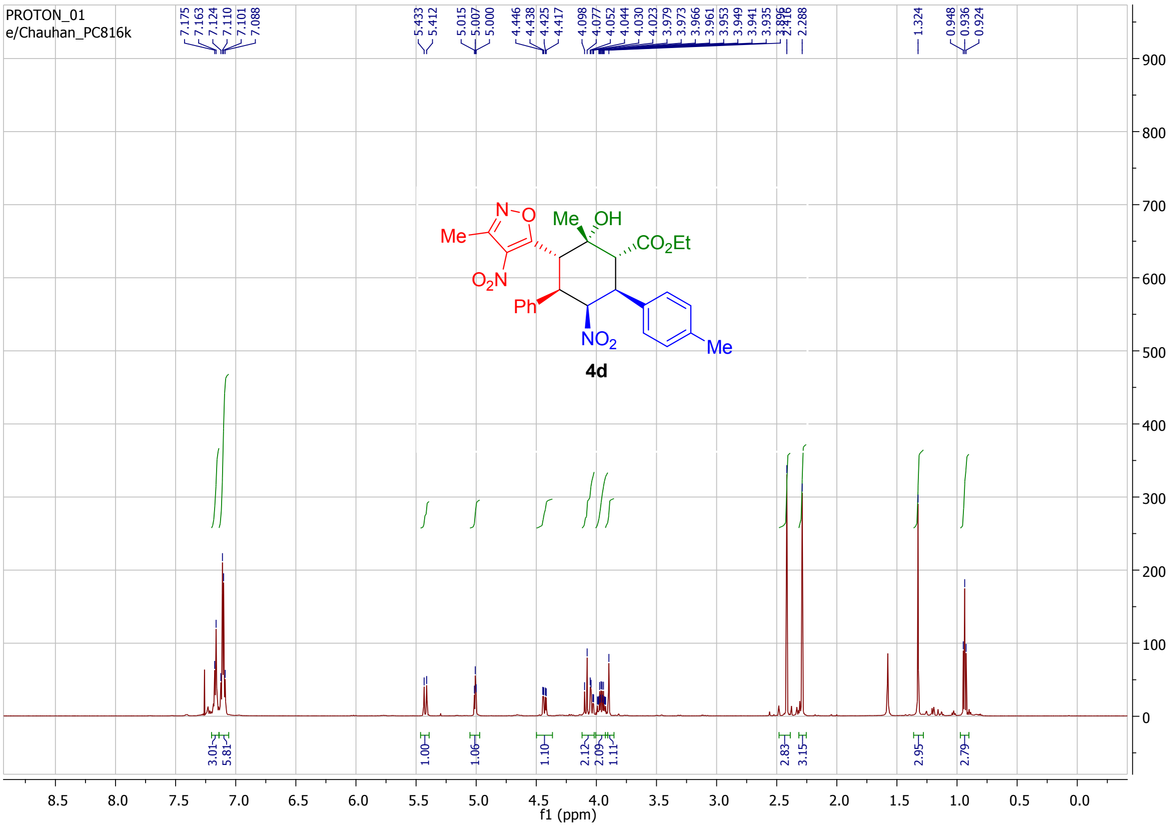
PROTON_01
e/Chauhan_PC816k
f1 (ppm)
4d

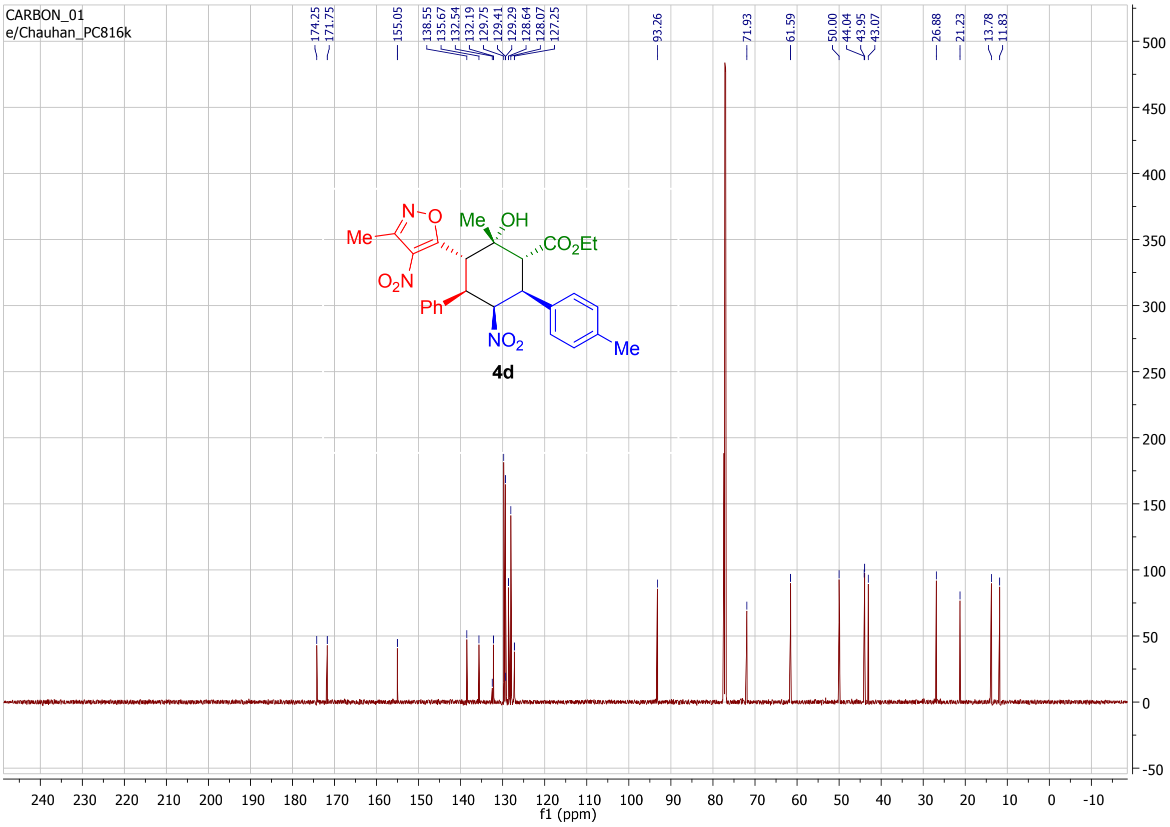
CARBON_01
e/Chauhan_PC816k
f1 (ppm)
4d

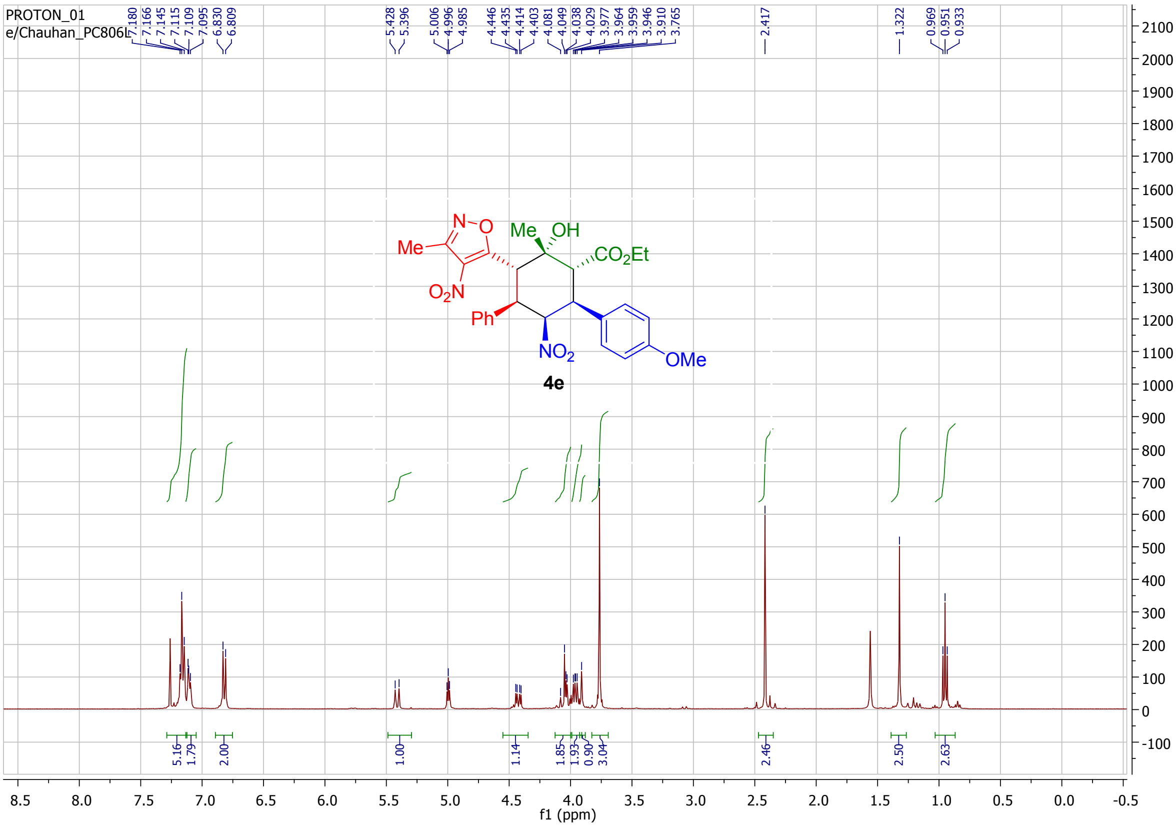
PROTON_01
e/Chauhan_PC806L
7.180 7.166 7.145 7.115 7.109 7.095 6.830 6.809
5.428 5.396 5.006 4.996 4.985
4.446 4.435 4.414 4.403 4.081 4.049 4.038 4.029 3.977 3.964 3.959 3.946 3.910 3.765
2.417
1.322 0.969 0.951 0.933
Me
N
O
Me
OH
CO2Et
O2N
Ph
NO2
OMe
4e
5.16 1.79 2.00 1.00 1.14 1.85 1.93 0.90 3.04 2.46 2.50 2.63
f1 (ppm)

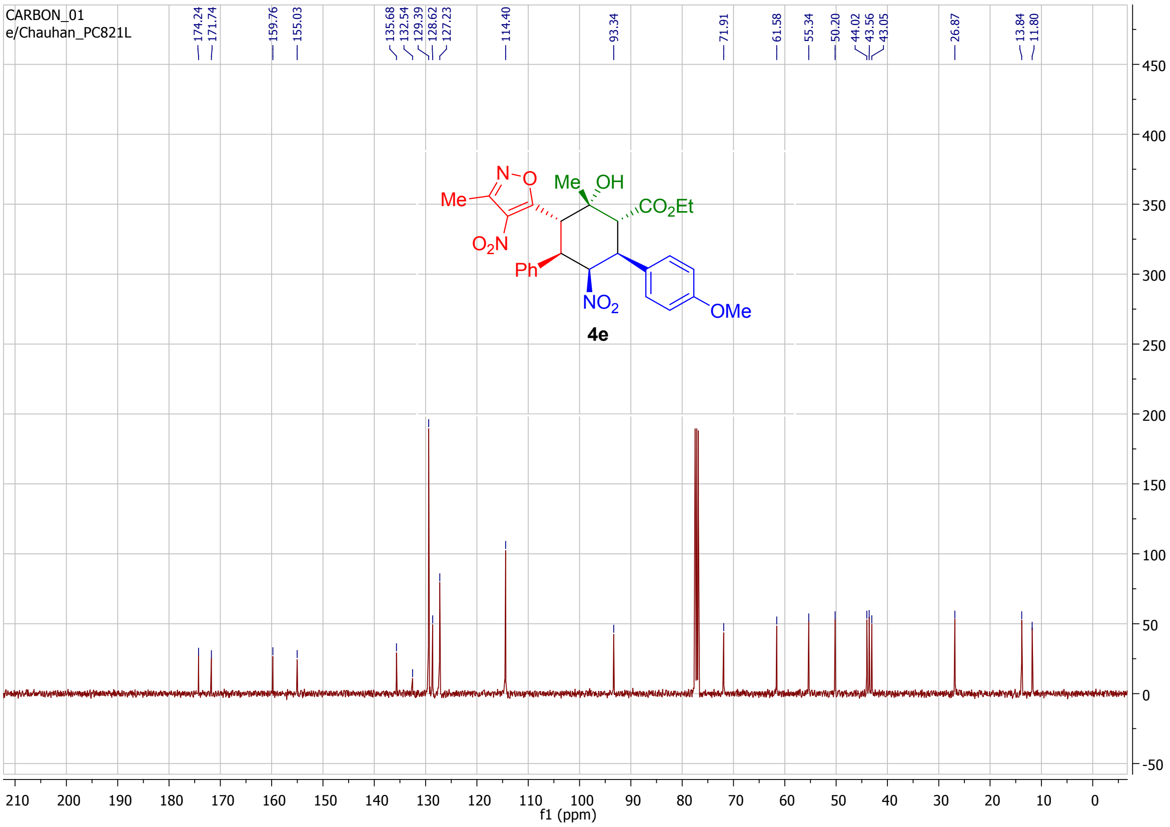
CARBON_01
e/Chauhan_PC821L
174.24 171.74 159.76 155.03
135.68 132.54 129.39 128.62 127.23
114.40
93.34
71.91
61.58 55.34 50.20 44.02 43.56 43.05
26.87
13.84 11.80
Me
N
O
Me
OH
CO2Et
O2N
Ph
NO2
OMe
4e
f1 (ppm)

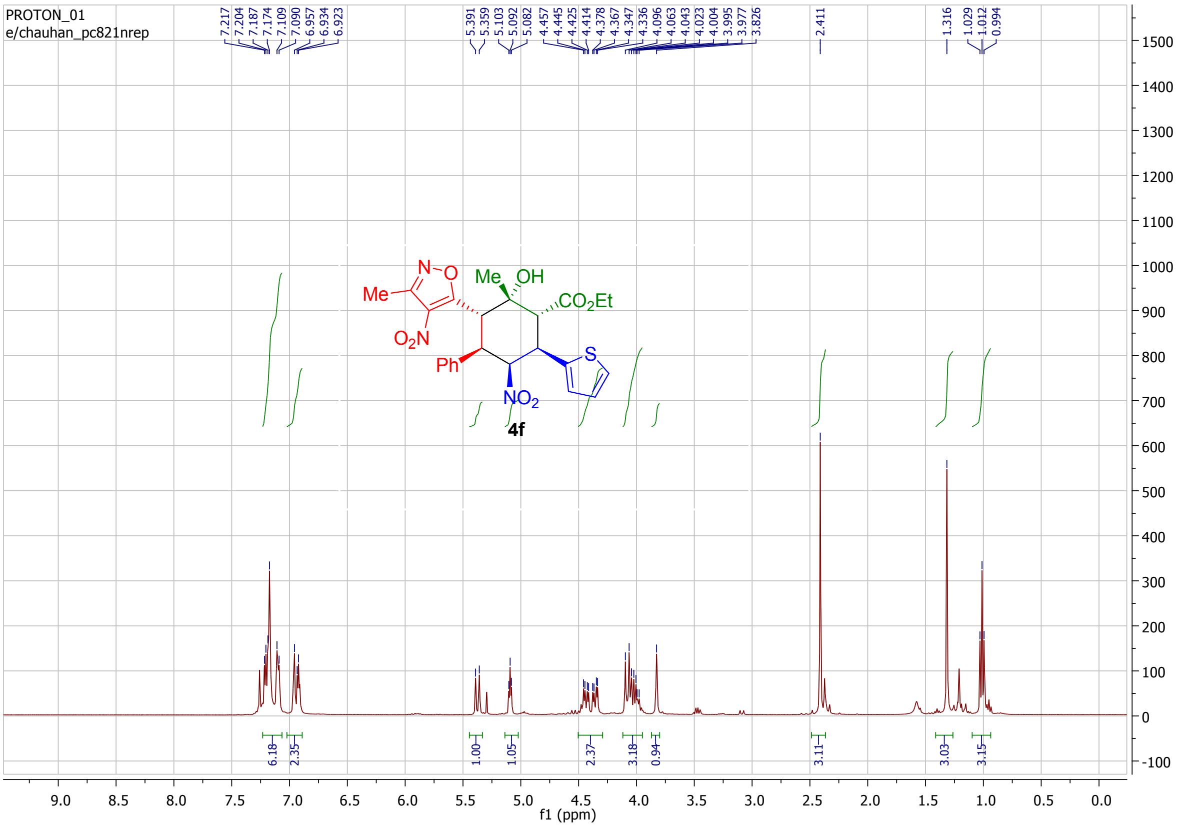
PROTON_01
e/chauhan_pc821nrep
7.217
7.204
7.187
7.174
7.109
7.090
6.957
6.934
6.923
5.391
5.359
5.103
5.092
5.082
4.457
4.445
4.425
4.414
4.378
4.367
4.347
4.336
4.096
4.063
4.043
4.023
4.004
3.995
3.977
3.826
2.411
1.316
1.029
1.012
0.994
Me
N
O
O_2N
Me
OH
CO_2Et
Ph
S
NO_2
4f
6.18
2.35
1.00
1.05
2.37
3.18
0.94
3.11
3.03
3.15
f1 (ppm)

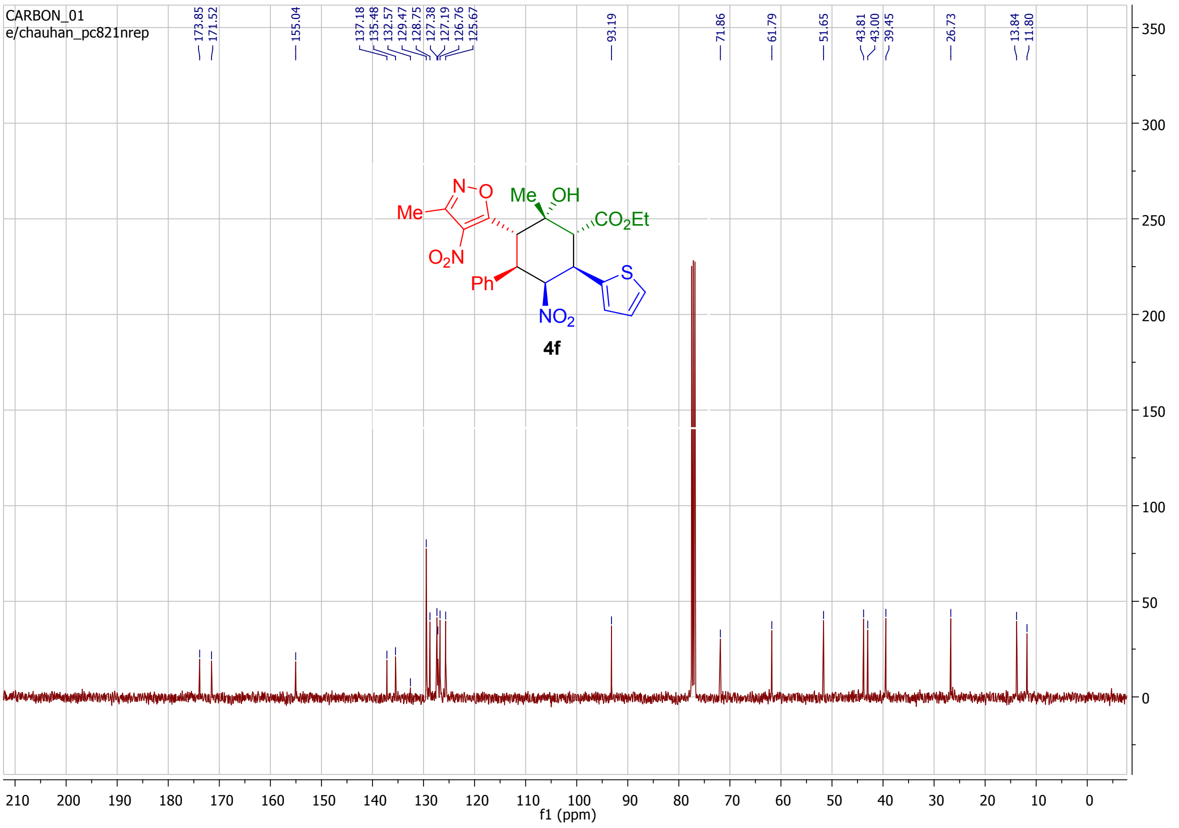
CARBON_01
e/chauhan_pc821nrep
173.85
171.52
155.04
137.18
135.48
132.57
129.47
128.75
127.38
127.19
126.76
125.67
93.19
71.86
61.79
51.65
43.81
43.00
39.45
26.73
13.84
11.80
Me
N
O
O_2N
Me
OH
CO_2Et
Ph
S
NO_2
4f
f1 (ppm)

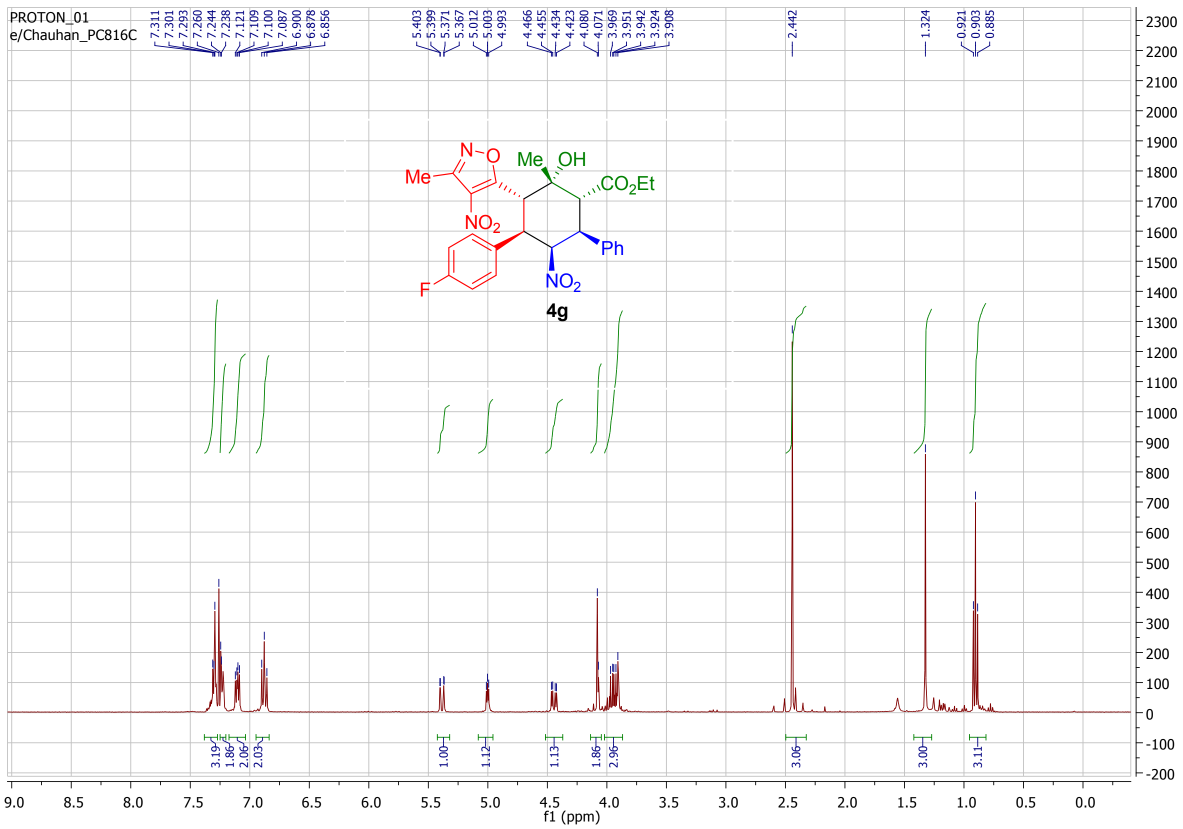
PROTON_01
e/Chauhan_PC816C
7.311
7.301
7.293
7.260
7.244
7.238
7.121
7.109
7.100
7.087
6.900
6.878
6.856
5.403
5.399
5.371
5.367
5.012
5.003
4.993
4.466
4.455
4.434
4.423
4.080
4.071
3.969
3.951
3.942
3.924
3.908
2.442
1.324
0.921
0.903
0.885
Me
OH
CO2Et
N
O
NO2
Ph
F
4g
3.19
1.86
2.06
2.03
1.00
1.12
1.13
1.86
2.96
3.06
3.00
3.11
f1 (ppm)

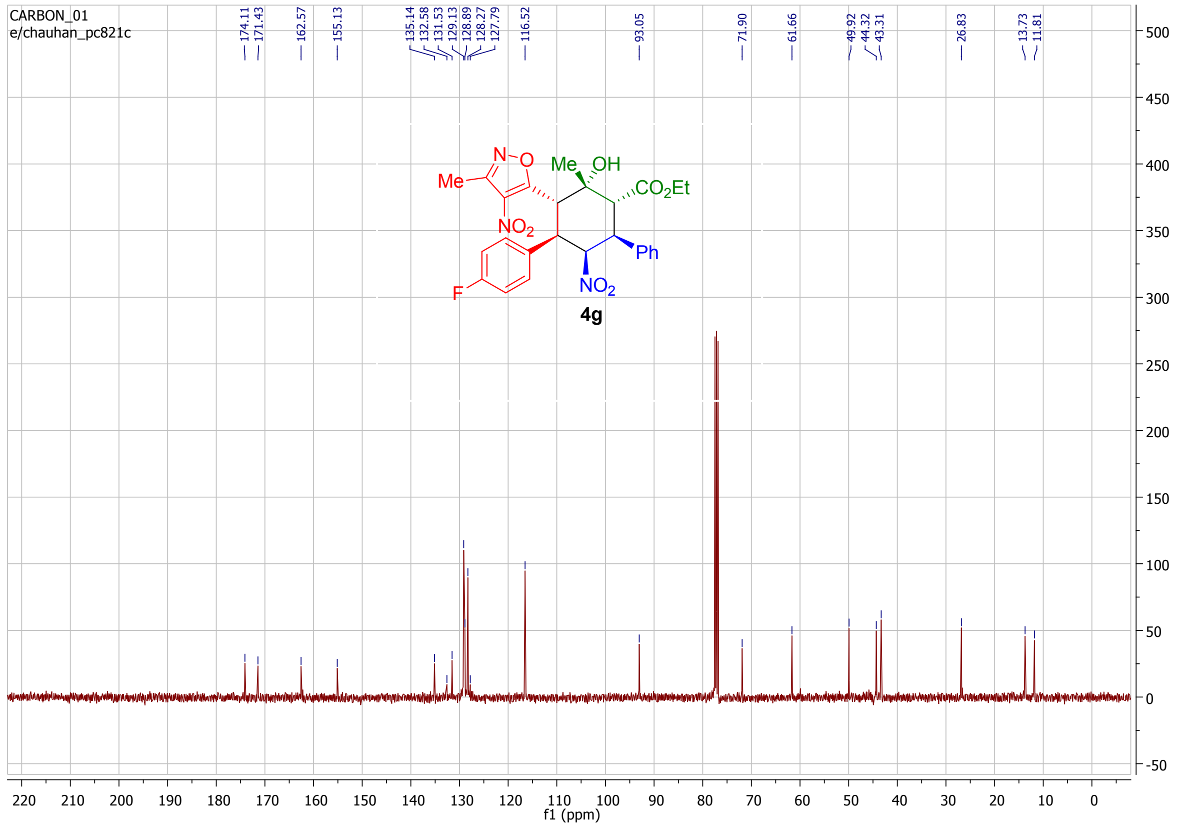
CARBON_01
e/chauhan_pc821c
174.11
171.43
162.57
155.13
135.14
132.58
131.53
129.13
128.89
128.27
127.79
116.52
93.05
71.90
61.66
49.92
44.32
43.31
26.83
13.73
11.81
Me
OH
CO2Et
N
O
NO2
Ph
F
4g
f1 (ppm)

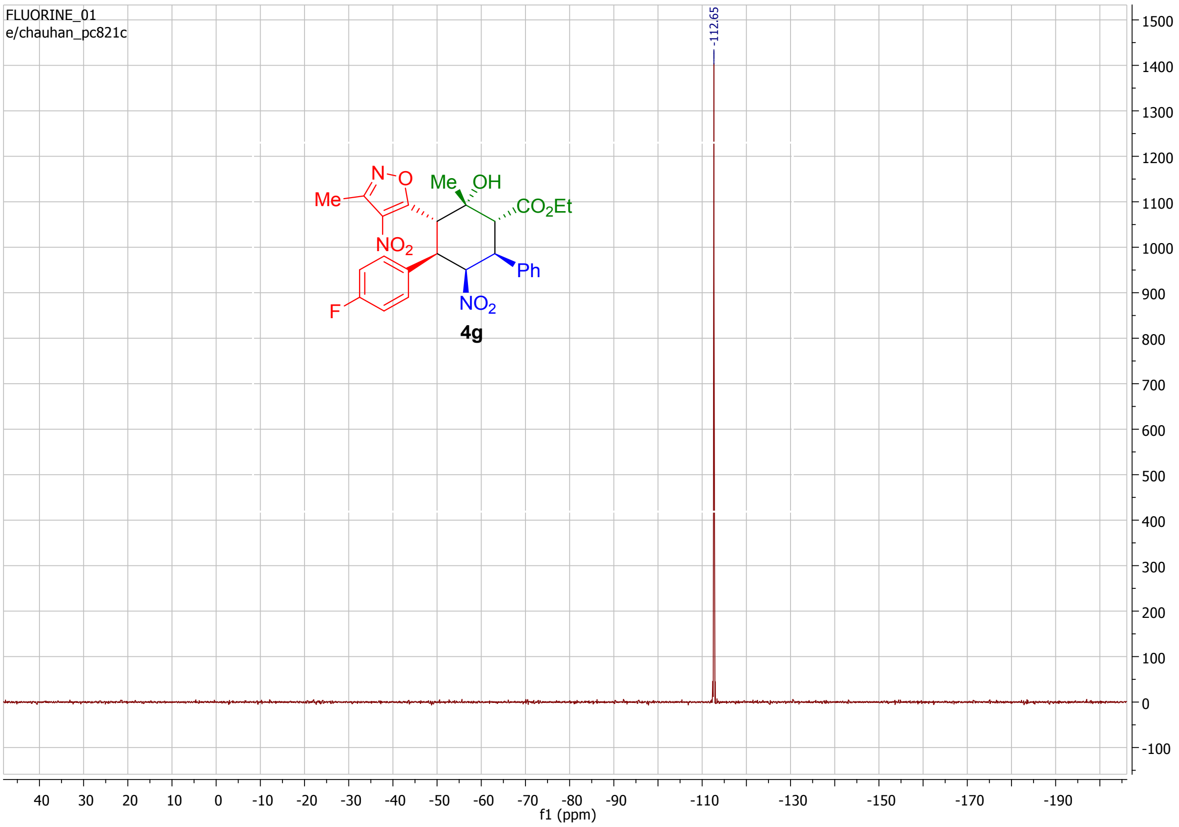
FLUORINE_01
e/chauhan_pc821c
-112.65
Me
N
O
Me
OH
CO2Et
NO2
Ph
F
NO2
4g
1500
1400
1300
1200
1100
1000
900
800
700
600
500
400
300
200
100
0
-100
40 30 20 10 0 -10 -20 -30 -40 -50 -60 -70 -80 -90 -110 -130 -150 -170 -190
f1 (ppm)

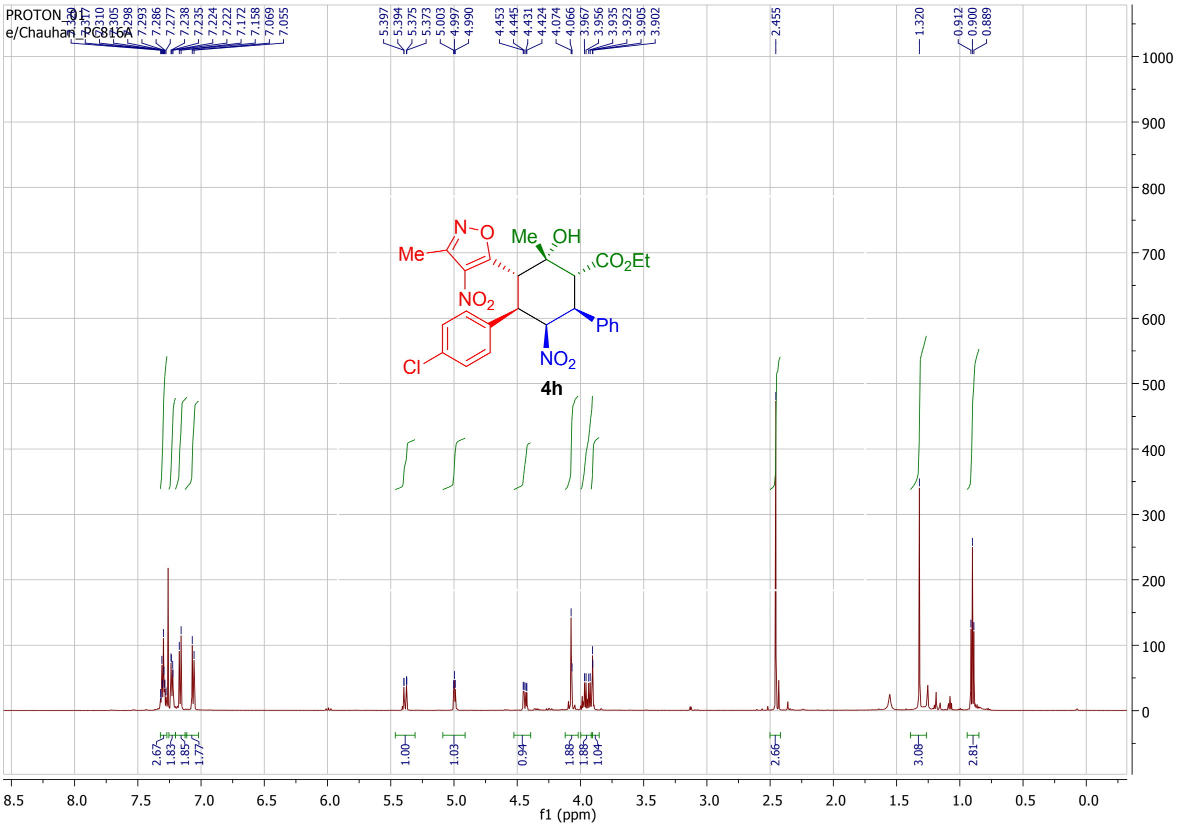
PROTON_01
e/Chauhan_PC816A
7.317
7.310
7.305
7.298
7.293
7.286
7.277
7.238
7.235
7.224
7.222
7.172
7.158
7.069
7.055
5.397
5.394
5.375
5.373
5.003
4.997
4.990
4.453
4.445
4.431
4.424
4.074
4.066
3.967
3.956
3.935
3.923
3.905
3.902
2.455
1.320
0.912
0.900
0.889
Me
N
O
NO2
Cl
Me
OH
CO2Et
Ph
NO2
4h
2.67
1.83
1.85
1.77
1.00
1.03
0.94
1.88
1.88
1.04
2.66
3.08
2.81
f1 (ppm)

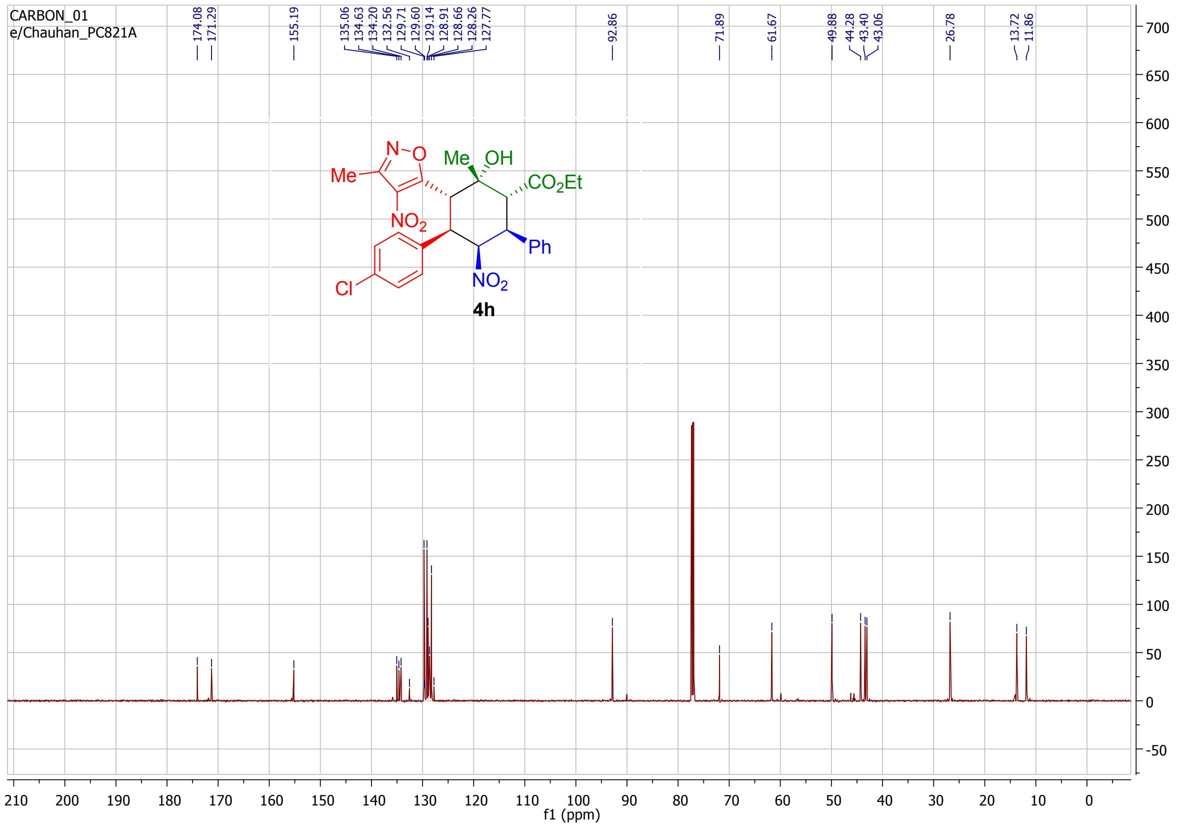
CARBON_01
e/Chauhan_PC821A
174.08
171.29
155.19
135.06
134.63
134.20
132.56
129.71
129.60
129.14
128.91
128.66
128.26
127.77
92.86
71.89
61.67
49.88
44.28
43.40
43.06
26.78
13.72
11.86
Me
N
O
NO2
Cl
Me
OH
CO2Et
Ph
NO2
4h
f1 (ppm)

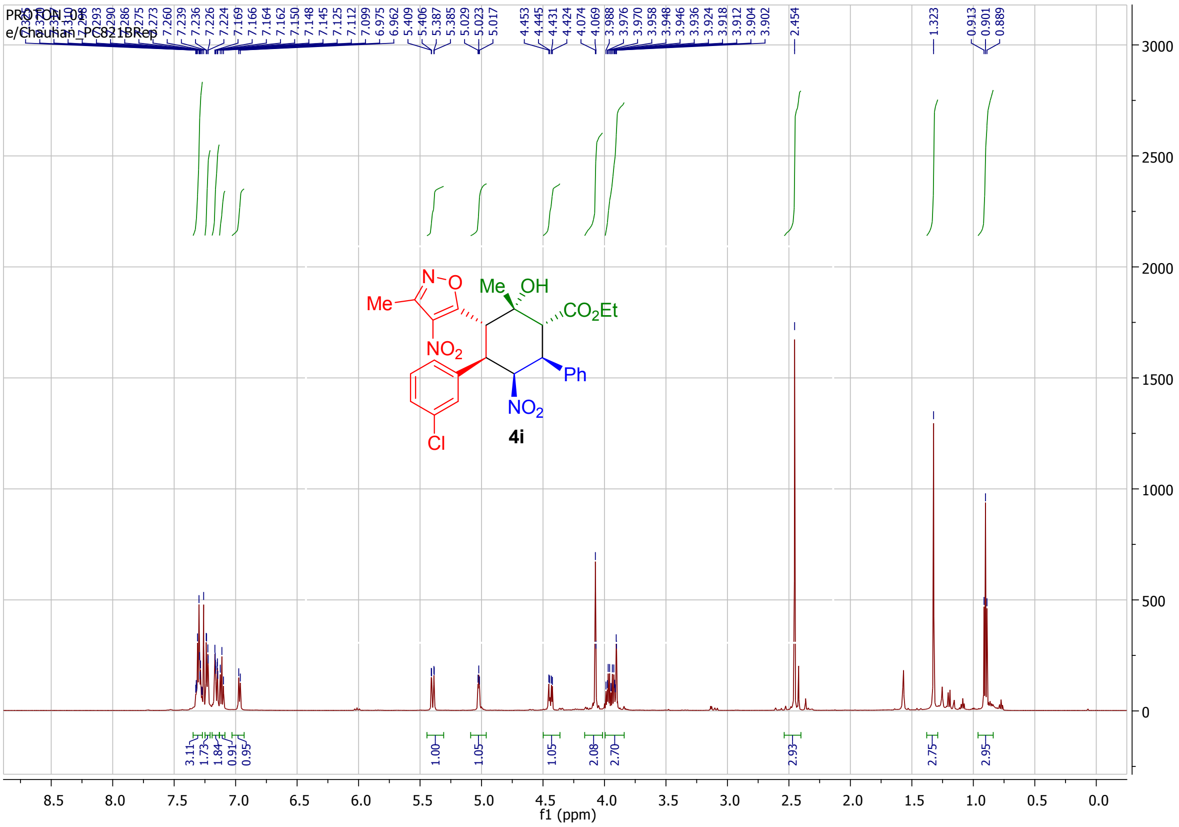
PROTON_01
e/Chauhan_PC821BRep
4i
f1 (ppm)

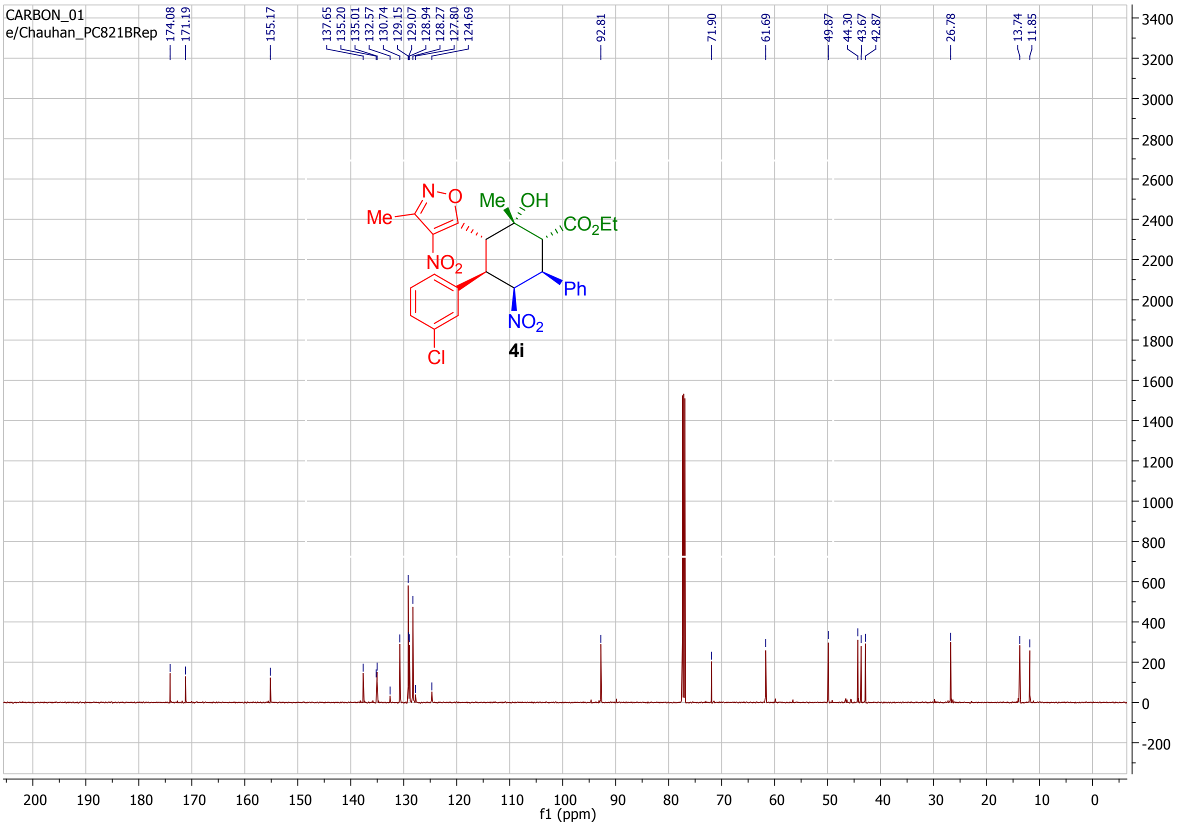
CARBON_01
e/Chauhan_PC821BRep
174.08
171.19
155.17
137.65
135.20
135.01
132.57
130.74
129.15
129.07
128.94
128.27
127.80
124.69
92.81
71.90
61.69
49.87
44.30
43.67
42.87
26.78
13.74
11.85
4i
f1 (ppm)

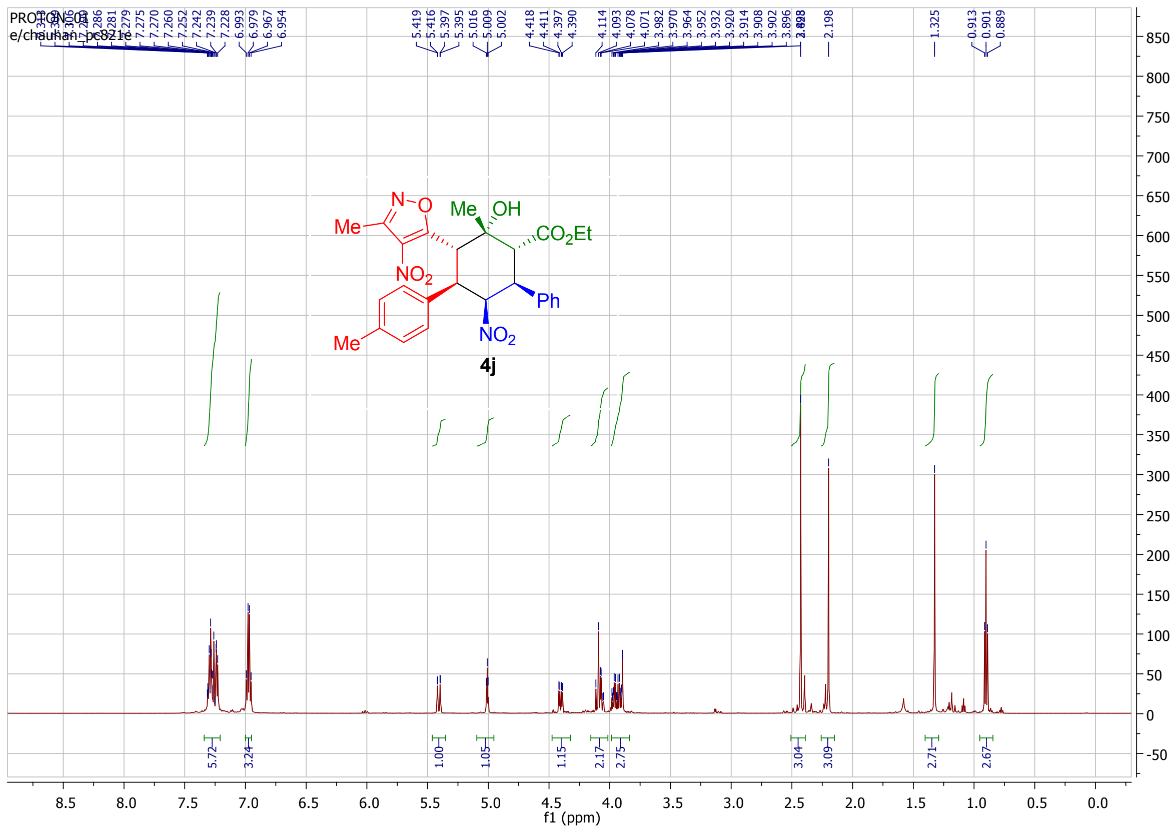

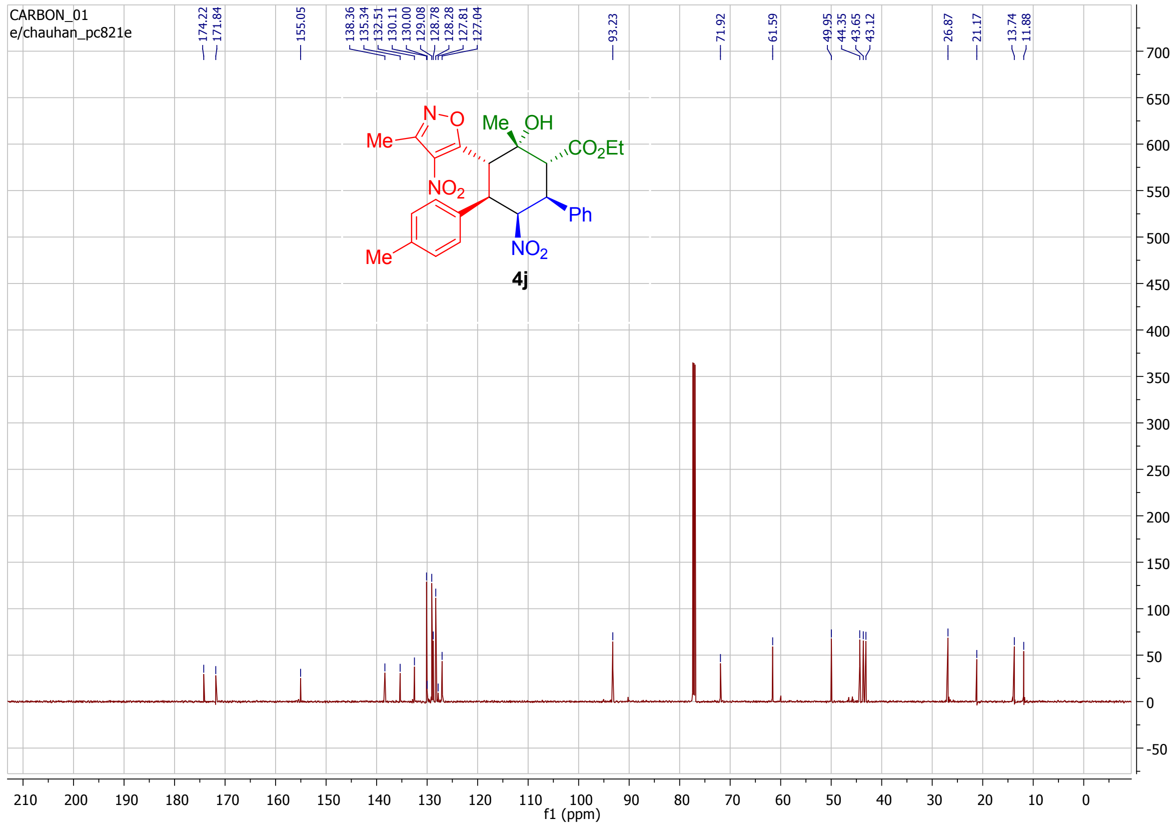

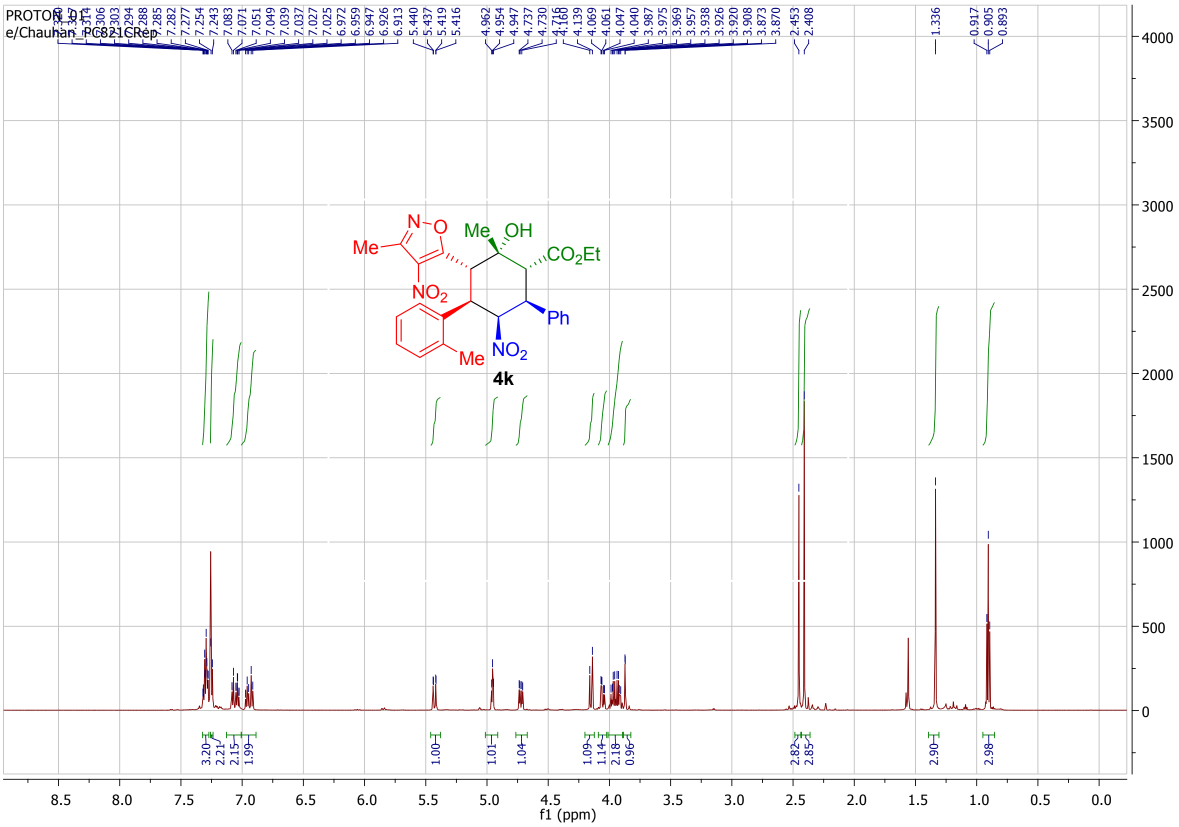

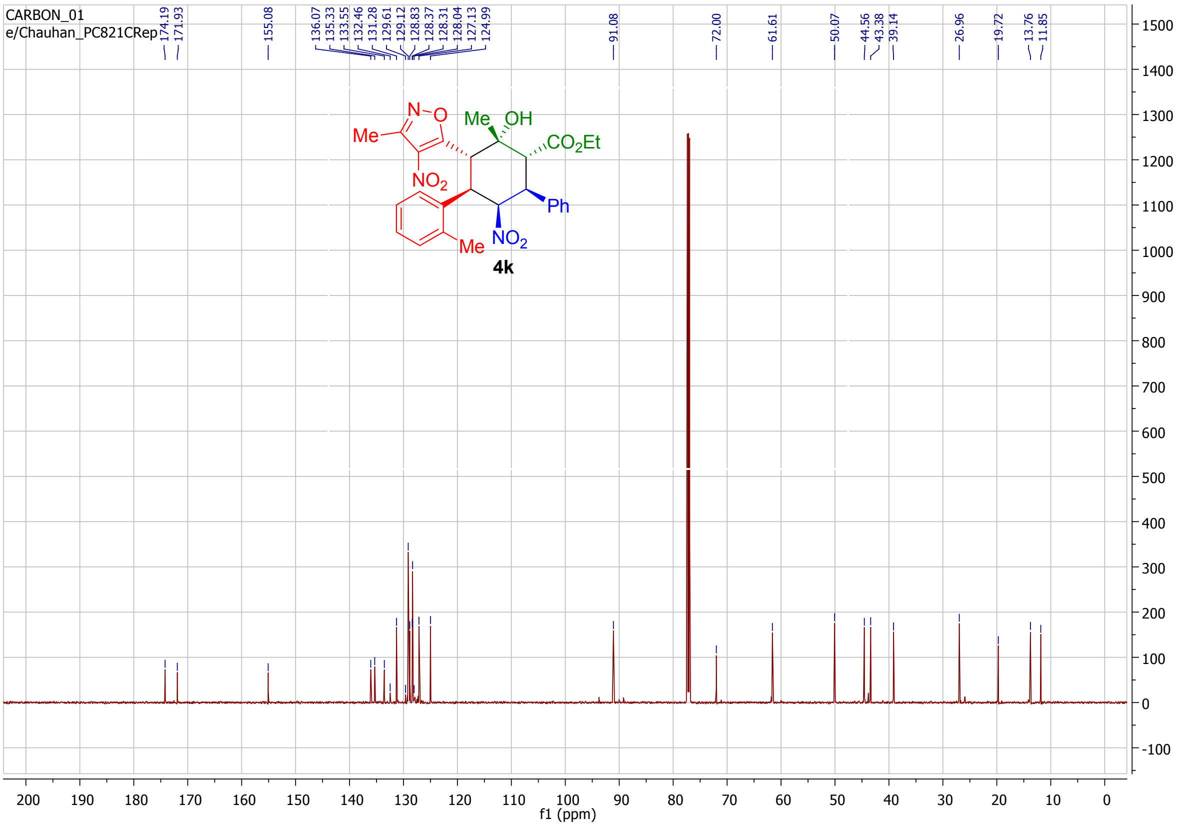

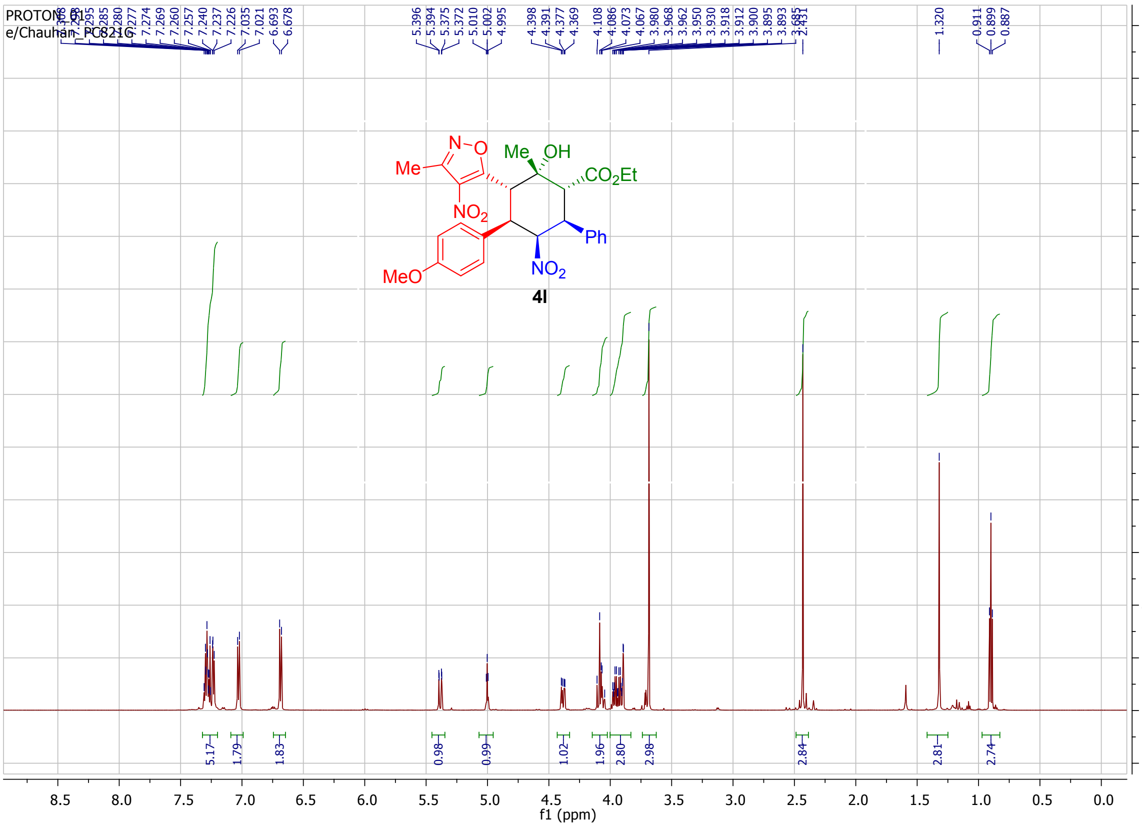
PROTON_01
e/Chauhan_PC821G
4l
f1 (ppm)

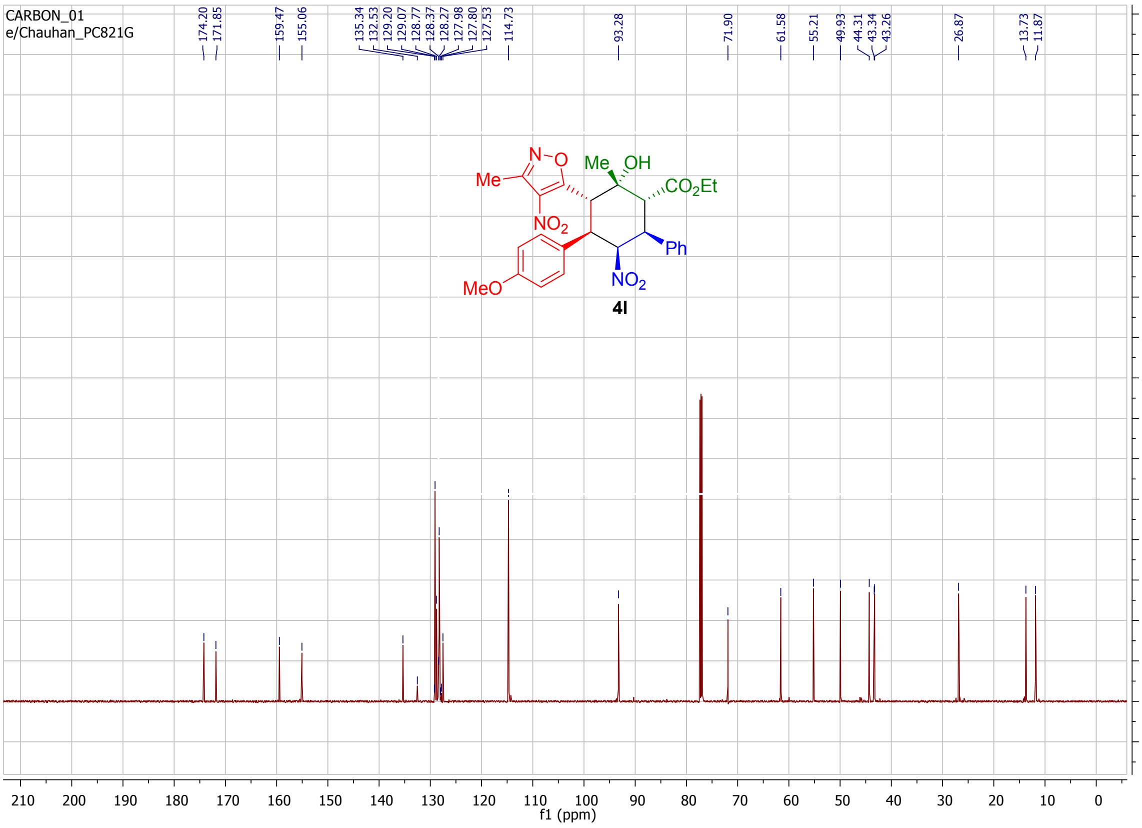
CARBON_01
e/Chauhan_PC821G
174.20
171.85
159.47
155.06
135.34
132.53
129.20
129.07
128.77
128.37
128.27
127.98
127.80
127.53
114.73
93.28
71.90
61.58
55.21
49.93
44.31
43.34
43.26
26.87
13.73
11.87
4l
f1 (ppm)

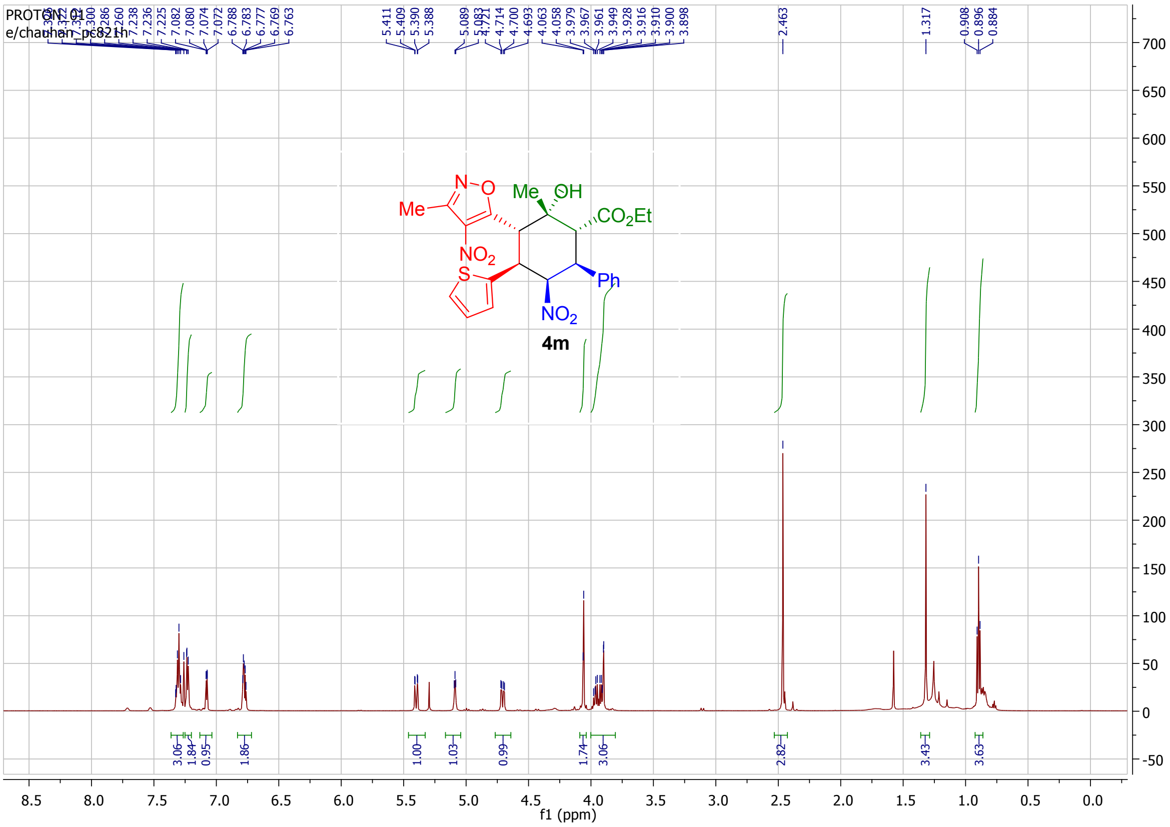
PROTON_01
5.411
5.409
5.390
5.388
5.089
4.714
4.700
4.693
4.063
4.058
3.979
3.967
3.961
3.949
3.928
3.916
3.910
3.900
3.898
2.463
1.317
0.908
0.896
0.884
Me
N
O
NO2
S
Me
OH
CO2Et
Ph
NO2
4m
3.06
1.84
0.95
1.86
1.00
1.03
0.99
1.74
3.06
2.82
3.43
3.63
f1 (ppm)

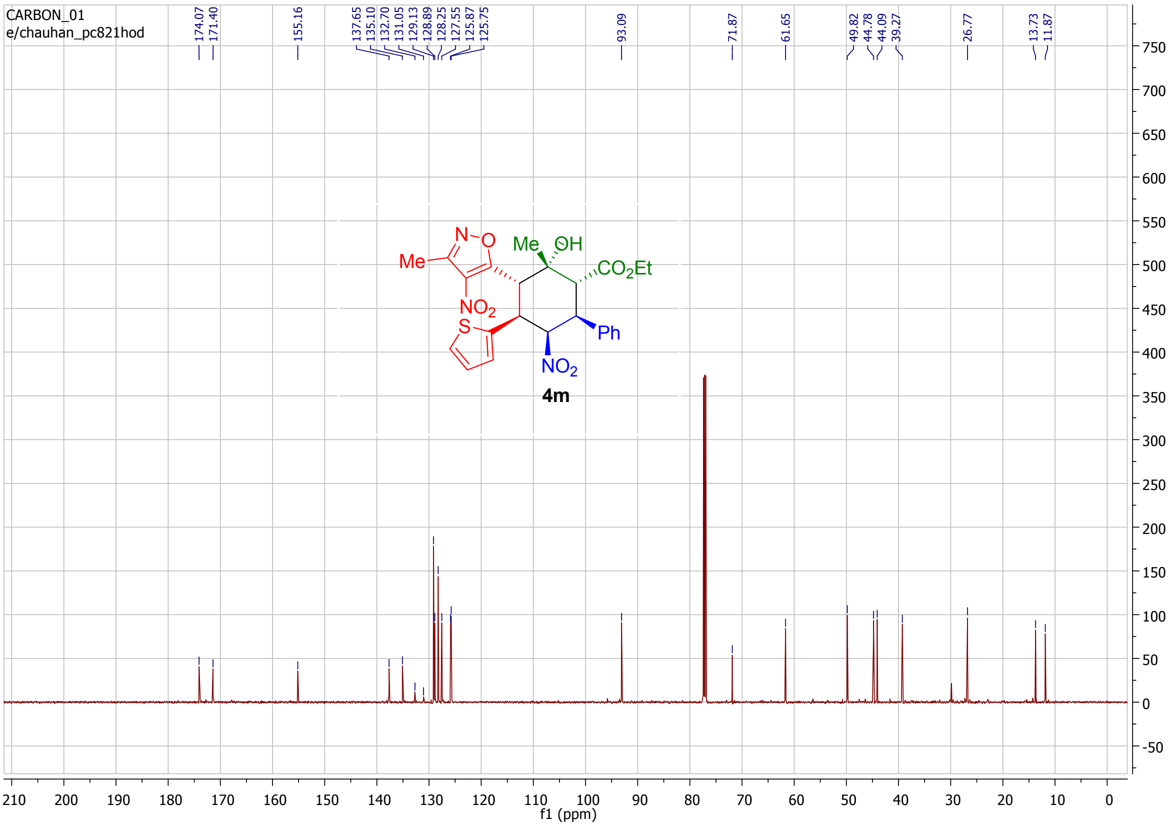
CARBON_01
e/chauhan_pc821hod
174.07
171.40
155.16
137.65
135.10
132.70
131.05
129.13
128.89
128.25
127.55
125.87
125.75
93.09
71.87
61.65
49.82
44.78
44.09
39.27
26.77
13.73
11.87
Me
N
O
NO2
S
Me
OH
CO2Et
Ph
NO2
4m
f1 (ppm)

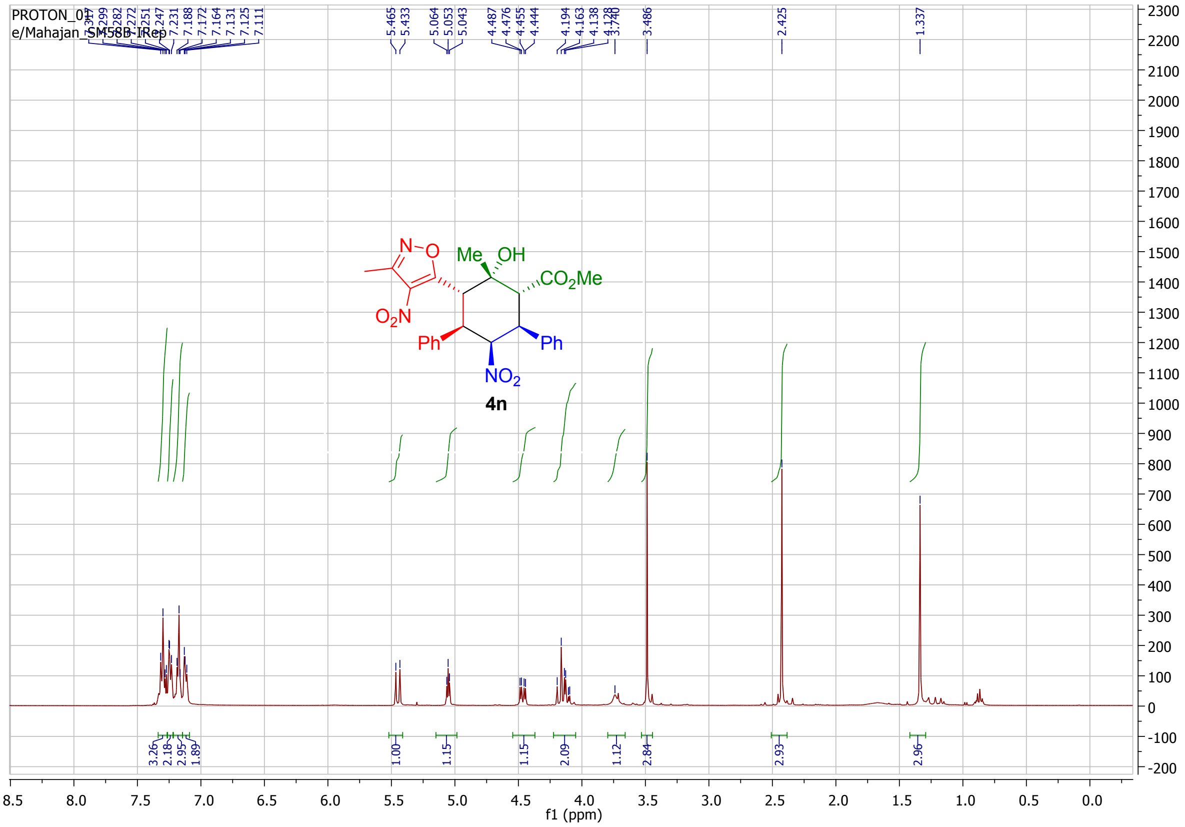
PROTON_01
e/Mahajan_SM58B-IRep
4n
f1 (ppm)

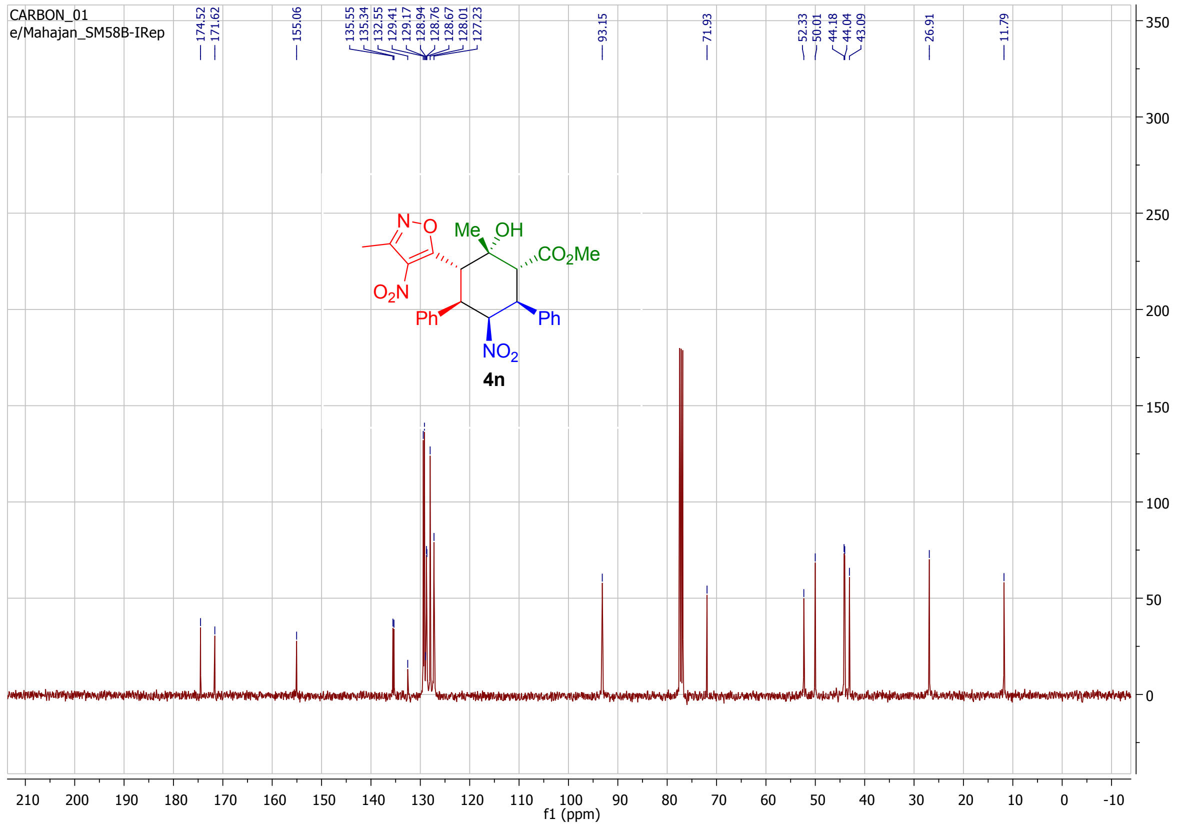
CARBON_01
e/Mahajan_SM58B-IRep
4n
f1 (ppm)

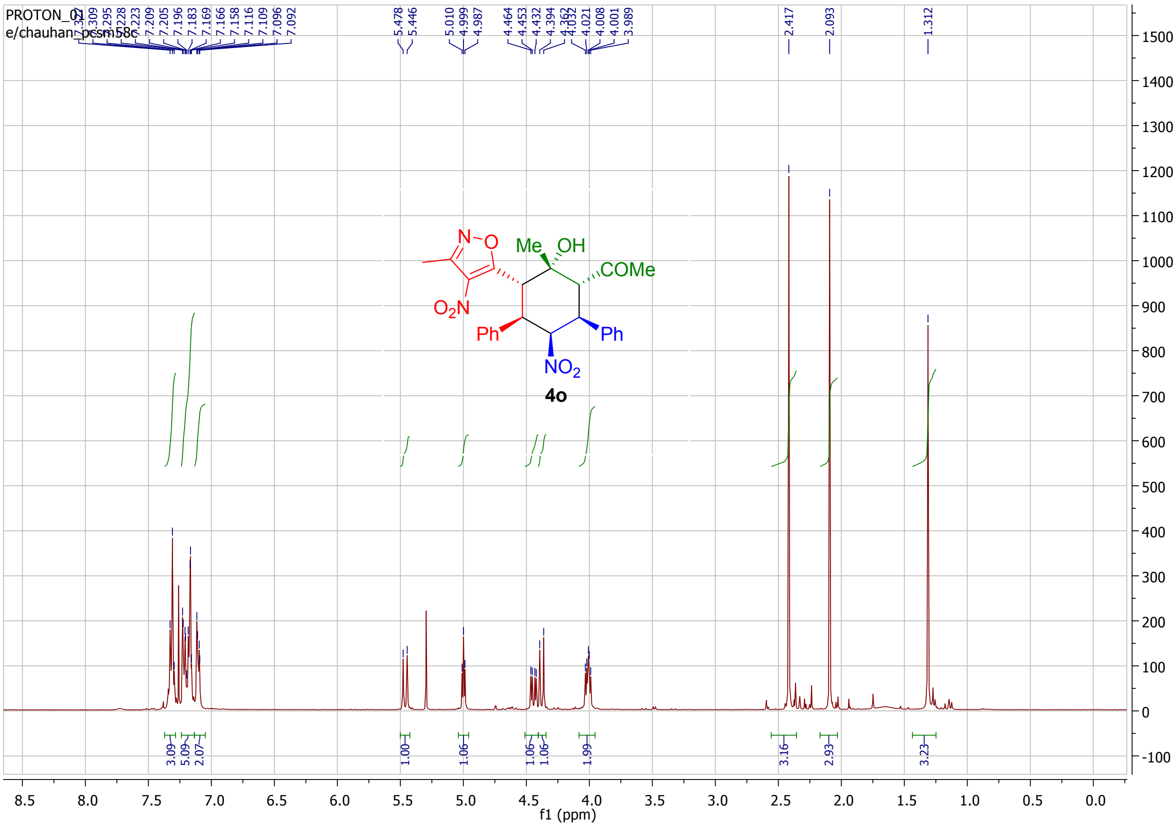
PROTON_01
e/chauhan_pcsm58c
7.309
7.295
7.228
7.223
7.209
7.205
7.196
7.183
7.169
7.166
7.158
7.116
7.109
7.096
7.092
5.478
5.446
5.010
4.999
4.987
4.464
4.453
4.432
4.394
4.362
4.032
4.021
4.008
4.001
3.989
2.417
2.093
1.312
3.09
5.09
2.07
1.00
1.06
1.06
1.06
1.99
3.16
2.93
3.23
f1 (ppm)
N-O
Me
OH
COMe
O_2N
Ph
Ph
NO_2
4o

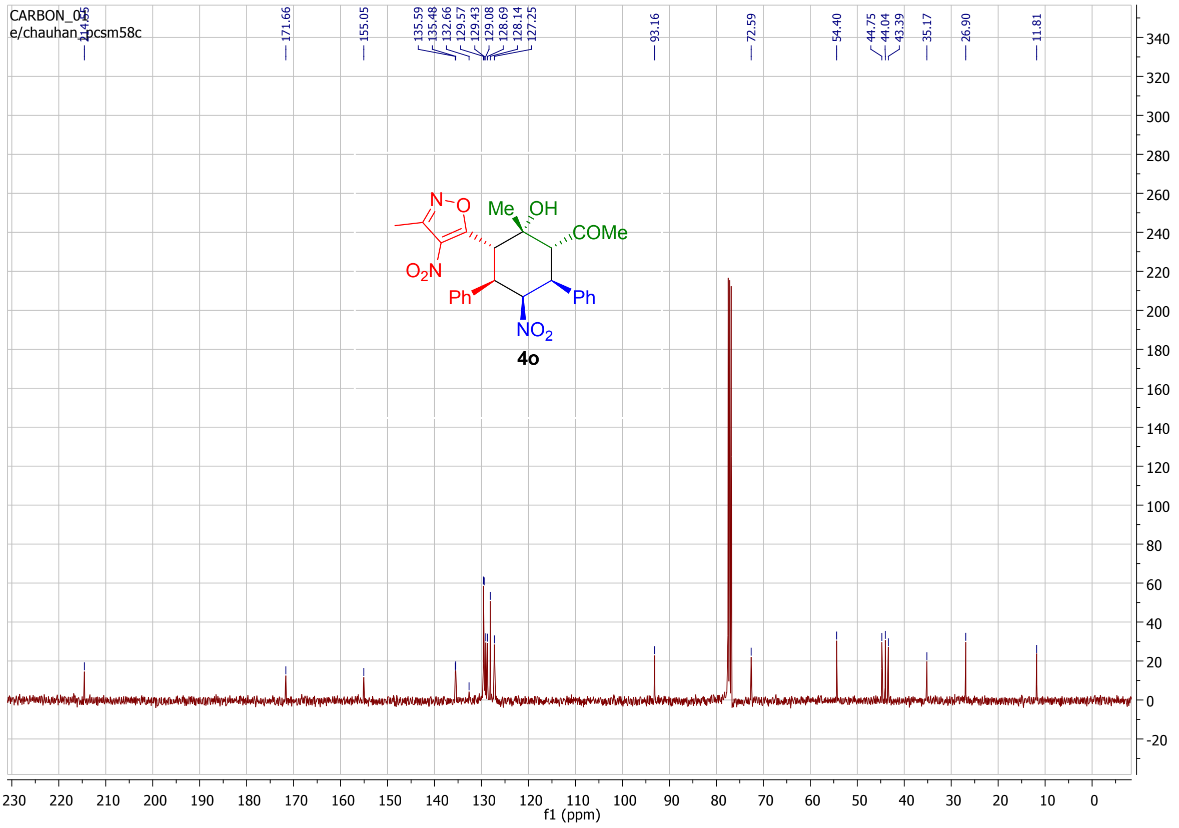
CARBON_01
e/chauhan_pcsm58c
171.66
155.05
135.59
135.48
132.66
129.57
129.43
129.08
128.69
128.14
127.25
93.16
72.59
54.40
44.75
44.04
43.39
35.17
26.90
11.81
f1 (ppm)
N-O
Me
OH
COMe
O_2N
Ph
Ph
NO_2
4o

HPLC Data

Sample Name: SM 38 rac
Data file: F:\GONZO\SM\38R3AD.D
Sample Info: Laufmittel: n-Heptan/IP 7:3; Die Probe ist in EtOH/LM gelöst.

HEWLETT PACKARD

Säule: DAICELAD.M
Säuleninfo: Chiralpak AD (250x4,6)mm
Operator: Analytik Labor AKEN

Injektion Time: 08:35:36
Injektion Date: 13.12.2013

Instrument Conditions:	At Start	At Stop
Temperature in°C:	30.0°C	30.0°C
Pressure in bar:	36.1	37.1
Flow in ml/min:	1.00	1.00

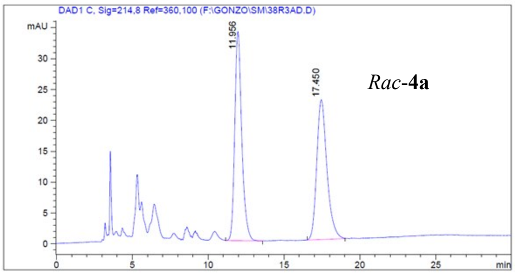

#	Ret. Time (min)	Width	Height (mAU)	Area (mAU*s)	Area %
1	11.96	0.49	33.89	1088.11	50.68
2	17.45	0.71	22.65	1058.89	49.32
Total				2147.00	100.00

Sample Name: PC 822 A
Data file: D:\GONZO\PC\822AAD.D
Sample Info: Laufmittel: n-Heptan/iPrOH 7:3; Die Probe ist in LM/DCM gelöst

HEWLETT PACKARD

Säule: DAICELAD.M
Säuleninfo: Chiralpak AD (250x4,6)mm
Operator: Analytik Labor AKEN

Injektion Time: 12:21:04
Injektion Date: 20.11.2014

Instrument Conditions:	At Start	At Stop
Temperature in°C:	30.0°C	30.0°C
Pressure in bar:	34.5	34.9
Flow in ml/min:	1.00	1.00

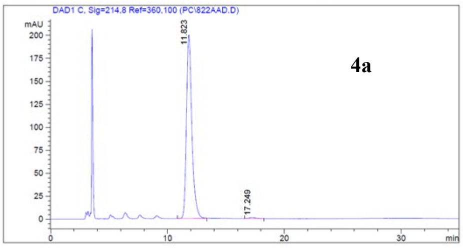

#	Ret. Time (min)	Width	Height (mAU)	Area (mAU*s)	Area %
1	11.82	0.48	199.82	6413.34	99.28
2	17.25	0.52	1.08	46.32	0.72
Total				6459.67	100.00

Sample Name: PC 806 J (qd)
Data file: D:\GONZO\PC\806JQDAD.D
Sample Info: Laufmittel: n-Heptan/iPrOH 7:3; Die Probe ist in DCM/LM gelöst.

HEWLETT PACKARD

Säule: DAICELAD.M
Säuleninfo: Chiralpak AD (250x4,6)mm
Operator: Analytik Labor AKEN

Injektion Time: 13:51:58
Injektion Date: 24.11.2014

Instrument Conditions:	At Start	At Stop
Temperature in°C:	30.0°C	30.0°C
Pressure in bar:	34.4	34.5
Flow in ml/min:	1.00	1.00

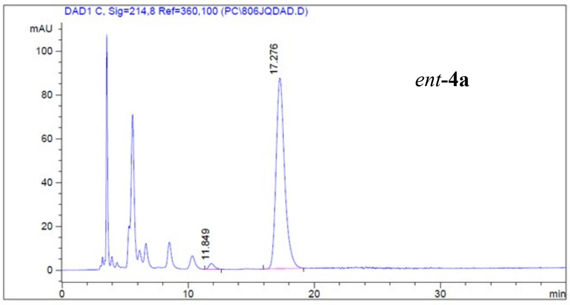

#	Ret. Time (min)	Width	Height (mAU)	Area (mAU*s)	Area %
1	11.85	0.45	2.67	80.56	1.92
2	17.28	0.71	87.04	4105.44	98.08
Total				4186.00	100.00

Sample name: PC 816 +821 I
Data file: C:\SNOOPY\PC\8136821IIC.D
Description: Laufmittel: n-Heptan/iPrOH 9:1; Probe ist in LM/DCM gelöst
Injection date: 11/11/2014 8:34:53 AM
Acq. Analysis method: CHIRALPAKIC1-6LNP.M

Column: Chiralpak IC, (150 x 4,6) mm, 5μ, SN: IC00CD-QF015

Pressure at start: 25 bar **Start flow:** 0.700 ml/min **Column oven:** 22.67 °C

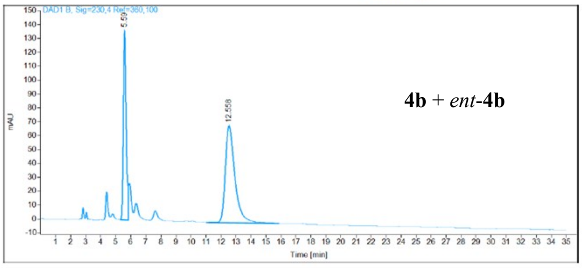

Name PC 816 +821 I

RT [min]	Type	Area%	Area	Height	Width [min]
5.59	BV	36.77	1807.43	136.54	0.20
12.56	BB	63.23	3107.61	69.80	0.67
	Sum	100.00	4915.04		

Sample name: PC 821 IA
Data file: C:\SNOOPY\PC\821IAIC.D
Description: Laufmittel: n-Heptan/iPrOH 9:1; Probe ist in LM/DCM gelöst
Injection date: 11/24/2014 11:05:10 AM
Acq. Analysis method: CHIRALPAKIC1-6LNP.M

Column: Chiralpak IC, (150 x 4,6) mm, 5μ, SN: IC00CD-QF015

Pressure at start: 27 bar **Start flow:** 0.700 ml/min **Column oven:** 23 °C

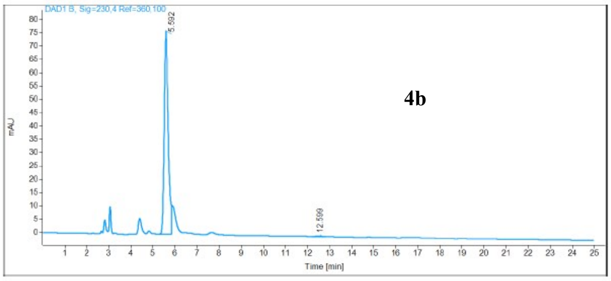

Name PC 821 IA

RT [min]	Type	Area%	Area	Height	Width [min]
5.59	MF	99.64	1000.98	76.31	0.22
12.60	MM	0.36	3.63	0.15	0.40
	Sum	100.00	1004.60		

Sample name: PC 816 I
Data file: C:\SNOOPY\PC\816I2IC.D
Description: Laufmittel: n-Heptan/iPrOH 9:1; Probe ist in LM/DCM gelöst
Injection date: 11/10/2014 4:06:17 PM
Acq. Analysis method: CHIRALPAKIC1-6LNP.M

Column: Chiralpak IC, (150 x 4,6) mm, 5μ, SN: IC00CD-QF015

Pressure at start: 24 bar **Start flow:** 0.700 ml/min **Column oven:** 27.5 °C

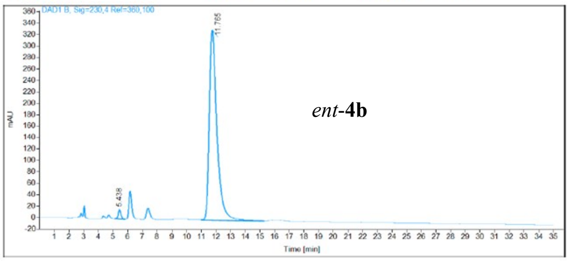

Name PC 816 I

RT [min]	Type	Area%	Area	Height	Width [min]
5.44	BB	1.58	192.14	15.80	0.18
11.76	VB	98.42	11956.05	332.14	0.54
	Sum	100.00	12148.19		

Sample name: PC 816 + 821 J

Data file: C:\SNOOPY\PC\816821JIC.D
Description: Laufmittel: n-Heptan/iPrOH 9:1; Probe ist in LM/DCM gelöst

Injection date: 11/11/2014 9:11:01 AM
Acq. Analysis method: CHIRALPAKIC1-6LNP.M

Column: Chiralpak IC, (150 x 4,6) mm, 5µ, SN: IC00CD-QF015

Pressure at start: 25 bar **Start flow:** 0.700 ml/min **Column oven:** 22.83 °C

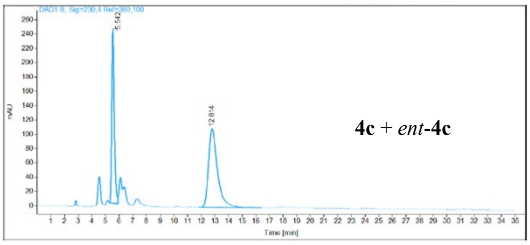

Name PC 816 + 821 J

RT [min]	Type	Area%	Area	Height	Width [min]
5.54	BV	39.51	3255.37	244.27	0.20
12.81	BB	60.49	4983.30	108.86	0.69
Sum		100.00	8238.67		

Sample name: PC 821 J rep

Data file: C:\SNOOPY\PC\PC 821 J REP IC.D
Description: Laufmittel: n-Heptan/iPrOH 9:1 Die Probe ist DCM/LM gelöst.

Injection date: 11/17/2014 11:45:20 AM
Acq. Analysis method: CHIRALPAKIC1-6LNP.M

Column: Chiralpak IC, (150 x 4,6) mm, 5µ, SN: IC00CD-QF015

Pressure at start: 25 bar **Start flow:** 0.700 ml/min **Column oven:** 22.85 °C

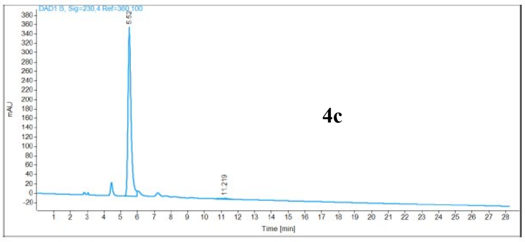

Name PC 821 J rep

RT [min]	Type	Area%	Area	Height	Width [min]
5.52	BV	99.13	4941.44	360.37	0.21
11.22	BBA	0.87	43.35	1.53	0.39
Sum		100.00	4984.79		

Sample name: PC 816 J

Data file: C:\SNOOPY\PC\PC 816 J 1IC.D
Description: Laufmittel: n-Heptan/iPrOH 9:1 Die Probe ist DCM/LM gelöst.

Injection date: 11/11/2014 1:00:13 PM
Acq. Analysis method: CHIRALPAKIC1-6LNP.M

Column: Chiralpak IC, (150 x 4,6) mm, 5µ, SN: IC00CD-QF015

Pressure at start: 25 bar **Start flow:** 0.700 ml/min **Column oven:** 23.24 °C

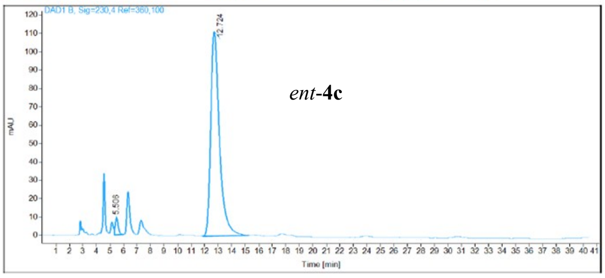

Name PC 816 J

RT [min]	Type	Area%	Area	Height	Width [min]
5.51	VB	2.71	138.50	9.24	0.22
12.72	BB	97.29	4968.67	111.29	0.67
Sum		100.00	5107.17		

Sample Name: PC 816K + 821 K
Data file: D:\GONZO\PC\81621KAD.D
Sample Info: Laufmittel: n-Heptan/iPrOH 7:3;
Die Probe ist in DCM/LM gelöst.

HEWLETT PACKARD

Säule: DAICELAD.M
Säuleninfo: Chiralpak AD (250x4,6)mm
Operator: Analytik Labor AKEN

Injektion Time: 08:35:10
Injektion Date: 10.11.2014

Instrument Conditions:	At Start	At Stop
Temperature in°C:	30.0°C	30.0°C
Pressure in bar:	22.7	22.8
Flow in ml/min:	0.70	0.70

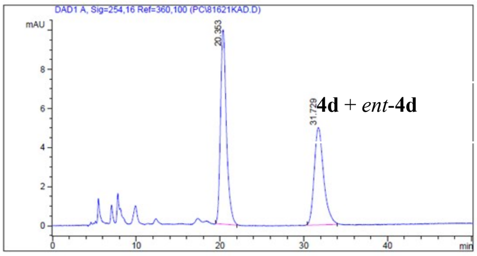

#	Ret. Time (min)	Width	Height (mAU)	Area (mAU*s)	Area %
1	20.35	0.74	9.95	489.81	55.69
2	31.73	1.12	4.98	389.73	44.31
Total				879.54	100.00

Sample Name: PC 821 K
Data file: D:\GONZO\PC\821KAD.D
Sample Info: Laufmittel: n-Heptan/iPrOH 7:3;
Die Probe ist in LM/DCM gelöst

HEWLETT PACKARD

Säule: DAICELAD.M
Säuleninfo: Chiralpak AD (250x4,6)mm
Operator: Analytik Labor AKEN

Injektion Time: 14:23:54
Injektion Date: 07.11.2014

Instrument Conditions:	At Start	At Stop
Temperature in°C:	30.0°C	30.0°C
Pressure in bar:	22.2	23.1
Flow in ml/min:	0.70	0.70

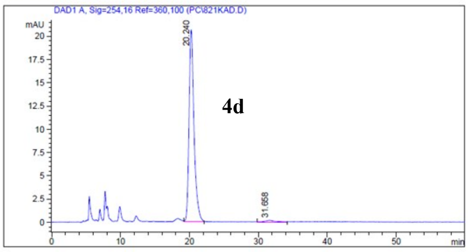

#	Ret. Time (min)	Width	Height (mAU)	Area (mAU*s)	Area %
1	20.24	0.76	20.64	1035.65	96.71
2	31.66	2.34	0.25	35.28	3.29
Total				1070.93	100.00

Sample Name: PC 816 K
Data file: D:\GONZO\PC\816KAD.D
Sample Info: Laufmittel: n-Heptan/iPrOH 7:3;
Die Probe ist in LM/DCM gelöst

HEWLETT PACKARD

Säule: DAICELAD.M
Säuleninfo: Chiralpak AD (250x4,6)mm
Operator: Analytik Labor AKEN

Injektion Time: 13:22:39
Injektion Date: 07.11.2014

Instrument Conditions:	At Start	At Stop
Temperature in°C:	30.0°C	30.0°C
Pressure in bar:	22.6	22.7
Flow in ml/min:	0.70	0.70

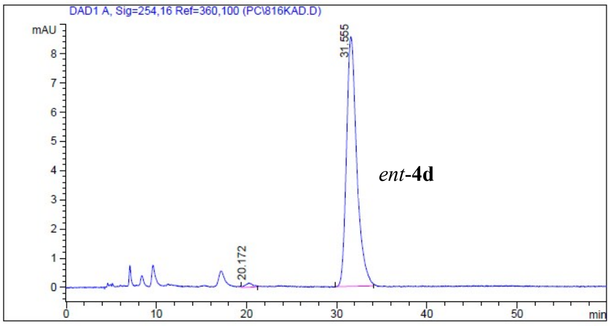

#	Ret. Time (min)	Width	Height (mAU)	Area (mAU*s)	Area %
1	20.17	0.96	0.14	8.20	1.19
2	31.56	1.04	8.55	679.06	98.81
Total				687.26	100.00

Sample name: PC 816+821 L
Data file: C:\SNOOPY\PC\816821LIA.D
Description: Laufmittel: n-Heptan/EtOH 9:1; Probe ist in LM/DCM gelöst.
Injection date: 11/13/2014 11:22:27 AM
Acq. Analysis method: CHIRALPAKIARNP.M
Column: Chiralpak IA, (250 x 4,6) mm, 5µ, SN: IA00CE-RC036
Pressure at start: 51 bar **Start flow:** 1.000 ml/min **Column oven:** 30 °C

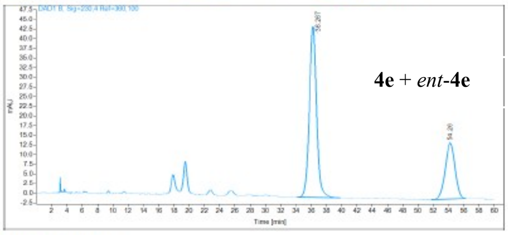

Name PC 816+821 L

RT [min]	Type	Area%	Area	Height	Width [min]
36.27	BB	69.27	2933.85	44.22	1.01
54.26	BB	30.73	1301.79	14.49	1.40
	Sum	100.00	4235.64		

Sample name: PC 821 L
Data file: C:\SNOOPY\PC\821LIA1.D
Description: Laufmittel: n-Heptan/EtOH 9:1; Probe ist in LM/DCM gelöst.
Injection date: 11/13/2014 4:44:19 PM
Acq. Analysis method: CHIRALPAKIARNP.M
Column: Chiralpak IA, (250 x 4,6) mm, 5µ, SN: IA00CE-RC036
Pressure at start: 50 bar **Start flow:** 1.000 ml/min **Column oven:** 30 °C

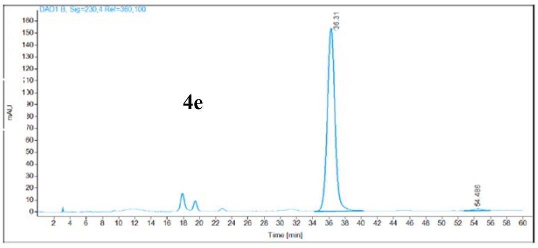

Name PC 821 L

RT [min]	Type	Area%	Area	Height	Width [min]
36.31	BB	98.86	10098.42	153.32	1.01
54.49	BB	1.14	116.76	1.36	1.03
	Sum	100.00	10215.18		

Sample name: PC 816 L
Data file: C:\SNOOPY\PC\816LIA.D
Description: Laufmittel: n-Heptan/EtOH 9:1; Probe ist in LM/DCM gelöst.
Injection date: 11/13/2014 3:43:13 PM
Acq. Analysis method: CHIRALPAKIARNP.M
Column: Chiralpak IA, (250 x 4,6) mm, 5µ, SN: IA00CE-RC036
Pressure at start: 52 bar **Start flow:** 1.000 ml/min **Column oven:** 30 °C

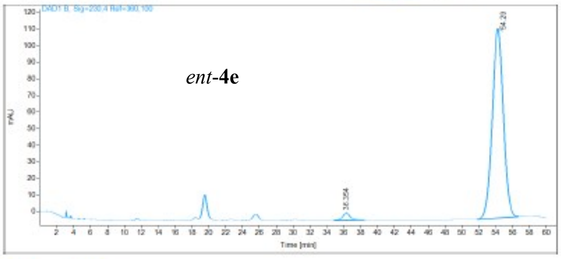

Name PC 816 L

RT [min]	Type	Area%	Area	Height	Width [min]
36.35	BB	2.54	271.62	4.19	0.95
54.29	BB	97.46	10411.30	113.98	1.41
	Sum	100.00	10682.91		

Sample name: PC 816+821N

Data file: C:\SNOOPY\PC\816821NYIC.D
Description: Laufmittel: n-Heptan/iPrOH 9:1;
Probe ist in LM/DCM gelöst
Injection date: 11/25/2014 2:57:22 PM
Acq. Analysis method: CHIRALPAKIC1-6LNP.M

Column: Chiralpak IC, (150 x 4,6) mm, 5µ, SN: IC00CD-QF015

Pressure at start: 26 bar **Start flow:** 0.700 ml/min **Column oven:** 29.99 °C

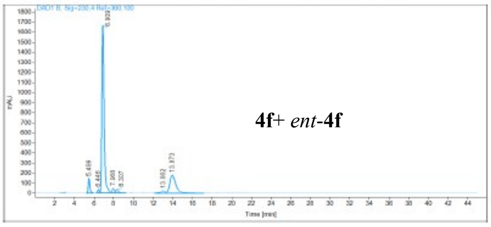

4f+ *ent*-**4f**

Name PC 816+821N

RT [min]	Type	Area%	Area	Height	Width [min]
5.50	BB	4.43	1681.12	143.46	0.18
6.45	VV	1.25	473.77	36.70	0.20
6.91	VV	68.53	25981.00	1665.22	0.24
7.97	VV	2.05	778.44	43.26	0.27
8.31	VB	2.11	801.79	36.30	0.32
13.06	BV	1.85	700.58	20.99	0.49
13.97	VB	19.77	7494.91	178.31	0.63
	Sum	100.00	37911.62		

Sample name: PC 821 N

Data file: C:\SNOOPY\PC\821NYIC.D
Description: Laufmittel: n-Heptan/iPrOH 9:1;
Probe ist in LM/DCM gelöst
Injection date: 11/25/2014 4:29:35 PM
Acq. Analysis method: CHIRALPAKIC1-6LNP.M

Column: Chiralpak IC, (150 x 4,6) mm, 5µ, SN: IC00CD-QF015

Pressure at start: 26 bar **Start flow:** 0.700 ml/min **Column oven:** 29.99 °C

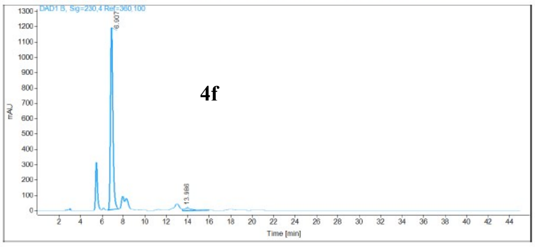

4f

Name PC 821 N

RT [min]	Type	Area%	Area	Height	Width [min]
6.91	BB	95.63	18099.65	1189.18	0.23
13.99	VB	4.37	827.49	17.75	0.69
	Sum	100.00	18927.14		

Sample name: PC 816 N

Data file: C:\SNOOPY\PC\816NYIC.D
Description: Laufmittel: n-Heptan/iPrOH 9:1;
Probe ist in LM/DCM gelöst
Injection date: 11/25/2014 3:43:29 PM
Acq. Analysis method: CHIRALPAKIC1-6LNP.M

Column: Chiralpak IC, (150 x 4,6) mm, 5µ, SN: IC00CD-QF015

Pressure at start: 26 bar **Start flow:** 0.700 ml/min **Column oven:** 30 °C

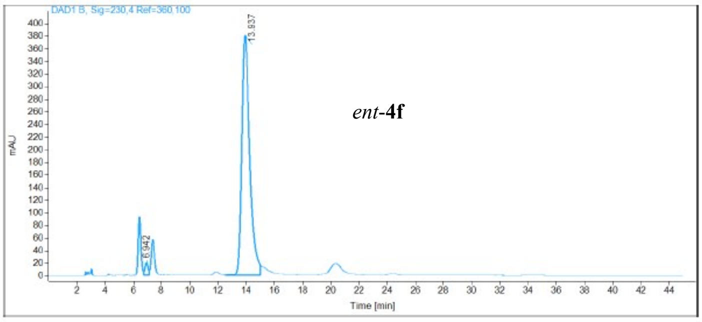

ent-**4f**

Name PC 816 N

RT [min]	Type	Area%	Area	Height	Width [min]
6.94	VV	2.01	308.25	20.40	0.23
13.94	MF	97.99	15050.47	380.63	0.66
	Sum	100.00	15358.72		

Sample Name: PC 816 C
Data file: D:\GONZO\PC\816CRAD.D
Sample Info: Laufmittel: n-Heptan/IP 7:3;
Die Probe ist in LM/DCM gelöst

HEWLETT PACKARD

Säule: DAICELAD.M
Säuleninfo: Chiralpak AD (250x4,6)mm
Operator: Analytik Labor AKEN

Injektion Time: 20:01:16
Injektion Date: 23.09.2014

Instrument Conditions:	At Start	At Stop
Temperature in°C:	30.0°C	30.0°C
Pressure in bar:	33.5	34.3
Flow in ml/min:	1.00	1.00

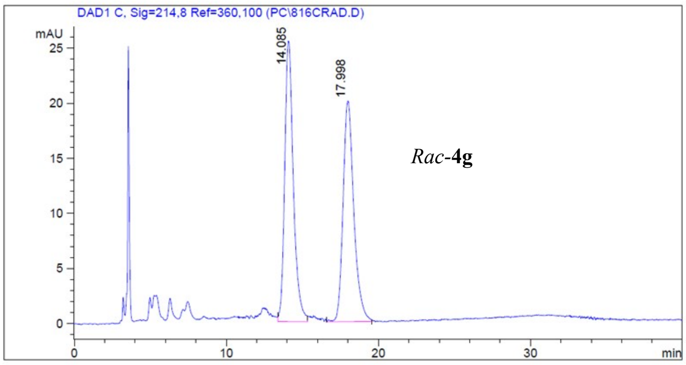

#	Ret. Time (min)	Width	Height (mAU)	Area (mAU*s)	Area %
1	14.09	0.55	25.48	1005.96	50.52
2	18.00	0.65	20.02	985.10	49.48
Total				1991.06	100.00

Sample Name: PC 821 C
Data file: D:\GONZO\PC\821CAD.D
Sample Info: Laufmittel: n-Heptan/iPrOH 7:3;
Die Probe ist in DCM/LM gelöst.

HEWLETT PACKARD

Säule: DAICELAD.M
Säuleninfo: Chiralpak AD (250x4,6)mm
Operator: Analytik Labor AKEN

Injektion Time: 14:19:47
Injektion Date: 17.11.2014

Instrument Conditions:	At Start	At Stop
Temperature in°C:	30.0°C	30.0°C
Pressure in bar:	34.5	35.3
Flow in ml/min:	1.00	1.00

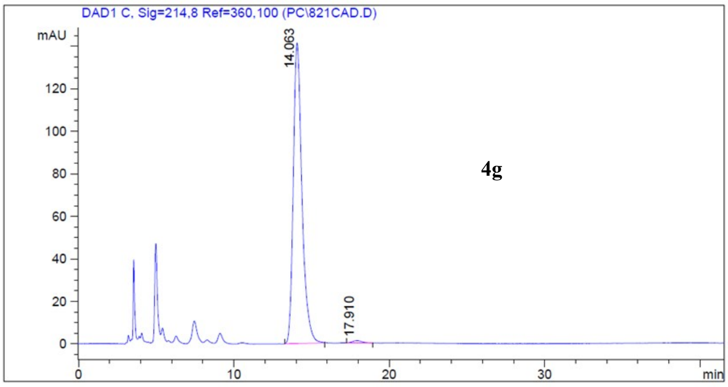

#	Ret. Time (min)	Width	Height (mAU)	Area (mAU*s)	Area %
1	14.06	0.59	141.41	5495.21	99.16
2	17.91	0.51	1.09	46.42	0.84
Total				5541.63	100.00

```
Sample Name:        PC 816 A
Data file:          D:\GONZO\PC\816ARAD.D
Sample Info:        Laufmittel: n-Heptan/IP  7:3;
                    Die Probe ist in LM/DCM gelöst

Säule:              DAICELAD.M
Säuleninfo:         Chiralpak AD (250x4,6)mm
Operator:           Analytik Labor AKEN

 Injektion Time:     19:20:04
 Injektion Date:     23.09.2014

 Instrument Conditions:   At Start          At Stop
 Temperature in°C:          30.0°C           30.0°C
 Pressure in bar:           33.3             34.3
 Flow in ml/min:            1.00             1.00
```

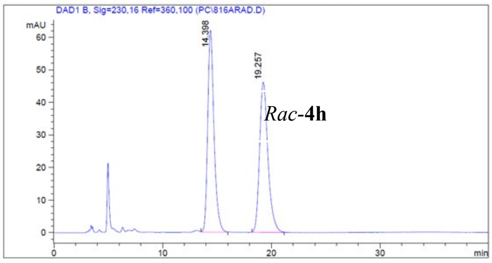

#	Ret. Time (min)	Width	Height (mAU)	Area (mAU*s)	Area %
1	14.40	0.61	62.04	2491.52	50.14
2	19.26	0.81	46.17	2477.41	49.86
Total				4968.93	100.00

```
Sample Name:        PC 821 A
Data file:          D:\GONZO\PC\821AAD.D
Sample Info:        Laufmittel: n-Heptan/iPrOH  7:3;
                    Die Probe ist in LM/DCM gelöst

Säule:              DAICELAD.M
Säuleninfo:         Chiralpak AD (250x4,6)mm
Operator:           Analytik Labor AKEN

 Injektion Time:     15:58:19
 Injektion Date:     10.11.2014

 Instrument Conditions:   At Start          At Stop
 Temperature in°C:          30.0°C           30.0°C
 Pressure in bar:           34.3             35.3
 Flow in ml/min:            1.00             1.00
```

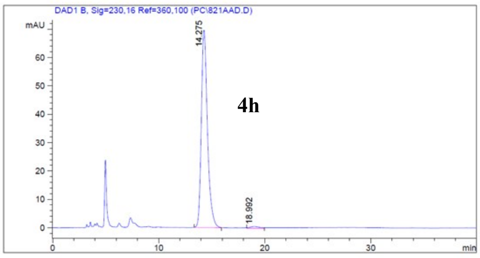

#	Ret. Time (min)	Width	Height (mAU)	Area (mAU*s)	Area %
1	14.27	0.60	69.61	2787.92	98.64
2	18.99	1.04	0.61	38.46	1.36
Total				2826.38	100.00

```
Sample Name:        PC 821 A1
Data file:          D:\GONZO\PC\821A1AD.D
Sample Info:        Laufmittel: n-Heptan/iPrOH  7:3;
                    Die Probe ist in LM/DCM gelöst

Säule:              DAICELAD.M
Säuleninfo:         Chiralpak AD (250x4,6)mm
Operator:           Analytik Labor AKEN

 Injektion Time:     11:44:53
 Injektion Date:     20.11.2014

 Instrument Conditions:   At Start          At Stop
 Temperature in°C:          30.0°C           30.0°C
 Pressure in bar:           34.3             35.0
 Flow in ml/min:            1.00             1.00
```

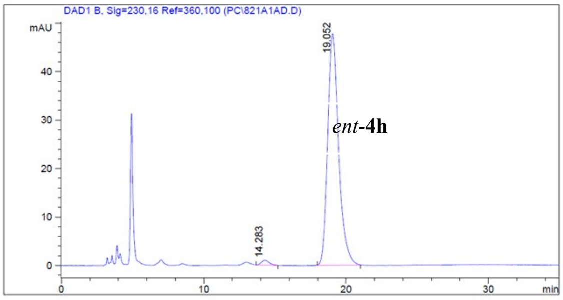

#	Ret. Time (min)	Width	Height (mAU)	Area (mAU*s)	Area %
1	14.28	0.49	1.06	41.37	1.59
2	19.05	0.82	47.79	2553.57	98.41
Total				2594.93	100.00

```
Sample Name:        PC 816 B rac
Data file:          D:\GONZO\PC\816BR2OD.D
Sample Info:        Laufmittel: n-Heptan/EtOH  9:1;
                    Die Probe ist in DCM/LM gelöst

Säule:              DAICELOD.M
Säuleninfo:         Chiralcel OD (250x4,6)mm
Operator:           Analytik Labor AKEN

Injektion Time:     10:33:20
Injektion Date:     24.10.2014

Instrument Conditions:   At Start      At Stop
Temperature in°C:         30.0°C        30.0°C
Pressure in bar:          29.4          29.8
Flow in ml/min:           1.00          1.00
```

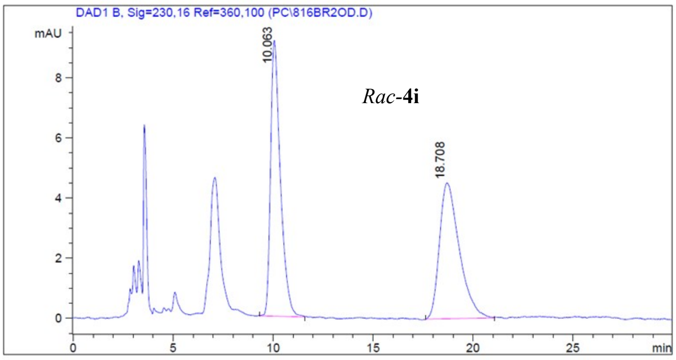

#	Ret. Time (min)	Width	Height (mAU)	Area (mAU*s)	Area %
1	10.06	0.51	9.16	313.43	50.13
2	18.71	1.04	4.51	311.86	49.87
Total				625.28	100.00

```
Sample Name:        PC 821 B
Data file:          D:\GONZO\PC\821BOD.D
Sample Info:        Laufmittel: n-Heptan/iPrOH  9:1;
                    Die Probe ist in DCM/LM gelöst

Säule:              DAICELOD.M
Säuleninfo:         Chiralcel OD (250x4,6)mm
Operator:           Analytik Labor AKEN

Injektion Time:     08:19:03
Injektion Date:     24.11.2014

Instrument Conditions:   At Start      At Stop
Temperature in°C:         30.0°C        30.0°C
Pressure in bar:          29.9          30.3
Flow in ml/min:           1.00          1.00
```

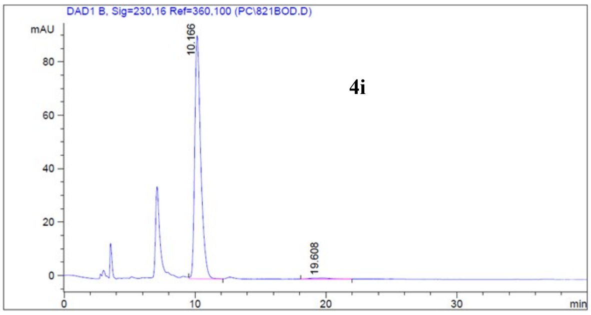

#	Ret. Time (min)	Width	Height (mAU)	Area (mAU*s)	Area %
1	10.17	0.48	90.98	2910.53	98.54
2	19.61	1.29	0.41	43.07	1.46
Total				2953.60	100.00

Sample Name: PC 816 E rac
Data file: D:\GONZO\PC\816ER2OD.D
Sample Info: Laufmittel: n-Heptan/EtOH 9:1;
Die Probe ist in DCM/LM gelöst

HEWLETT PACKARD

Säule: DAICELOD.M
Säuleninfo: Chiralcel OD (250x4,6)mm
Operator: Analytik Labor AKEN

Injektion Time: 11:04:33
Injektion Date: 24.10.2014

Instrument Conditions:	At Start	At Stop
Temperature in°C:	30.0°C	30.0°C
Pressure in bar:	29.2	29.6
Flow in ml/min:	1.00	1.00

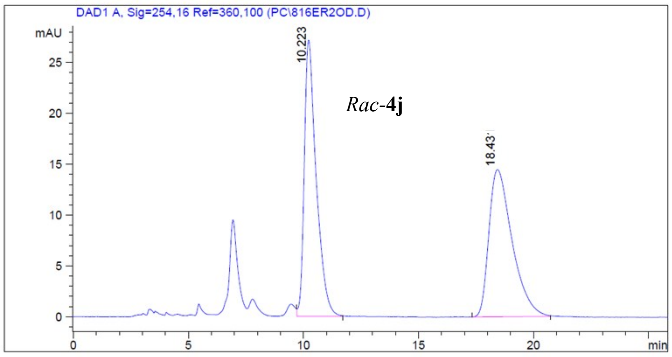

#	Ret. Time (min)	Width	Height (mAU)	Area (mAU*s)	Area %
1	10.22	0.52	27.18	979.52	49.03
2	18.43	1.06	14.48	1018.10	50.97
Total				1997.62	100.00

Sample Name: PC 821 E
Data file: D:\GONZO\PC\821EOD.D
Sample Info: Laufmittel: n-Heptan/EtOH 9:1;
Die Probe ist in DCM/LM gelöst.

HEWLETT PACKARD

Säule: DAICELOD.M
Säuleninfo: Chiralcel OD (250x4,6)mm
Operator: Analytik Labor AKEN

Injektion Time: 10:17:09
Injektion Date: 18.11.2014

Instrument Conditions:	At Start	At Stop
Temperature in°C:	30.0°C	30.0°C
Pressure in bar:	29.7	30.2
Flow in ml/min:	1.00	1.00

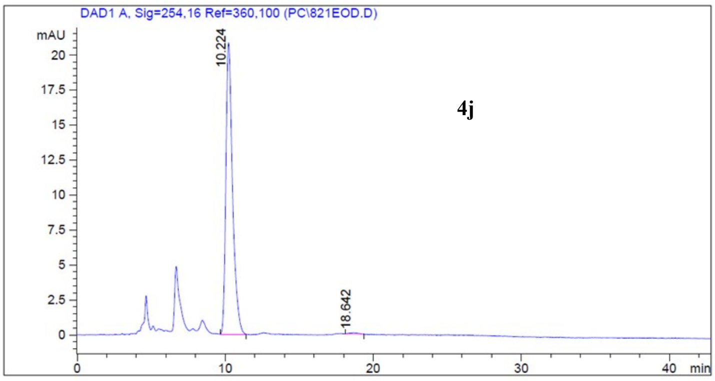

#	Ret. Time (min)	Width	Height (mAU)	Area (mAU*s)	Area %
1	10.22	0.48	20.85	656.61	99.39
2	18.64	0.70	0.10	4.01	0.61
Total				660.62	100.00

Sample name: **PC 816 F rac**
Data file: C:\SNOOPY\PC\PC 816 F RAC IC.D
Description: Laufmittel: n-Heptan/iPrOH 9 :1 Die Probe ist DCM/LM gelöst.

Injection date: 11/26/2014 8:27:48 AM
Acq. Analysis method: CHIRALPAKIC1-6LNP.M

Column: Chiralpak IC, (150 x 4,6) mm, 5µ, SN: IC00CD-QF015

Pressure at start: 26 bar **Start flow:** 0.700 ml/min **Column oven:** 30 °C

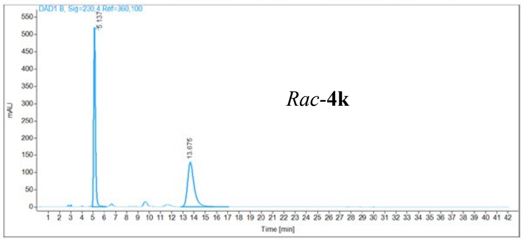

Name PC 816 F rac

RT [min]	Type	Area%	Area	Height	Width [min]
5.14	VV	50.40	5403.85	518.84	0.16
13.68	BB	49.60	5317.10	129.58	0.62
	Sum	100.00	10720.96		

Sample name: **PC 821 F**
Data file: C:\SNOOPY\PC\821FIC.D
Description: Laufmittel: n-Heptan/iPrOH 9:1; Probe ist in LM/DCM gelöst

Injection date: 11/27/2014 8:03:07 AM
Acq. Analysis method: CHIRALPAKIC1-6LNP.M

Column: Chiralpak IC, (150 x 4,6) mm, 5µ, SN: IC00CD-QF015

Pressure at start: 27 bar **Start flow:** 0.700 ml/min **Column oven:** 29.99 °C

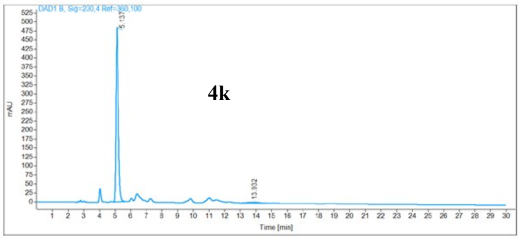

Name PC 821 F

RT [min]	Type	Area%	Area	Height	Width [min]
5.14	BB	97.60	4970.08	485.03	0.16
13.93	BB	2.40	122.25	2.50	0.70
	Sum	100.00	5092.34		

Sample name: **PC 821 F (QD)**
Data file: C:\SNOOPY\PC\821FQDIC.D
Description: Laufmittel: n-Heptan/iPrOH 9:1; Probe ist in LM/DCM gelöst

Injection date: 11/27/2014 8:34:36 AM
Acq. Analysis method: CHIRALPAKIC1-6LNP.M

Column: Chiralpak IC, (150 x 4,6) mm, 5µ, SN: IC00CD-QF015

Pressure at start: 27 bar **Start flow:** 0.700 ml/min **Column oven:** 29.99 °C

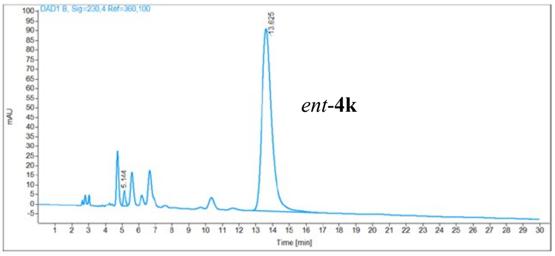

Name PC 821 F (QD)

RT [min]	Type	Area%	Area	Height	Width [min]
5.14	VV	1.96	77.68	7.81	0.15
13.63	VB	98.04	3884.73	94.70	0.62
	Sum	100.00	3962.41		

Sample Name: PC 816 G rac
Data file: D:\GONZO\PC\816GR1AD.D
Sample Info: Laufmittel: n-Heptan/iPrOH 7:3;
Die Probe ist in LM/DCM gelöst

HEWLETT PACKARD

Säule: DAICELAD.M
Säuleninfo: Chiralpak AD (250x4,6)mm
Operator: Analytik Labor AKEN

Injektion Time: 14:09:43
Injektion Date: 20.11.2014

Instrument Conditions:	At Start	At Stop
Temperature in°C:	30.0°C	30.0°C
Pressure in bar:	34.3	35.0
Flow in ml/min:	1.00	1.00

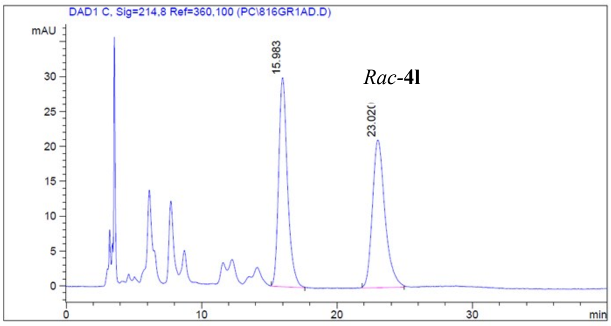

#	Ret. Time (min)	Width	Height (mAU)	Area (mAU*s)	Area %
1	15.98	0.68	29.97	1359.98	49.82
2	23.02	0.95	21.17	1369.74	50.18
Total				2729.72	100.00

Sample Name: PC 821 G
Data file: D:\GONZO\PC\821GADAD.D
Sample Info: Laufmittel: n-Heptan/EtOH 7:3;
Die Probe ist in DCM/LM gelöst.

HEWLETT PACKARD

Säule: DAICELAD.M
Säuleninfo: Chiralpak AD (250x4,6)mm
Operator: Analytik Labor AKEN

Injektion Time: 13:13:13
Injektion Date: 21.11.2014

Instrument Conditions:	At Start	At Stop
Temperature in°C:	30.0°C	30.0°C
Pressure in bar:	34.4	34.8
Flow in ml/min:	1.00	1.00

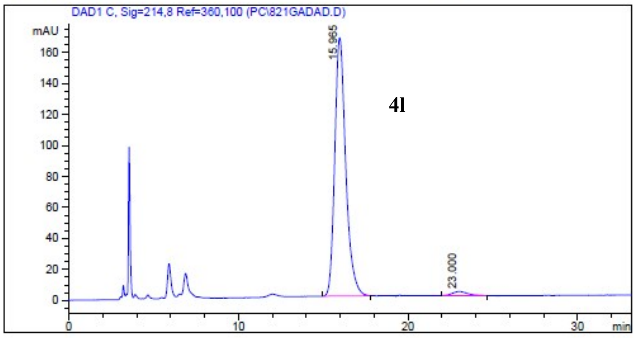

#	Ret. Time (min)	Width	Height (mAU)	Area (mAU*s)	Area %
1	15.96	0.69	166.71	7514.89	97.75
2	23.00	0.76	2.70	172.71	2.25
Total				7687.60	100.00

Sample name: **PC 816 H rac**

Data file: C:\SNOOPY\PC\816HRIC.D
Description: Laufmittel: n-Heptan/iPrOH 9:1;
Probe ist in LM/DCM gelöst

Injection date: 11/24/2014 12:36:57 PM
Acq. Analysis method: CHIRALPAKIC1-6LNP.M

Column: Chiralpak IC, (150 x 4,6) mm, 5µ, SN: IC00CD-QF015

Pressure at start: 26 bar **Start flow:** 0.700 ml/min **Column oven:** 29.99 °C

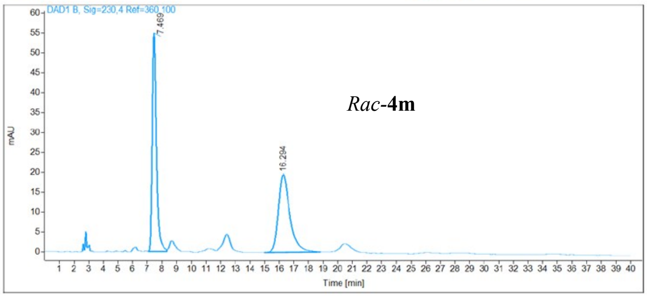

Name PC 816 H rac

RT [min]	Type	Area%	Area	Height	Width [min]
7.47	BV	49.72	1059.69	55.04	0.29
16.29	BB	50.28	1071.55	19.54	0.83
	Sum	100.00	2131.23		

Sample name: **PC 821 H**

Data file: C:\SNOOPY\PC\821HIC.D
Description: Laufmittel: n-Heptan/iPrOH 9:1;
Probe ist in LM/DCM gelöst

Injection date: 11/24/2014 4:13:47 PM
Acq. Analysis method: CHIRALPAKIC1-6LNP.M

Column: Chiralpak IC, (150 x 4,6) mm, 5µ, SN: IC00CD-QF015

Pressure at start: 26 bar **Start flow:** 0.700 ml/min **Column oven:** 29.99 °C

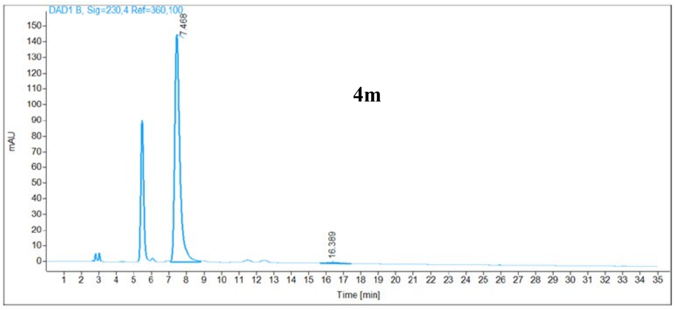

Name PC 821 H

RT [min]	Type	Area%	Area	Height	Width [min]
7.47	VV	98.71	2881.33	144.88	0.30
16.39	BB	1.29	37.53	0.77	0.63
	Sum	100.00	2918.87		

Sample name: **SM 58b + 59b**

Data file: C:\SNOOPY\SM\5859BNIC.D
Description: Laufmittel: n-Heptan/EtOH 9:1;
Die Probe ist im LM/DCM gelöst.

Injection date: 4/17/2014 1:10:44 PM
Acq. Analysis method: CHIRALPAKIC1-6LNP.M

Column: Chiralpak IC, (150 x 4,6) mm, 5µ, SN: IC00CD-QF015

Pressure at start: 24 bar **Start flow:** 0.700 ml/min **Column oven:** 30 °C

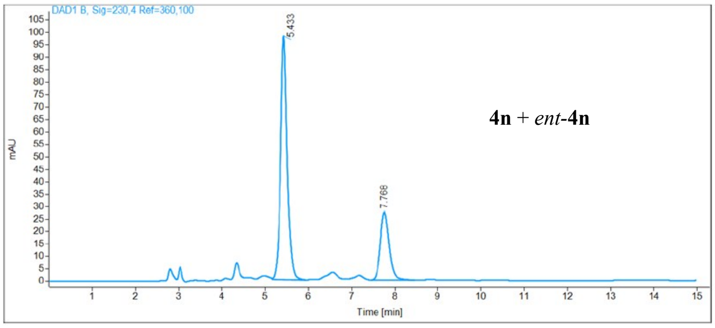

Name SM 58b + 59b

RT [min]	Type	Area%	Area	Height	Width [min]
5.43	VB	72.67	1033.19	97.76	0.16
7.77	BB	27.33	388.61	27.06	0.22
	Sum	100.00	1421.79		

Sample name: **PC 819**

Data file: C:\SNOOPY\PC\PC 819 IC.D
Description: Laufmittel: n-Heptan/EtOH 9:1 Die Probe ist DCM/LM gelöst.

Injection date: 11/3/2014 9:11:21 AM
Acq. Analysis method: CHIRALPAKIC1-6LNP.M

Column: Chiralpak IC, (150 x 4,6) mm, 5µ, SN: IC00CD-QF015

Pressure at start: 24 bar **Start flow:** 0.700 ml/min **Column oven:** 24.22 °C

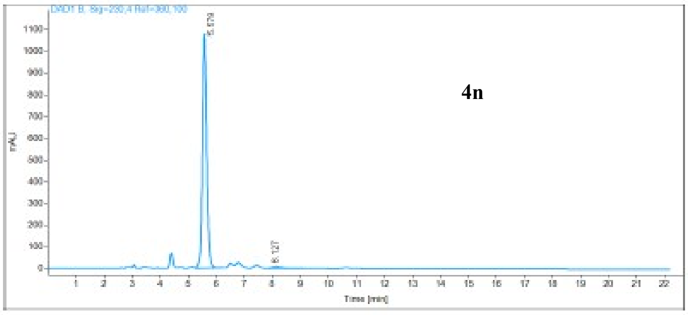

Name PC 819

RT [min]	Type	Area%	Area	Height	Width [min]
5.58	VB	98.42	10983.01	1081.63	0.15
8.13	BB	1.58	175.97	7.57	0.37
	Sum	100.00	11158.98		

Sample name: **SM 58c + 59c**

Data file: C:\SNOOPY\SM\SM 58C + 59C IC.D
Description: Laufmittel: n-Heptan/EtOH 97:3 Die Probe ist DCM/LM gelöst.

Injection date: 4/15/2014 1:22:37 PM
Acq. Analysis method: CHIRALPAKIC1-6LNP.M

Column: Chiralpak IC, (150 x 4,6) mm, 5µ, SN: IC00CD-QF015

Pressure at start: 24 bar **Start flow:** 0.700 ml/min **Column oven:** 29.97 °C

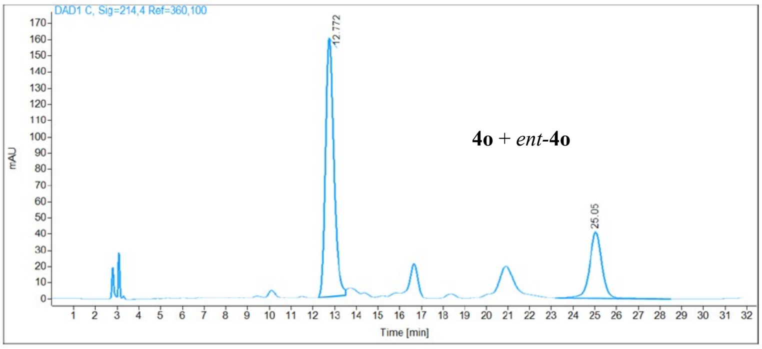

Name SM 58c + 59c

RT [min]	Type	Area%	Area	Height	Width [min]
12.77	BV	71.89	4179.24	159.53	0.40
25.05	BB	28.11	1633.96	40.59	0.61
	Sum	100.00	5813.21		

Sample name: **PC 58 C rep2**

Data file: C:\SNOOPY\PC\58CREP2XIC.D
Description: Laufmittel: n-Heptan/EtOH 97:3;
Probe ist in LM/DCM gelöst

Injection date: 11/18/2014 6:18:37 PM
Acq. Analysis method: CHIRALPAKIC1-6LNP.M

Column: Chiralpak IC, (150 x 4,6) mm, 5µ, SN: IC00CD-QF015

Pressure at start: 23 bar **Start flow:** 0.700 ml/min **Column oven:** 29.98 °C

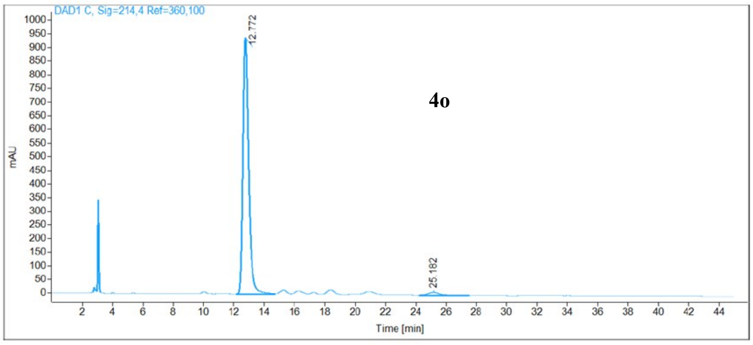

Name PC 58 C rep2

RT [min]	Type	Area%	Area	Height	Width [min]
12.77	BV	98.02	24348.06	938.63	0.39
25.18	BB	1.98	490.94	9.88	0.77
	Sum	100.00	24839.00		
